# Rapid access to substituted 2-naphthyne intermediates *via* the benzannulation of halogenated silylalkynes†
†Electronic supplementary information (ESI) available: Synthetic procedures, characterization data, and DFT calculations. CCDC 1539450 and 1539451. For ESI and crystallographic data in CIF or other electronic format see DOI: 10.1039/c7sc01625e



**DOI:** 10.1039/c7sc01625e

**Published:** 2017-06-09

**Authors:** Samuel J. Hein, Dan Lehnherr, William R. Dichtel

**Affiliations:** a Department of Chemistry , Northwestern University , 2145 Sheridan Road , Evanston , Illinois 60208 , USA . Email: wdichtel@northwestern.edu; b Department of Chemistry and Chemical Biology , Baker Laboratory , Cornell University , Ithaca , New York 14853 , USA

## Abstract

Aryne intermediates are versatile and important reactive intermediates for natural product and polymer synthesis. 2-Naphthynes are relatively unexplored because few methods provide precursors to these intermediates, especially for those bearing additional substituents. Here we report a general synthetic strategy to access 2-naphthyne precursors through an Asao-Yamamoto benzannulation of *ortho*-(phenylethynyl)benzaldehydes with halo-silylalkynes. This transformation provides 2-halo-3-silylnaphthalenes with complete regioselectivity. These naphthalene products undergo desilylation/dehalogenation in the presence of F^–^ to generate the corresponding 2-naphthyne intermediate, as evidenced by furan trapping experiments. When these 2-naphthynes are generated in the presence of a copper catalyst, *ortho*-naphthalene oligomers, trinaphthalene, or binaphthalene products are formed selectively by varying the catalyst loading and reaction temperature. The efficiency, mild conditions, and versatility of the naphthalene products and naphthyne intermediates will provide efficient access to many new functional aromatic systems.

## Abstract 2

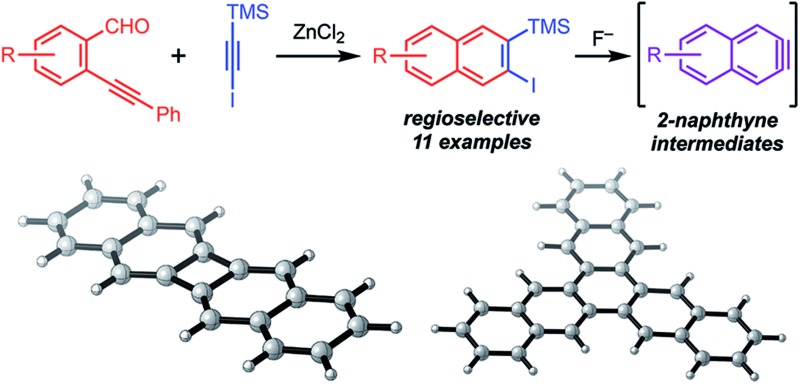
A ZnCl_2_-catalyzed, regioselective benzannulation of halogenated silylacetylenes provides access to 2-naphthyne precursors.

## 

*ortho*-Arynes are reactive intermediates generated through the elimination of adjacent functional groups from an aromatic system.^1^ These species have a rich history in physical and synthetic organic chemistry since their existence was first postulated by Stoermer and Kahlert.^2^ Transformations involving nucleophilic additions, bond insertions, pericyclic reactions, and multicomponent coupling reactions involving arynes have been employed for the synthesis of natural products and polycyclic aromatic hydrocarbons.^3^ Although early arynes were first generated using strong bases with limited functional group compatibility, Kobayashi reported that *ortho*-silylaryl triflates generate arynes efficiently in the presence of fluoride ions.^4^ The mild conditions and excellent functional group tolerance of this approach has led to *ortho*-silylaryl triflates becoming popular aryne precursors. Yet few *ortho*-silylaryl triflates are commercially available, and their synthesis is often laborious, despite the recent development of cycloaddition-based strategies by Harrity^5^ and aryl C–H bond silylations by Daugulis.^6^

Arynes derived from other aromatic systems^7,8^ enable the synthesis of more complex structures, yet access to appropriately substituted precursors is even more limited. Wong^9^ and Maly^10^ reported substituted 2-naphthyne precursors, but the seven step syntheses of each method limit general adoption (Scheme 1a and b). We recently noted that the Asao-Yamamoto benzannulation,^11^ which was known to provide 2,3-diarylnaphthalenes from *o*-phenylethynyl-benzaldehydes and diarylalkynes, is capable of modifying conjugated polymer backbones with high efficiency^12^ and is regioselective for alkynes with electronically different aryl substituents.^13^ The scope of these benzannulation reactions has since been expanded to silyl- and haloalkynes, enabling the synthesis of sterically hindered^14^ and polyheterohalogenated naphthalenes.^15^ Here we demonstrate a new variant of the Asao-Yamamoto benzannulation that provides 2-silyl-3-halonaphthalenes as single regioisomers (Scheme 1c). These compounds generate 2-naphthyne intermediates under mild conditions, which were trapped by furan, oligomerized, and found to undergo cyclodimerization or cyclotrimerization to bi- and trinaphthalenes, respectfully. Several examples featuring substituted *o*-phenylethynylbenzaldehydes demonstrate the generality of this approach to access substituted 2-naphthyne intermediates.

**Scheme 1:**
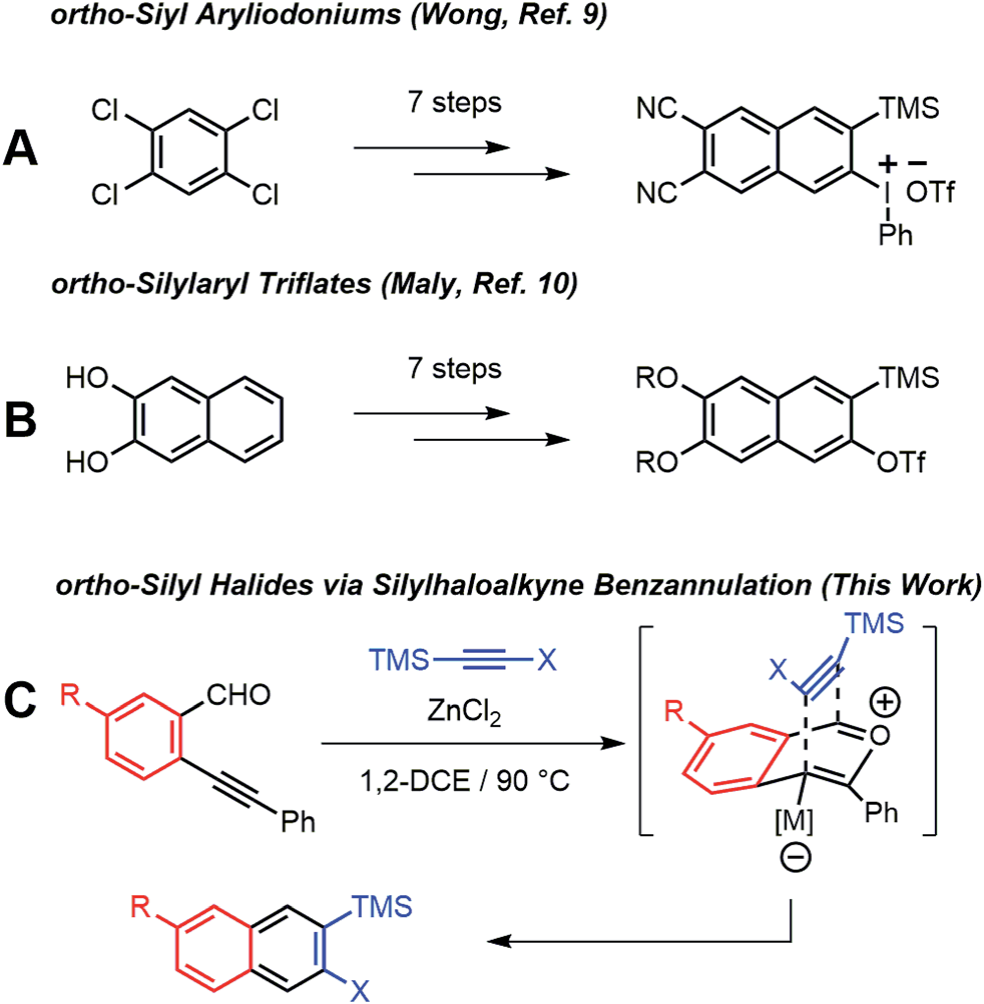
Synthesis of substituted 2-naphthyne precursors.

We evaluated the scope and regioselectivity of the benzannulation reaction using substituted benzaldehydes and silylhaloalkynes (Fig. 1). Previously, catalytic Cu(OTf)_2_ in the presence of CF_3_CO_2_H had been employed for the benzannulation of substituted acetylenes, but these conditions favour protodesilylation of the trimethylsilyl (TMS) substituent.^14^ ZnCl_2_ is a milder catalyst that does not require acidic additives.^16^ Naphthalene products **3a–j** were obtained as single regioisomers, often in good to excellent yield. The reaction was more efficient for iodoalkynes than bromoalkynes, as shown for benzaldehyde **1b** with **2a** (X = I, 93% yield) and **2b** (X = Br, 18% yield). Likewise, the reaction of **1c** with **2a** and **2b** provided **3d** (93%) and **3e** (42%). Unsubstituted and halogenated benzaldehydes (**1a–f**) performed well with yields between 70–93% whereas those for benzaldehydes bearing two phenolic esters (**1g**) and two alkyl groups (**1h**) were 63% and 59%, respectively. Benzaldehydes bearing stronger electron donating groups, such as dimethoxy benzaldehyde **1i**, did not undergo benzannulation, and a mixture of aldehyde decomposition products and unreacted alkyne was obtained.

**Fig. 1:**
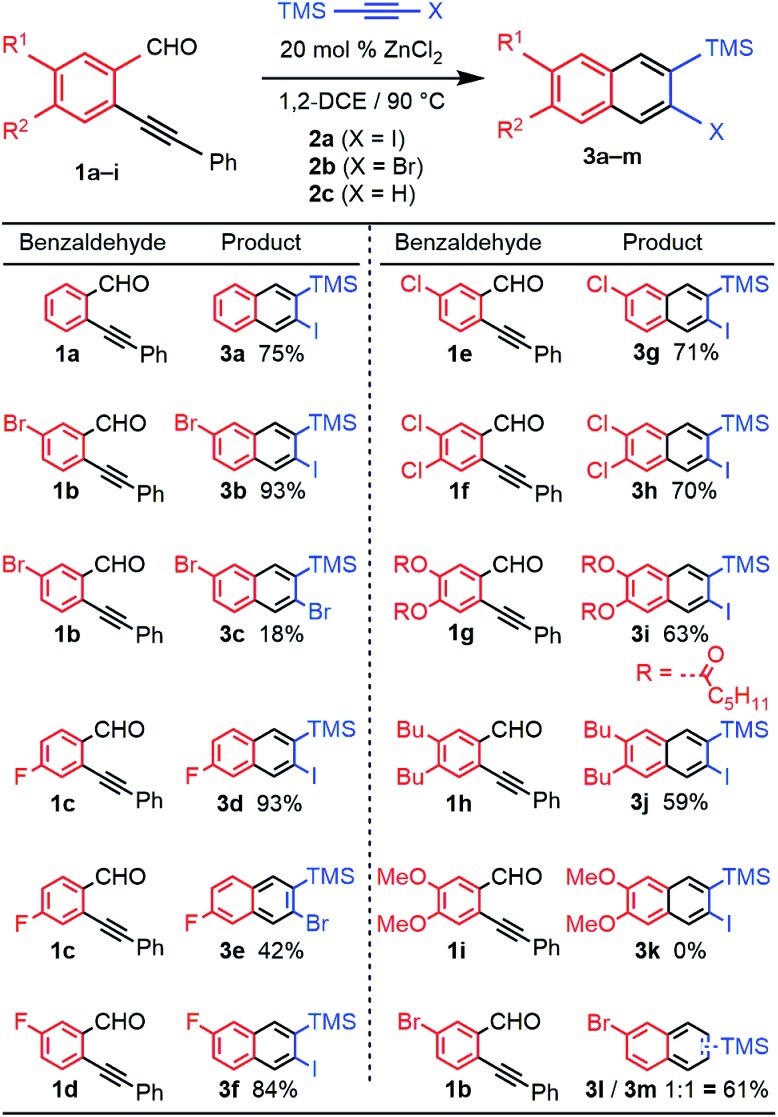
Synthesis of 2-naphthyne precursors from substituted 2-(phenylethynyl)benzaldehydes all yields are isolated yields. Reaction conditions: alkyne (**2a–c**, 0.10 M in 1,2-DCE); benzaldehyde (**1a**, 2.0 equiv. or **1b–i**,1.3 equiv.); ZnCl_2_ (0.20 equiv.).

The benzannulation reactions of **2a** and **2b** (Fig. 1) provide substituted naphthalene products as single regioisomers when non-pseudosymmetric benzaldehydes (*e.g.*, **1c**, **1d**) are employed. Our previous studies of diarylalkynes and haloarylalkynes indicated that regioselectivity arises from the ability of the alkyne substituents to preferentially stabilize developing positive charge at one of the alkyne carbons.^13,15^ For example, when brominated benzaldehyde **1b** undergoes benzannulation with an aryl-haloalkyne, using either Cu(OTf)_2_ or ZnCl_2_ catalysts, the *syn*-regioisomer with respect to the iodine and bromine positions is obtained (Fig. 2a). The opposite regioisomer is obtained for silylhaloalkyne substrates; **1b** reacts with **2a** and **2b** to provide the corresponding *anti*-regioisomers **3b** and **3c**, respectively. These outcomes were confirmed by single crystal X-ray crystallography of **3c** (Fig. 2a), compared to that obtained for a typical arylhaloalkyne. The regiochemical outcome is insensitive to substitution patterns on the benzaldehyde, as demonstrated for the benzannulations of monofluorinated benzaldehyde regioisomers **1c** and **1d** with **2a**. Each reaction provides a single fluoronaphthalene regioisomer, whose structures were assigned by ^19^F and 2D NMR spectroscopy (see ESI†). Finally, halogenation of the silylalkyne is also essential for regioselectivity, as the benzannulation reaction of trimethylsilylacetylene (**2c**) and **1b** provides a nearly 1 : 1 ratio of the two regioisomers. The regiochemical outcome of these ZnCl_2_-catalyzed benzannulations with silylarylalkynes are congruent with those using silylhaloalkynes. This observation supports our hypothesis that the preferred regiochemical outcome is based on going through the more stabilized regioisomeric cation, in this case the cation stabilized by both the beta-silyl effect and formation of a benzylic cation (see ESI†).

**Fig. 2:**
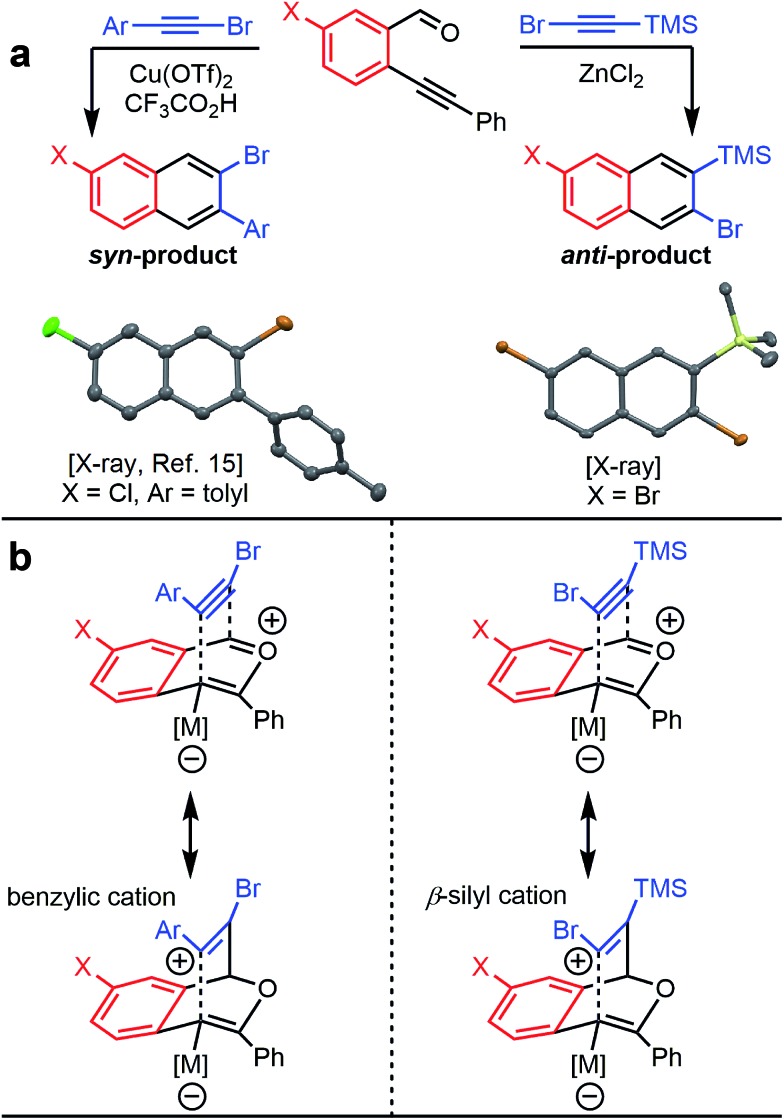
(a) Haloarylalkynes and halosilylalkynes provide opposite regioselectivity in benzannulation reactions, as demonstrated by X-ray crystallography.^17^ Ellipsoids set to 50% probability level for **3c**. (b) Rationale for the regioselectivity of each reaction. The silicon substituent stabilizes developing positive charge on the carbon adjacent to the halogen, which makes the observed regioselectivity consistent with other Asao-Yamamoto benzannulation reactions.

The reversed regioselectivity of halosilylalkyne benzannulations likely originates from the combined ability of the silicon and halogen substituents to stabilize developing positive charge on the alkyne carbon beta to the silicon atom.^18^ In contrast, when arylhaloalkynes are benzannulated, the aromatic ring stabilizes a developing positive charge on the alkyne carbon alpha to the ring (Fig. 2b). A DFT model of the proposed transition states leading to either the *syn*- or *anti*-regioisomer correctly predicts the reversal in regioselectivity observed experimentally between silylhaloalkynes and arylhaloalkynes (Fig. 3). The B3LYP/6-31G(d) calculated ZPE-corrected electronic energies of regioisomeric transition states predict a 2.0 kcal mol^–1^ preference for the *anti*-regioisomer in the case of silylhaloalkyne **2b** compared to 5.6 kcal mol^–1^ in favor of the *syn*-regioisomer for phenylbromoalkyne. An alternate mechanism in which the metal is not bound to the benzopyrylium intermediate is also plausible, and transition state energies of those structures also support the observed regioselectivity (see ESI†).

**Fig. 3:**
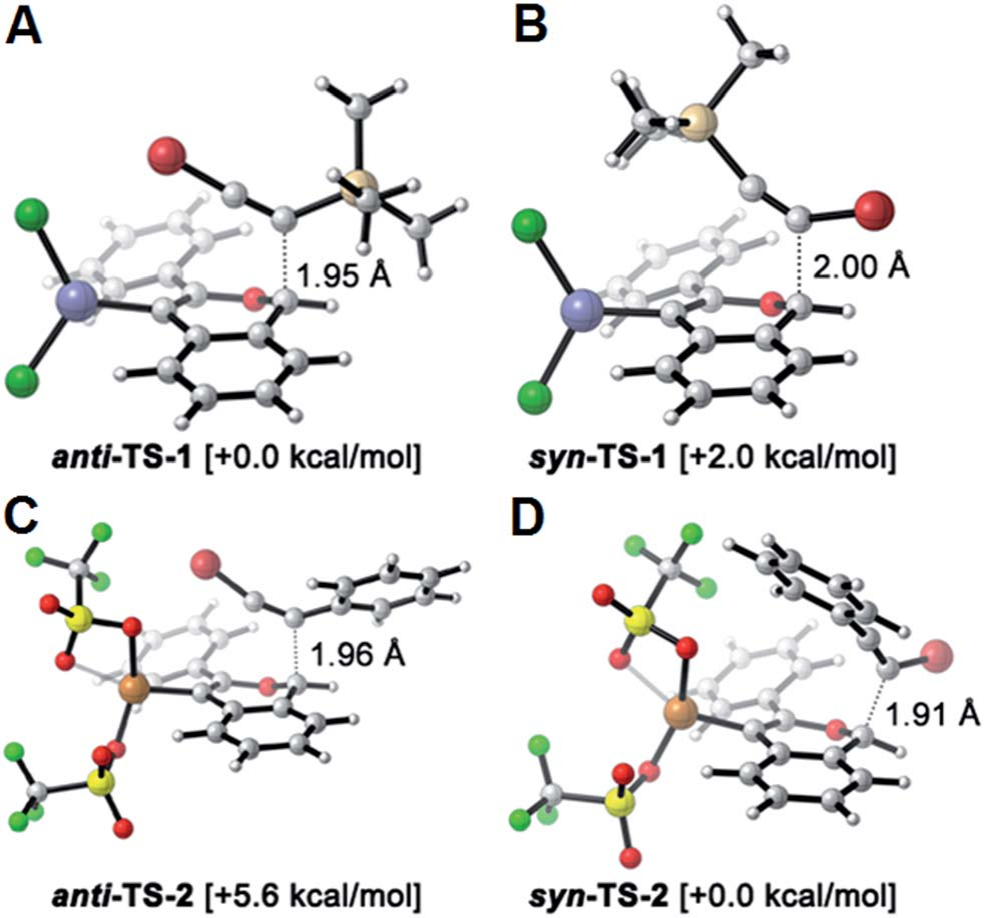
DFT calculated transition-states using B3LYP/6-31G(d) potentially responsible for the regioselectivity outcome in the benzannulation of: (A, B) silylhaloalkynes & (C, D) arylhaloalkynes, along with their relative electronic energies and bond forming interatomic distances. Element coloring scheme: C = silver, H = white, O = light red, Zn = blue, Cl = dark green, F = light green, S = yellow, Br = dark red, Cu = bronze.

Furthermore, we had previously accessed a library of polyheterohalogenated naphthalenes through the benzannulation of arylhaloalkynes. Although this approach was used to prepare more than twenty polyheterohalogenated naphthalenes as single regioisomers, it was limited to 2-arylnaphthalene derivatives.^15^ Silylhaloalkyne benzannulations eliminate this restriction, and instead provide 2-trimethylsilylnaphthalenes with a silyl group that is easily removed or further transformed. For example, treatment of **3c** with ICl afforded polyheterohalogenated naphthalene **4** in quantitative yield (Scheme 2). The TMS group incorporated into the naphthalene products serves as a versatile handle for further functionalization, as C(aryl)–Si bonds are readily transformed into I, Br, Cl, H, OH, CF_3_, Me, and others, highlighting the vast chemical space available through this method.^19^

**Scheme 2:**
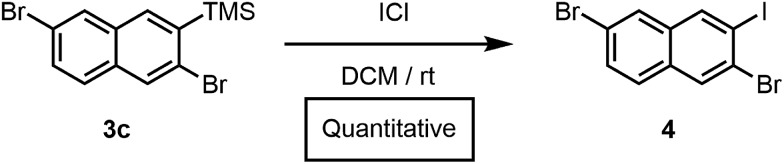
Conversion of **3c** to **4**.

The 2-silyl-3-iodonaphthalenes serve as efficient precursors of 2-naphthyne intermediates in the presence of F^–^ at room temperature, as demonstrated by furan trapping experiments.^20–22^ Unsubstituted (**3a**), 6-bromo- (**3b**), 6,7-dichloro- (**3h**), and 6,7-dibutyl-substituted (**3j**) 2-naphthynes provided [2.2.1]oxabicyclic alkenes (**5a–d**) in good to excellent isolated yields (68–86%, Scheme 3). These structures are of interest as precursors of poly(*ortho*-phenylene)s, iptycenes, acenes, and other aromatic structures.^23–31^ Given the availability of many substituted benzaldehyde cycloaddition partners, these findings demonstrate that benzannulation chemistry provides rapid entry to many substituted 2-naphthyne intermediates.

**Scheme 3:**
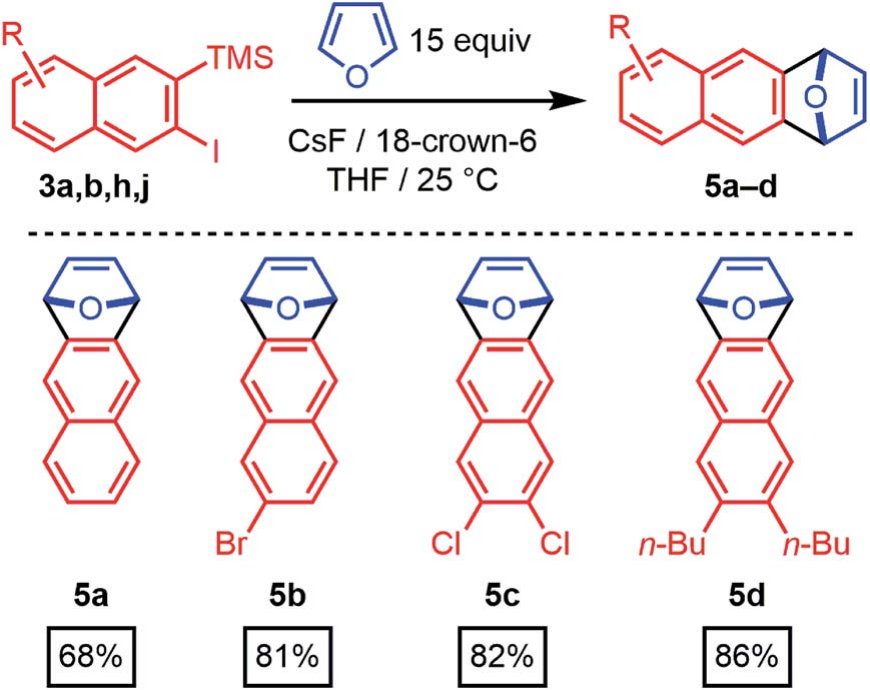
Aryne generation and trapping with furan.

The synthesis of oligo- and poly(*o*-arylene)s represents a long-standing synthetic challenge. Oligomers have been prepared through stepwise cross-coupling strategies^32,33^ or cycloaddition approaches^34^ and adopt preferred or exclusive helical conformations. Formal or direct aryne polymerizations have provided the first polymers with moderate to high degrees of polymerization (*D*_P_: 20–100).^23,35^ We explored the CuCN-mediated polymerization of **3j** (Fig. 4a) under conditions adapted from Uchiyama's pioneering study.^35^ Naphthalene derivative **3j** (0.235 mM) was polymerized in THF at 25 °C in the presence of CsF (2 equiv.), CuCN (0.05 equiv.). *n*-BuLi (0.10 equiv.) and 18-crown-6 (4 equiv.). The addition of *n*-BuLi to CuCN generates a Lipshultz-type cuprate that is thought to be the active catalytic species in this reaction.^35,36^ These conditions provided **6** as an oligomeric mixture in modest isolated yield (19%). Size-exclusion chromatography (SEC) of **6** (Fig. 3) indicated a monomodal molecular weight distribution with *M*_n_ = 1900 g mol^–1^ compared to polystyrene standards, with tailing to low molecular weight, corresponding to desilylated and/or dehalogenated naphthalene side products. MALDI-TOF mass spectrometry (see ESI†) showed peaks with spacings consistent with the expected dibutylnaphthalene repeat unit and suggested that these species had well-defined nitrile end group on one end of the oligomer chain. These results confirm the expected reactivity of **3j** under reported aryne polymerization conditions, despite the formation of low molecular weight species. It may ultimately prove possible to achieve higher yields and *D*_P_ by optimizing the rate of 2-naphthyne generation. However, our efforts to explore the role of reaction conditions, such as increased catalyst loading and temperatures, provided unexpected and previously undescribed cyclooligomerization products in synthetically useful yields (Fig. 4a).

**Fig. 4:**
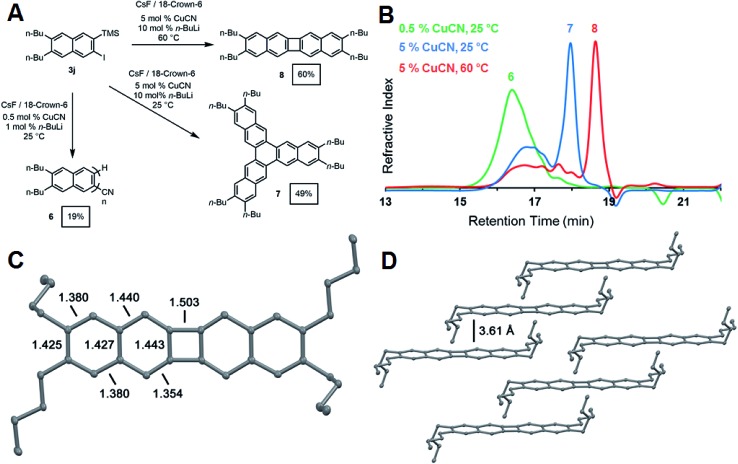
(A) Synthesis of oligo(*ortho*-naphthalene) **6** and cyclic compounds **7** and **8**. All yields are isolated yields. Reaction conditions: **3j** (0.1 mM in THF), CsF (2 equiv.), and 18-crown-6 (4 equiv.). (B) SEC traces for the copper mediated oligomerization and cyclization of **3j** with 0.5 mol% CuCN at room temperature (green), 5 mol% CuCN at room temperature (blue), and 5 mol% CuCN at 60 °C (red). Molecular weight distributions were determined by calibration with polystyrene standards. (C) Single crystal X-ray structure of **8** showing bond C–C distances around the aromatic rings in Å. Hydrogens are omitted for clarity, thermal ellipsoids shown at the 50% probability level. (D) Solid state packing arrangement of **8** illustrating the interplanar distances between arenes.

During our attempts to obtain higher molecular weight polymers, we found the product distribution drastically changed when the CuCN loading was increased to 5 mol% and the reaction run at room temperature. Analysis of the GPC trace of the precipitated product revealed a significant shift in the retention time of the oligomeric peak reflective of shorter oligomers (Fig. 4b). A sharp peak emerged at significantly longer retention times, suggesting a well-defined, low molecular weight compound. The ^1^H and ^13^C NMR spectra indicated the formation of a single naphthyl species with only two aromatic ^1^H resonances and five aromatic carbon shifts, suggesting the formation of a cyclic compound with a high degree of symmetry (see ESI Fig. S40 and S41†). Finally, high-resolution mass spectrometry of the isolated product after purification by chromatography identified it as the cyclic hexabutyltrinaphthylene **7**, which was isolated in 49% yield (Fig. 4a). This extended form of triphenylene is of interest for discotic liquid crystals and as a potential monomer for covalent organic frameworks (COFs).^37^

When the above reaction is run at elevated temperature, a different molecular product dominates, as indicated by a shift to longer retention time in the GPC trace of the crude reaction mixture (Fig. 4b). We identified this species as the tetrabutylbinaphthalene **8** through ^1^H and ^13^C NMR (ESI Fig. S42 and S43†) spectroscopy and high-resolution mass spectrometry. Single-crystal X-ray crystallography unambiguously identified the structure as tetrabutylbinaphthalene product **8** (Fig. 4c). The solid-state arrangement of **8** features cofacial π-stacking with an unusually long interplanar distance of 3.61 Å, compared to the typical 3.4 Å distance for acenes (Fig. 4d).^38^ This arrangement arises from the butyl side chains, whose conformation places their termini perpendicular to the arene plane, preventing closer cofacial packing. [*N*]Phenylenes are traditionally challenging synthetic targets. Vollhardt and coworkers have prepared many of these compounds through a cobalt-catalyzed [2 + 2 + 2] cycloaddition of phenylene ethynylenes,^39^ and new synthetic strategies have been reported by Swager,^40^ Xia,^41^ and Bunz.^42^ These compounds consist of alternating aromatic rings fused cyclobutadienes with strong anti-aromatic character. The X-ray crystal structure showed a nearly planar geometry with torsion angles of 1.56° and 0.55° around the phenylene link. Analysis of the bond lengths confirms localization of the π-bonds around the 4-membered ring to offset the antiaromatic character of the cyclobutadienoid with alternating 1.44 Å endocyclic and 1.35 Å exocyclic bond distances. Bonds linking the acenoid segments have a larger single bond character with a bond length of 1.50 Å (Fig. 4c).^43^ UV-vis absorption and photoemission spectra of phenylenes **7** and **8** also show distinct differences to naphthyne precursor **3j** and oligo(*ortho*-naphthalene) **6** (see ESI†).

## Conclusions

The benzannulation of halosilylalkynes provides a general and rapid method to produce substituted 2-halo-3-silylnaphthalenes with high regioselectivity. The addition of a fluoride anion generates 2-naphthyne reactive intermediates, which were trapped as the [2.2.1]oxabicyclic alkene. These trapped arynes are themselves synthetically useful building blocks.^23–31^ A copper mediated aryne polymerization afforded low molecular weight oligomers, however when higher catalyst loadings are used the [2 + 2 + 2] and [2 + 2] cycloaddition products were observed in synthetically useful yields. These results show the utility of sequential zinc-catalyzed benzannulation reaction and naphthyne generation to access substituted tri- and binaphthalenes that are otherwise not easily prepared. We anticipate that the control of the halogenation pattern in conjunction with the diversity of available aryne reactions will enable rapid access to diverse and unique polycyclic conjugated aromatic architectures, including functionalized acenes and poly(arylene)s, among others.
