# Supplementary material for: Rapid access to substituted 2-naphthyne intermediates via the benzannulation of halogenated silylalkynes
Source: Chem Sci. 2017 Jun 9;8(8):5675–81. doi: 10.1039/c7sc01625e (PMC5621055; doi:10.1039/c7sc01625e)
Supplement: Supplementary file 1 [file SC-008-C7SC01625E-s001.pdf]

Electronic Supplementary Material (ESI) for Chemical Science.
This journal is © The Royal Society of Chemistry 2017

# Rapid Access to Substituted 2-Naphthyne Intermediates via the Benzannulation of Halogenated Silylalkynes

SAMUEL J. HEIN[a],[b], DAN LEHNHERR[b], AND WILLIAM R. DICHTEL[a],*

[a]*Department of Chemistry, Northwestern University, Evanston, Illinois, 60208 USA*

[b]*Department of Chemistry and Chemical Biology, Cornell University, Baker Laboratory, Ithaca, New York, 14853 USA*

**Supplementary Information**

**Table of Contents**

A. Materials and Instrumentation S2

B. Synthetic Procedures S3

C. 1D NMR Spectroscopy S16

D. 2D NMR Spectroscopy S43

E. DFT Calculations S54

F. MALDI-TOF-MS S73

G. Single Crystal X-Ray Data S74

H. UV/Vis and Fluorescence Spectroscopy S84

I. References to Supporting Information S85

A. **Materials.** Benzaldehydes **1a**, **1b**, **1c**, **1d**, **1e**, **1f,** and **1i** were all synthesized according to a literature procedure.[1] 18-Crown-6 which was recrystallized from anhydrous acetonitrile and dried under vacuum. $ZnCl_2$ (1 M in $Et_2O$) was purchased from Sigma Aldrich and used as is without further purification. Tetrahydrofuran was purchased from commercial sources and purified using a custom-built alumina-column based solvent purification system. Other solvents were purchased from commercial sources and used without further purification.

**Instrumentation.** Infrared spectra were recorded on a Thermo Nicolet iS10 with a zinc selenide ATR attachment and are uncorrected. UV/Vis absorbance spectra were recorded on a Cary 5000 UV-Vis-NIR spectrophotometer with an Hg lamp. Photoemission spectra were recorded on a Horiba Jobin Yvon Fluorolog-3 fluorescence spectrophotometer equipped with a 450 W Xe lamp, double excitation and double emission monochromators, a digital photon-counting photomultiplier and a secondary InGaAs detector for the NIR range.

High resolution mass spectra were recorded on an Agilent 6210A LC/MS with either an electrospray ionization (ESI) or an Agilent 7890 GC with electron impact ionization time-of-flight (EI-TOF) or atmospheric pressure photoionization (APPI) sources. Matrix assisted laser desorption ionization time-of-flight (MALDI-TOF) mass spectra were recorded on a Bruker Autoflex III with a 2,5-dihydroxybenzoic acid matrix and silver trifluoroacetate additive. Gel Permeation Chromatography traces were recorded using 2 PolyPore 300×7.5mm columns with 18 Angles Dawn Heleos Multi Angle Light Scattering and Optilab T-rex Differential Refractive Index detectors. Polystyrene standards (1KDa, 2KDa, 3KDa, 5KDa) were purchased from Agilent and used as is.

NMR spectra of solutions were recorded on a Varian 400 spectrometer with an ASW probe with a 20 MHz sample spin rate, 400 MHz Agilent DD MR-400 NMR equipped with AutoX probe, Bruker 500 MHz spectrometer with a DCH CryoProbe. 2D NMR were recorded on a 400 MHz Agilent DD MR-400 NMR with AutoX probe and a 600 MHz Bruker Avance III spectrometer with BBI probe. All spectra were recorded at ambient temperature.

**B. Synthetic Procedures**

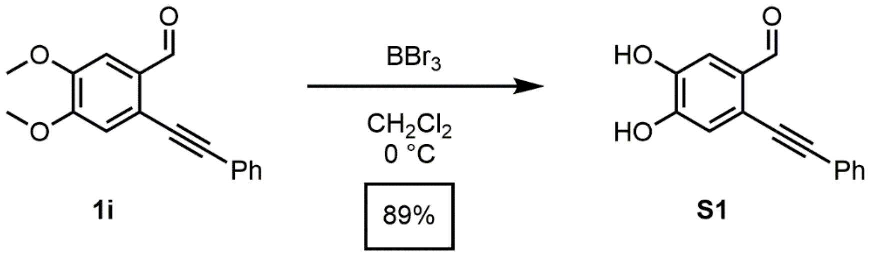


**Synthesis of S1**: **1i** (10.024 g, 37.645 mmol) was transferred to a flame dried round bottom flask and dissolved in anhydrous $CH_2Cl_2$ (180 mL) at 0 °C. Boron tribromide (56 mL, 1 M in $CH_2Cl_2$) was slowly added to the reaction over 5 minutes. The mixture was removed from the ice bath and allowed to warm to rt. After 12 hours, the reaction was again placed in a 0 °C bath and methanol (50 mL) was slowly added to quench any unreacted boron tribromide. The mixture was washed with 2 M HCl (100 mL), extracted with $CH_2Cl_2$ (3 × 100 mL), dried with $MgSO_4$ and filtered. The solvent was concentrated to dryness before being redissolved in methanol (50 mL) and 1 mL $CF_3CO_2H$ and stirred at room temperature for 1 hour. The solvent was removed under vacuum and purified by silica gel column chromatography (Hexanes/Ethyl Acetate, 1:1). **S1** was isolated as a white solid (8.011 g, 89 %). $^1$H NMR (400 MHz, DMSO-$d_6$): δ 10.27 (s, 1H), 7.60 (m, 2H), 7.43 (m, 3H), 7.26 (s, 1H), 7.02 (s, 1H) ppm. $^{13}$C NMR (100 MHz, DMSO-$d_6$): δ 189.4, 151.7, 147.0, 131.4, 128.9, 128.7, 128.7, 122.1, 119.0, 118.4, 113.2, 93.2, 85.2 ppm. IR (solid, ATR) 3271, 2481, 2393, 1634, 1598, 1562, 1507, 1440, 1424, 1353, 1316, 1261, 1213, 1189, 1165, 1088, 984, 955, 880, 754, 746, 684, 662 cm$^{-1}$. HRMS (APPI) calcd for $C_{15}H_{10}O_3$ ($[M]^+$) 238.0624, found 238.0633.

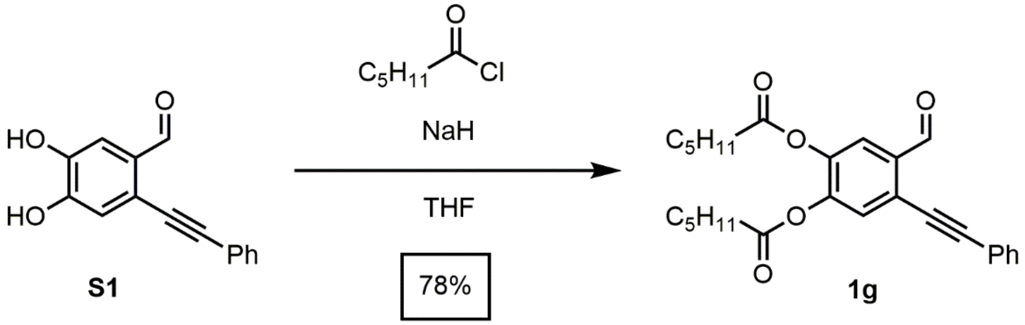


**Synthesis of 1g: S1** (3.007 g, 12.62 mmol) and a 60 % dispersion of NaH in mineral oil (1.08 g, 26.9 mmol) were transferred to a flame dried flask. The reagents were dissolved in anhydrous THF (130 mL) and the reaction was allowed to stir at rt for 1 hour. Hexanoic acid chloride (3.746 g, 27.83 mmol) were then added to the mixture slowly and stirred for 1 hour at rt. The reaction was washed with 2 M HCl (50 mL) and extracted with $CH_2Cl_2$ (3 × 50 mL). The organic layer was dried using $MgSO_4$, filtered, and concentrated before being purified by silica gel column chromatography. Compound **1g** was isolated as a light orange oil (4.261 g, 78 %). $^1$H NMR (400 MHz, $CDCl_3$): δ 10.55 (s, 1H), 7.76 (s, 1H), 7.58-7.53 (m, 2H), 7.51 (s, 1H), 7.42-7.37 (m, 3H), 2.58 (t, $J$ = 7.7 Hz, 4H), 1.80-1.71 (m, 4H), 1.43-1.35 (m, 10H), 0.96-0.94 (t, $J$ = 6.90 Hz, 6 H) ppm. $^{13}$C NMR (100 MHz, $CDCl_3$): δ 189.9, 170.7, 170.4, 146.8, 142.9, 134.3, 131.9, 129.4, 128.7, 128.1, 125.4, 122.6, 122.1, 97.1, 83.6, 34.2, 34.0, 31.4, 31.3, 24.7, 24.6, 22.4, 14.0 ppm. IR (solid, ATR) 2957, 2931, 2871, 1770, 1694, 1603, 1496, 1482, 1467, 1443,

1411, 1379, 1319, 1237, 1130, 1085, 915, 852, 756, 689 cm$^{-1}$. HRMS (ESI) calcd for $C_{27}H_{30}O_5$ ($[M + H]^+$) 435.2166, found 435.2166.

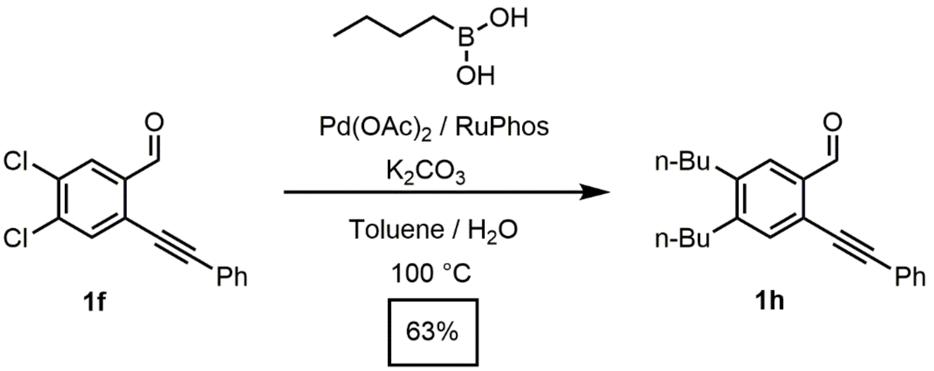


**1f** **1h**

**Synthesis of 1h:** Palladium(II) acetate (0.020 g, 0.089 mmol), RuPhos (0.074 g, 0.160 mmol), potassium carbonate (1.548 g, 11.20 mmol), *n*-butyl boronic acid (0.939 g, 9.21 mmol), and **1f** (1.003 g, 3.645 mmol) were added to a 250 mL round bottom flask before being evacuated and back filled with $N_2$. In a separate flask, 36 mL of a 10:1 (v/v) mixture of toluene and water was sparged with nitrogen gas for one hour before being transferred to the reaction mixture. When all the solvent had been added, the reaction flask was placed in a 100 °C oil bath and allowed to stir at reflux for 6 hours. After the reaction was finished, it was cooled to rt, quenched with 2 M HCl (20 mL), and extracted with $CH_2Cl_2$ (3 × 50 mL). The organic fractions were collected and dried using $MgSO_4$ before being purified by column chromatography (70% Hexanes : 30% $CH_2Cl_2$) to give **1h** as an orange solid (0.730 g, 63 %). $^1$H NMR (500 MHz, $CDCl_3$): δ 10.57 (s, 1H), 7.74 (s, 1H), 7.56 (m, 2H), 7.43 (s, 1H), 7.37 (m, 3H) 2.66 (m, 4H), 1.64-1.56 (m, 4H), 1.47-1.38 (m, 4H), 1.00-0.95 (q, $J$ = 7.3 Hz, 6H) ppm. $^{13}$C NMR (125 MHz, $CDCl_3$): δ 191.9, 147.9, 142.2, 133.9, 131.8, 128.9, 128.6, 127.9, 124.4, 122.8, 95.2, 85.5, 33.1, 33.0, 32.7, 32.4, 22.9, 22.9, 14.1 ppm. IR (solid, ATR) 2955, 2927, 2870, 1684, 1601, 1494, 1464, 1441, 1430, 1393, 1378, 1315, 1260, 1206, 1171, 1152, 1103, 1067, 1025, 937, 913, 879, 758, 733, 690, 667, 654 cm$^{-1}$. HRMS (ESI) calcd for $C_{23}H_{26}O$ ($[M + H]^+$) 319.2056, found 319.2056.

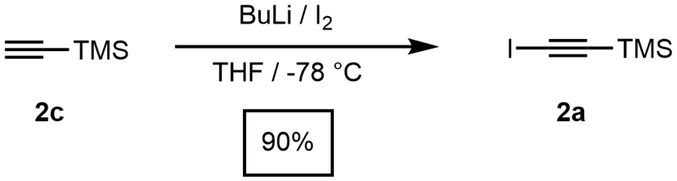


**2c** **2a**

**Synthesis of 2a:** To a flame dried flask, **2c** (2.9 g, 4.3 mL, 30 mmol) was dissolved in THF (50 mL) and placed in a -78 °C dry ice/acetone bath. To this solution, *n*-butyl lithium (24.5 mL, 2.5 M in THF) was added dropwise and allowed to stir for 15 minutes before a solution of $I_2$ (8.625 g, 33.98 mmol) in THF (50 mL) was added dropwise. The reaction was stirred for another 15 minutes at -78 °C before washing with saturated aqueous $Na_2S_2O_3$ (50 mL). The reaction mixture was extracted with ethyl ether (3×50 mL), before being dried with sodium sulfate and filtered. The solvent was removed under reduced pressure to give compound **2a** (6.384 g, 90 % yield). $^1$H and $^{13}$C NMR data are consistent with literature values.[2]

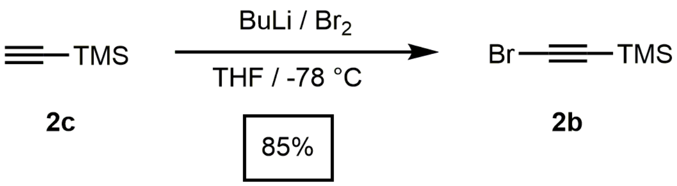

**Synthesis of 2b:** To a flame dried flask, **2c** (4.992 g, 50.94 mmol) was dissolved in THF (50 mL) and placed in a -78 °C dry ice/acetone bath. To this solution, *n*-butyl lithium (24.5 mL, 2.5 M in THF) was added dropwise and allowed to stir for 15 minutes before $Br_2$ (11.443 g, 71.60 mmol) was added dropwise. The reaction was let to stir for another 15 minutes at -78 °C before washing with saturated aqueous $Na_2S_2O_3$ (50 mL). The reaction mixture was extracted with ethyl ether (3×50 mL), before being dried with sodium sulfate and filtered. The solvent was removed under reduced pressure to give compound **2b** (7.719 g, 85 % yield). $^1H$ and $^{13}C$ NMR data were consistent with literature values.[3]

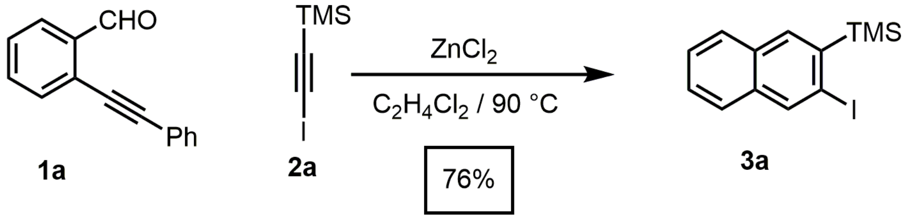

**Synthesis of 3a: 2a** (0.503 g, 2.24 mmol) and **1a** (0.946 g, 4.59 mmol) were dissolved in 1,2-DCE (5 mL). To this solution, 0.1 mL of $ZnCl_2$ (0.2 eq, 1 M in ethyl ether) was added via syringe. The reaction mixture turned a light yellow color and the reaction was placed in a 90 °C oil bath for 12 hours. The reaction was then poured into hexanes and passed through a silica plug using hexanes as the eluent. The solvent was removed under reduced pressure to yield compound **3a** as a yellow liquid (0.558 g, 76 % yield). $^1H$ NMR (300 MHz, $CDCl_3$): δ 8.39 (s, 1H), 7.88 (s, 1H), 7.79 (m, 1H), 7.68 (m, 1H), 7.49 (m, 2H), 0.52 (s, 9H) ppm. $^{13}C$ NMR (100 MHz, $CDCl_3$): δ 142.2, 138.6, 136.7, 135.2, 131.8, 128.2, 127.2, 126.6, 126.4, 99.7, 0.1 ppm. IR (solid, ATR) 3050, 2951, 2891, 1620, 1567, 1482, 1405, 1288, 1246, 1196, 1137, 1091, 1017, 950, 871, 831, 742, 688 $cm^{-1}$. HRMS (EI-TOF) calcd for $C_{13}H_{15}ISi$ ($[M]^+$) 325.9988, found 325.9981.

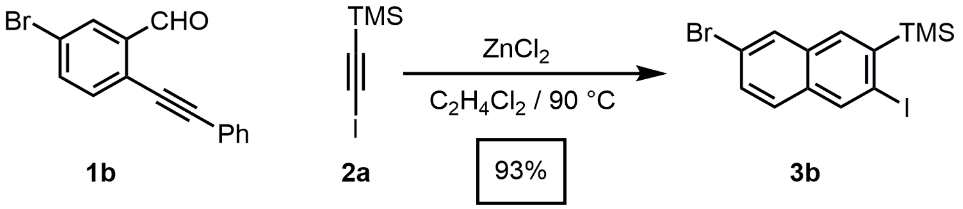

**Synthesis of 3b: 2a** (0.104 g, 0.464 mmol) and **1b** (0.181 g, 0.635 mmol) were dissolved in 1,2-DCE (4.5 mL). To this solution, 0.09 mL of $ZnCl_2$ (0.2 eq, 1 M in ethyl ether) was added via syringe. The reaction mixture turned a light yellow color and the reaction was placed in an 90 °C oil bath for 12 hours. The reaction was then poured into hexanes and passed through a silica plug using hexanes as the eluent. The solvent was removed under reduced pressure to yield compound **3b** as a clear oil (0.169 g, 93 % yield). $^1H$ NMR (600 MHz, $CDCl_3$): δ 8.30 (s, 1H), 7.92 (s, 1H), 7.74 (s, 1H), 7.51 (dd, $J$ = 1.9 Hz, $J$ = 8.7 Hz, 1H), 7.48 (d, $J$ = 8.7 Hz, 1H), 0.51 (s, 9H) ppm. $^{13}C$ NMR (150 MHz, $CDCl_3$): δ 143.6, 138.2, 135.4, 133.3, 130.4, 130.1, 127.9, 120.2, 99.8, -0.1 ppm. IR (solid, ATR) 2950, 1562, 1550, 1473, 1407, 1376, 1247, 1175, 1141, 1094, 1062,952,

887, 834, 798, 750, 689 cm$^{-1}$. HRMS (EI-TOF) calcd for $C_{13}H_{14}SiBrI$ ($[M]^+$) 403.9093, found 403.9116.

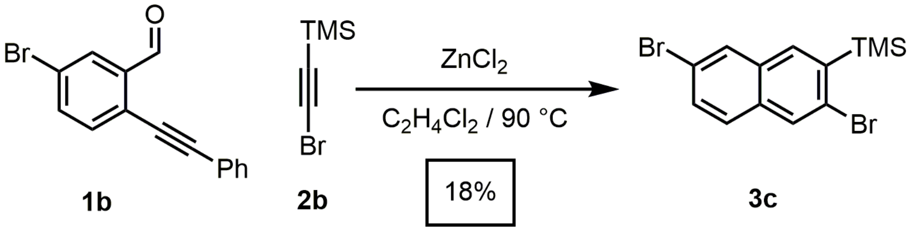


**1b** **2b** **3c**

**Synthesis of 3c: 2b** (0.255 g, 1.44 mmol) and **1b** (0.565 g, 1.98 mmol) are dissolved in 1,2-DCE (14 mL). To this solution, 0.3 mL of $ZnCl_2$ (0.2 eq, 1 M in ethyl ether) was added via syringe. The reaction mixture turned a light yellow color and the reaction was placed in an 80 °C oil bath for 12 hours. The reaction was then poured into hexanes and passed through a silica plug using hexanes as the eluent plug and eluted with hexanes. The solvent was removed under reduced pressure to yield compound **3c** a white solid (0.091 g, 18 % yield). $^1$H NMR (400 MHz, $CDCl_3$): 7.97 (s, 1H), 7.96 (s, 1H), 7.80 (s, 1H), 7.55 (s, 2H), 0.48 (s, 9H) ppm. $^{13}$C NMR (100 MHz, $CDCl_3$): δ 140.5, 135.7, 135.7, 133.2, 132.5, 130.7, 130.2, 128.2, 127.3, 120.0, -0.2. IR (solid, ATR) 2953, 2896, 1567, 1551, 1472, 1409, 1378, 1336, 1282, 1248, 1242, 1174, 1142, 1102, 1062, 959, 907, 893, 879, 834, 800, 759, 750, 689 cm$^{-1}$. HRMS (EI-TOF) calcd for $C_{13}H_{14}SiBr_2$ ($[M]^+$) 355.9232, found 355.9247.

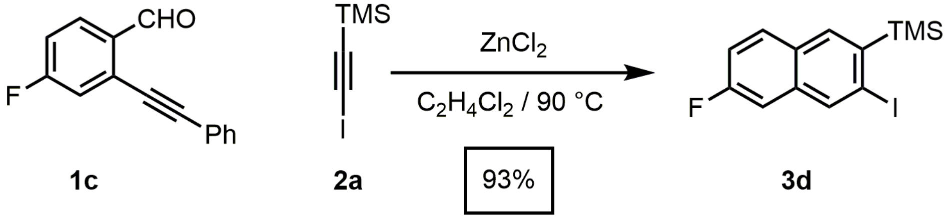


**1c** **2a** **3d**

**Synthesis of 3d: 2a** (0.105 g, 0.471 mmol) and **1c** (0.146 g, 0.651 mmol) are dissolved in 1,2-DCE (4.5 mL). To this solution, 0.09 mL of $ZnCl_2$ (0.2 eq, 1 M in ethyl ether) was added via syringe. The reaction mixture turned a light yellow color and the reaction was placed in a 90 °C oil bath for 12 hours. The reaction was then poured into hexanes and passed through a silica plug using hexanes as the eluent. The solvent was removed under reduced pressure to yield compound **3d** as a light yellow solid (0.144 g, 93 % yield). $^1$H NMR (400 MHz, $CDCl_3$): δ 8.32 (s, 1 H), 7.86 (s, 1H), 7.77 (dd, $J$ = 5.3 Hz, 8.5 Hz, 1H), 7.29-7.23 (m, 2H), 0.54 (s, 9H) ppm. $^{13}$C NMR (100 MHz, $CDCl_3$): δ 161.1 ($J$ = 248.8 Hz), 141.5 ($J$ = 2.6 Hz), 137.9 ($J$ = 5.3 Hz), 136.4 ($J$ = 1.2 Hz), 135.9 ($J$ = 9.8 Hz), 130.8 ($J$ = 9.3 Hz), 128.9 ($J$ = 0.9 Hz), 117.0 ($J$ = 25.3 Hz), 109.5 ($J$ = 20.93 Hz), 101.2, 0.02 ppm. $^{19}$F NMR (375 MHz, $CDCl_3$) δ -112.01 (td, $J$ = 6.1 Hz, 9.0 Hz, 1F) ppm IR (solid, ATR) 2951, 1627, 1563, 1484, 1433, 1379, 1332, 1276, 1246, 1229, 1204, 1146, 1122, 1091, 970, 950, 931, 893, 881, 836, 801, 757, 746, 689 cm$^{-1}$. HRMS (EI-TOF) calcd for $C_{13}H_{14}SiFI$ ($[M]^+$) 343.9894, found 343.9897.

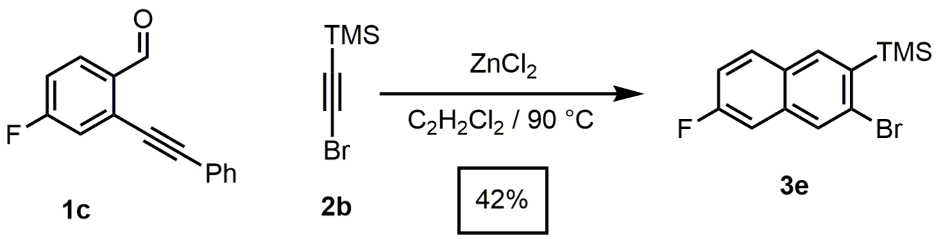

**Synthesis of 3e: 2a** (0.247 g, 1.39 mmol) and **1c** (0.465 g, 1.56 mmol) were dissolved in 1,2-DCE (14 mL). To this solution, 0.2 mL of $ZnCl_2$ (0.2 eq, 1 M in ethyl ether) was added via syringe. The reaction mixture turned a light yellow color and the reaction was placed in a 90 °C oil bath for 12 hours. The reaction was then poured into hexanes and passed through a silica plug using hexanes as the eluent plug and eluted with hexanes. The solvent was removed under reduced pressure to yield compound **3e** as a pale yellow oil (0.173 g, 42 % yield). $^1$H NMR (400 MHz, $CDCl_3$): δ 7.93 (s, 1H), 7.86 (s, 1H), 7.75 (dd, *J* = 5.7 Hz, *J* = 8.7 Hz, 1H), 7.27 (dd, *J* = 2.3 Hz, *J* = 9.6 Hz, 1H), 7.21 (dt, *J* = 2.3 Hz, 8.7 Hz, 1H), 0.46 (s, 9H) ppm. $^{13}$C NMR (100 MHz, $CDCl_3$): δ 161.4 (*J* = 248.6 Hz), 138.3 (*J* = 2.8 Hz), 136.6, 135.7 (*J* = 10.3 Hz), 130.7 (*J* = 9.3 Hz), 130.1 (*J* = 5.7 Hz), 128.7, 128.3f, 116.7 (*J* = 25.6 Hz), 109.8 (*J* = 21.2 Hz), -0.15 ppm. $^{19}$F NMR (375 MHz, $CDCl_3$) δ -112.3 (dt, *J* = 5.6 Hz, 8.9 Hz, 1F) ppm. IR (solid, ATR) 2954, 1627, 1564, 1486, 1438, 1381, 1336, 1280, 1247, 1231, 1205, 1145, 1123, 1096, 972, 947, 882, 836, 804, 755, 690 $cm^{-1}$. HRMS (EI-TOF) calcd for $C_{13}H_{14}FSiBr$ ($[M]^+$) 296.0032, found 296.0039.

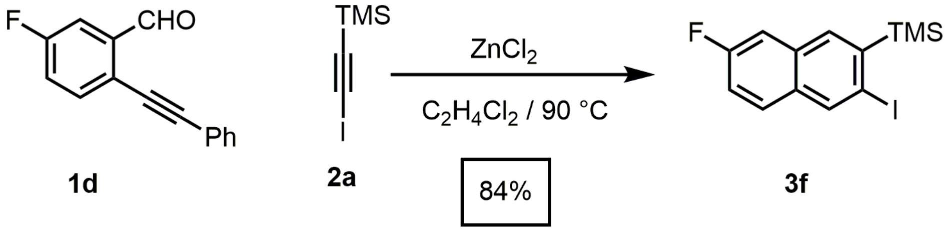

**Synthesis of 3f: 2a** (0.103 g, 0.461 mmol) and **1d** (0.144 g, 0.642 mmol) were dissolved in 1,2-DCE (4.5 mL). To this solution, 0.09 mL of $ZnCl_2$ (0.2 eq, 1 M in ethyl ether) was added via syringe. The reaction mixture turned a light yellow color and the reaction was placed in a 90 °C oil bath for 12 hours. The reaction was then poured into hexanes and passed through a silica plug using hexanes as the eluent. The solvent was removed under reduced pressure to yield compound **3f** as a pale yellow oil (0.131 g, 84 % yield). $^1$H NMR (400 MHz, $CDCl_3$): δ 8.36 (s, 1H), 7.82 (s, 1H), 7.65 (dd, *J* = 5.6 Hz, *J* = 9.0 Hz, 1H), 7.40 (dd, *J* = 2.2 Hz, *J* = 9.7 Hz, 1H), 7.26 (td, *J* = 2.2 Hz, *J* = 9.0 Hz, 1 H) 0.53 (s, 9H) ppm. $^{13}$C NMR (100 MHz, $CDCl_3$): δ 160.8 (*J* = 247.0 Hz), 143.5 (*J* = 0.8 Hz), 138.4 (*J* = 1.1 Hz), 135.9 (*J* = 5.5 Hz), 132.5 (*J* = 9.0 Hz), 132.2 (*J* = 0.9 Hz), 128.9 (*J* = 9.1 Hz), 117.7 (*J* = 25.6 Hz), 111.3 (*J* = 20.5 Hz), 98.4 (*J* = 3.0 Hz), 0.0 ppm. $^{19}$F NMR (375 MHz, CDCl3) δ -113.0 ppm (td, *J* = 5.4 Hz, *J* = 9.1 Hz, 1F). IR (solid, ATR) 2952, 1628, 1570, 1489, 1387, 1335, 1277, 1246, 1197, 1144, 1122, 1093, 937, 898, 878, 835, 803, 759, 739, 688 $cm^{-1}$. HRMS (EI-TOF) calcd for $C_{13}H_{14}SiFI$ ($[M]^+$) 343.9894, found 343.9879.

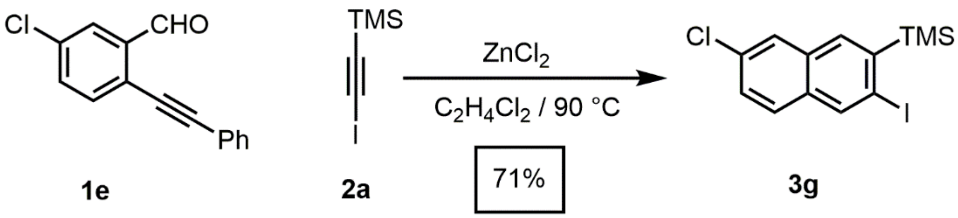


**Synthesis of 3g: 2a** (0.102 g, 0.450 mmol) and **1e** (0.154 g, 0.640 mmol) were dissolved in 1,2-DCE (4.5 mL). To this solution, 0.09 mL of $ZnCl_2$ (0.2 eq, 1 M in ethyl ether) was added via syringe. The reaction mixture turned a light yellow color and the reaction was placed in a 90 °C oil bath for 12 hours. The reaction was then poured into hexanes and passed through a silica plug using hexanes as the eluent. The solvent was removed under reduced pressure to yield compound **3g** as a clear oil (0.115 g, 71 % yield). $^1$H NMR (300 MHz, $CDCl_3$): δ 8.34 (s, 1H), 7.78 (s, 2H), 7.59 (d, *J* = 8.9 Hz, 1H), 7.55 (dt, *J* = 1.5, *J* = 8.7, 1H), 0.53 (s, 9H) ppm. $^{13}$C NMR (75 MHz, $CDCl_3$): δ 143.7, 138.3, 135.6, 133.2, 132.3, 132.1, 128.1, 127.9, 126.9, 99.8, -0.0 ppm. IR (solid, ATR) 2951, 1566, 1553, 1474, 1409, 1382, 1246, 1175, 1139, 1097, 1080, 953, 953, 900, 879, 833, 800, 751, 733, 688, 667 cm$^{-1}$. HRMS (EI-TOF) calcd for $C_{13}H_{14}SiClI$ ($[M]^+$) 359.9598, found 359.9569.

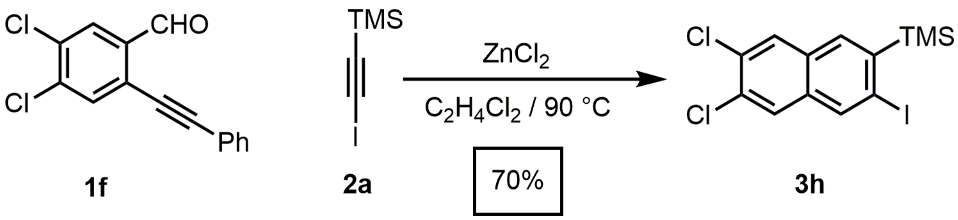


**Synthesis of 3h: 2a** (0.307 g, 1.37 mmol) and **1f** (0.507 g, 1.84 mmol) were dissolved in 1,2-DCE (13.5 mL). To this solution, 0.54 eq of $ZnCl_2$ (0.4 eq, 1 M in ethyl ether) was added via syringe. The reaction mixture turned a light yellow color and the reaction was placed in a 90 °C oil bath for 12 hours. The reaction was then poured into hexanes and passed through a silica plug using hexanes as the eluent. The solvent was removed under reduced pressure to yield compound **3h** as a white solid (0.370 g, 70 % yield). $^1$H NMR (500 MHz, $CDCl_3$): δ 8.28 (s, 1H), 7.90 (s, 1H), 7.78 (s, 1H), 7.75 (s, 1H), 0.49 (s, 9H) ppm. $^{13}$C NMR (125 MHz, $CDCl_3$): 144.3, 137.4, 135.3, 133.8, 131.6, 130.8, 130.7, 129.2, 127.3, 100.9, -0.07 ppm. IR (solid, ATR) 2954, 1548, 1460, 1407, 1352, 1243, 1175, 1118, 1093, 981, 972, 931, 903, 885, 825, 752, 679 cm$^{-1}$. HRMS (EI-TOF) calcd for $C_{13}H_{13}SiCl_2$ ($[M]^+$) 393.9208, found 393.9211.

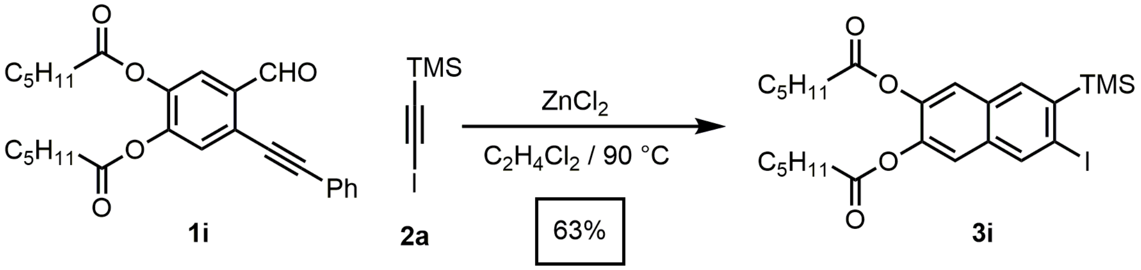


**Synthesis of 3i: 2a** (0.105 g, 0.470 mmol) and **1i** (0.263 g, 0.605 mmol) were dissolved in 1,2-DCE (4.5 mL). To this solution, 0.09 mL of $ZnCl_2$ (0.2 eq, 1 M in ethyl ether) was added via syringe. This reaction flask was then placed into a 90 °C oil bath and allowed to stir for 2 hours. When the reaction was complete, the solvent was removed under reduced pressure and product

was purified by column chromatography (1:1 Hexanes/$CH_2Cl_2$). Compound **3i** was isolated as a colorless oil (0.1557 g, 63 %). $^1$H NMR (500 MHz, $CDCl_3$): 8.33, (s, 1H), 7.81 (s, 1H), 7.61 (s, 1H) 7.48 (s, 1H), 2.57 (dt, $J$ = 2.5, 7.6 Hz, 4H), 1.81-1.74 (m, 4H), 1.46-1.36 (m, 8H), 0.94 (t, $J$ = 6.9 Hz, 6H), 0.48, (s, 9H) ppm. $^{13}$C NMR (125 MHz, $CDCl_3$): δ 171.3, 171.3, 143.0, 142.2, 141.7, 137.9, 136.1, 133.2, 130.0, 121.4, 119.5, 100.0, 34.3, 31.5, 24.8, 22.5, 14.1, -0.03 ppm. IR (solid, ATR) 2955, 2929, 2859, 1766, 1629, 1667, 1488, 1466, 1425, 1377, 1340, 1246, 1226, 1159, 1137, 1095, 949, 917, 837, 760, 689 cm$^{-1}$. HRMS (ESI) calcd for $C_{25}H_{35}O_4ISiNa$ ([M + Na]$^+$) 577.1242, found 577.1243.

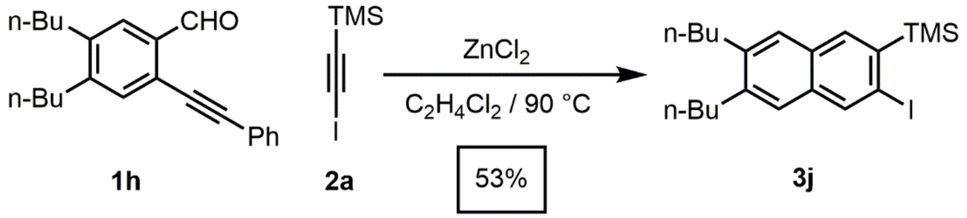


**Synthesis of 3j: 2a** (0.300 g, 1.34 mmol) and **1h** (0.568 g, 1.78 mmol) were dissolved in 1,2-DCE (30 mL). To this solution, 0.3 mL of $ZnCl_2$ (0.2 eq, 1 M in ethyl ether) were added and the reaction was placed into a 90 °C oil bath and allowed to stir for 2 hours. When the reaction was complete, the solvent was removed under reduced pressure and product was purified by column chromatography with hexanes as the eluent. Compound **3j** was isolated as a colorless oil (0.347 g, 59%). $^1$H NMR (500 MHz, $CDCl_3$): δ 8.28 (s, 1H), 7.78 (s, 1H), 7.54 (s, 1H), 7.43 (s, 1H), 2.78-2.73 (m, 4H), 1.71-1.60 (m, 4H), 1.50-1.37 (m, 4H), 1.00-0.95 (dt, $J$ = 2.8, 7.2 Hz, 6H) ppm. $^{13}$C NMR (125 MHz, $CDCl_3$): δ 141.3, 140.9, 140.6, 137.8, 136.0, 134.2, 130.6, 127.2, 125.5, 98.5, 33.3, 33.3, 32.7, 23.0, 22.9, 14.2, 14.2, 0.1 ppm. IR (solid, ATR) 2954, 2928, 2869, 2857, 1570, 1464, 1376, 1247, 1098, 949, 909, 835, 758, 687 cm$^{-1}$. HRMS (EI-TOF) calcd for $C_{21}H_{31}SiI$ ([M]$^+$) 438.1240, found 438.1250.

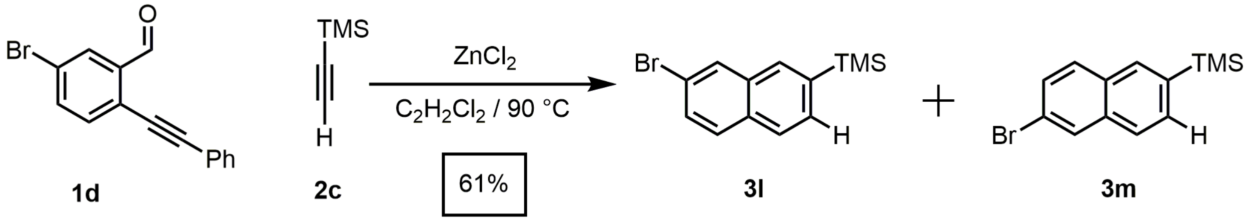


**Synthesis of 3l and 3m: 2c** (0.109 g, 1.11 mmol) and **1d** (0.386 g, 1.35 mmol) were dissolved in 1,2-DCE (10 mL). To this solution, 0.2 mL of $ZnCl_2$ (0.2 eq, 1 M in ethyl ether) was added via syringe. The reaction mixture turned a light yellow color and it was placed in a 90 °C oil bath for 12 hours. After cooling to rt, the solvent was removed under vacuum and the product was purified by silica gel chromatography using hexanes as the eluent. The products were isolated as a 1:1 inseparable mixture to give **3l** and **3m** as a white solid (0.172 g, 61 % yield). $^1$H NMR of **3l** (400 MHz, $CDCl_3$): δ 8.03 (d, $J$ = 1.6 Hz, 1H), 7.92 (s, 1H), 7.79 (d, $J$ = 8.2 Hz, 1H), 7.69 (d, $J$ =8.4 Hz, 1H), 7.62 (dd, $J$ = 8.2, 1.1 Hz, 1H), 7.55 (d, $J$ = 8.4 Hz, 1H) ppm. $^{13}$C NMR of **3l** (100 MHz, $CDCl_3$): δ 139.6, 143.1, 132.9, 132.1, 130.4, 130.2, 129.7, 129.5, 126.9, 119.8 ppm. $^1$H NMR of **3m** (400 MHz, $CDCl_3$): δ 7.99 (d, $J$ = 1.7 1H), 7.98 (s, 1H), 7.73 (d, $J$ = 8.0 Hz, 1H), 7.72 (d, $J$ = 9.4 Hz, 1H), 7.63 (dd, $J$ = 8.0, 0.9 Hz, 1H), 7.55 (dd, $J$ = 9.0, 1.7 Hz, 1H) ppm, $^{13}$C NMR of **3m** (100 MHz, $CDCl_3$): δ 138.8, 134.8, 133.7, 131.4, 131.4, 131.0, 129.8, 129.4, 126.1, 120.3 ppm, IR (solid, ATR) 3042, 2956, 2894, 1616, 1576, 1493, 1450, 1407, 1310, 1247, 1180, 1139, 1087,

1059, 949, 882, 829, 751, 694, 652 cm$^{-1}$. HRMS (EI-TOF) calcd for $C_{13}H_{15}SiBr$ ($[M]^+$) 278.0126, found 278.0139.

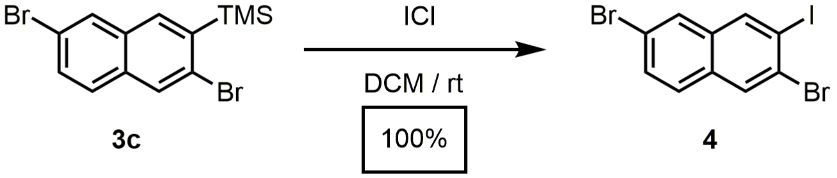


3c 4

**Synthesis of 4: 3c** (0.050 g, 0.140 mmol) and ICl (0.025 g, 0.140 mmol) were dissolved in separate $CH_2Cl_2$ solutions (1 mL and 0.5 mL respectively), which were combined. The resulting reaction mixture was stirred for 5 min, after which the solution was washed with saturated $Na_2S_2O_3$ (1 mL) and extracted with $CH_2Cl_2$ (3 × 3 mL). The organic fractions were combined, dried ($MgSO_4$), and filtered to provide a light yellow solution. The solvent was removed, and **4** was purified using column chromatography ($SiO_2$, hexanes) to provide a white solid in quantitative yield (0.057 g, 100 %). **4:** $^1$H NMR (400 MHz, $CDCl_3$): δ 8.29 (s, 1H), 8.08 (s, 1H), 7.85 (1, 1H) 7.57 (d, 2H) ppm. $^{13}$C NMR (100 MHz, $CDCl_3$): δ 138.6, 134.1, 132.1, 130.9, 130.9, 128.8, 128.6, 126.9, 121.1, 99.9 ppm. IR (solid, ATR) 2956, 1693, 1609, 1560, 1475, 1413, 1375, 1331, 1284, 1263, 1175, 1142, 1088, 1059, 943, 889, 872, 803, 795 cm$^{-1}$. HRMS (DART) calcd for $C_{10}H_5Br_2I$ ($[M]^+$) 409.7797, found 409.7789.

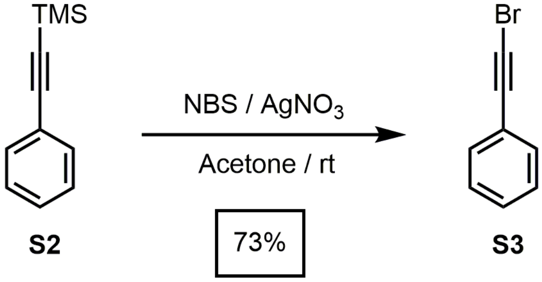


S2 S3

**Synthesis of S3: S2** (0.598 g, 2.87 mmol), *N*-Bromosuccinimide (0.589 g, 3.31 mmol), and $AgNO_3$ (0.499 g, 2.98 mmol) were dissolved in acetone (15 mL) and stirred at rt for 12 h. The solvent was removed under vacuum and the crude reaction mixture was purified by silica gel chromatography using hexanes as the eluent. The solvent was removed under reduced pressure to afford **S3** as a clear liquid (0.3776 g, 73 % yield). $^1$H and $^{13}$C NMR spectral data were consistent with literature values.[4]

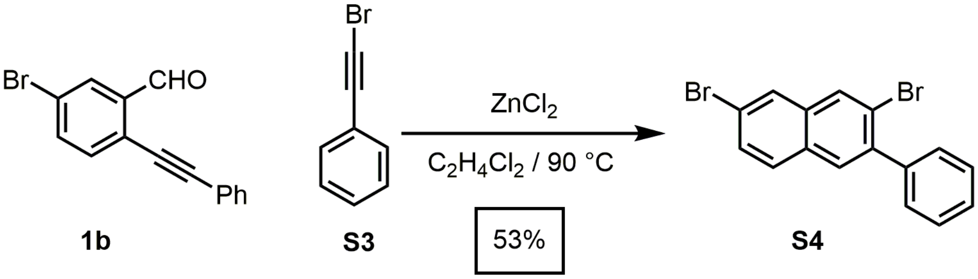


1b S3 S4

**Synthesis of S4:** S3 (0.101 g, 0.553 mmol) and **1b** (0.210 g, 0.720 mmol) were dissolved in 1,2-DCE (3 mL). To this solution, 0.1 mL of $ZnCl_2$ (0.2 eq, 1 M in ethyl ether) was added via syringe. The reaction mixture turned a light yellow color and it was placed in a 90 °C oil bath for 12 hours.

After cooling to rt, the solvent was removed under vacuum and the product was purified by silica gel chromatography using hexanes as the eluent. The product **S4** was isolated as a light yellow solid (0.107 g, 53 % yield). $^{1}$H NMR (400 MHz, $CDCl_3$): δ 8.08 (s, 1H), 7.93 (d, *J* = 2.0 Hz, 1H), 7.75 (s, 1H), 7.66 (d, *J* = 8.75 Hz, 1H), 7.58 (dd, *J* = 2.0 Hz, *J* = 8.75 Hz, 1H), 7.51-7.44 (m, 5H) ppm. $^{13}$C NMR (100 MHz, $CDCl_3$): δ 140.7, 140.7, 134.4, 130.7, 130.7, 130.1, 129.9, 129.7, 129.5, 128.7, 128.1, 127.9, 122.2, 121.0 ppm. IR (solid, ATR) 3056, 1618, 1576, 1557, 1496, 1473, 1442, 1372, 1341, 1302, 1266, 1219, 1170, 1140, 1061, 1027, 952, 836, 802, 760 cm$^{-1}$. HRMS (EI-TOF) calcd for $C_{16}H_{10}Br_2$ ($[M]^+$) 359.9149, found 359.9158.

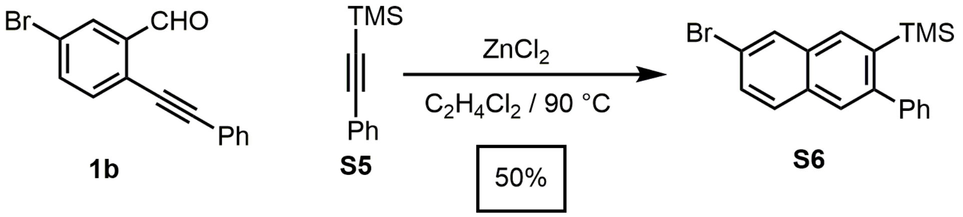


**Synthesis of S6:** S**5** (0.105 g, 0.604 mmol) and **1b** (0.214 g, 0.749 mmol) were dissolved in 1,2-DCE (3 mL). To this solution, 0.11 mL of $ZnCl_2$ (0.2 eq, 1 M in ethyl ether) was added via syringe. The reaction mixture turned a light yellow color and it was placed in a 90 °C oil bath for 12 hours. After cooling to rt, the solvent was removed under vacuum and the product was purified by silica gel chromatography using hexanes as the eluent. The product **S6** was isolated as a light yellow oil (0.108 g, 50 % yield). $^{1}$H NMR (400 MHz, $CDCl_3$): δ 8.09 (s, 1H), 8.05 (s, 1H), 7.69 (d, *J* = 8.9 Hz, 1H), 7.68 (s, 1H), 7.59 (dd, *J* = 2.0, 8.9 Hz, 1H), 7.47-7.43 (m, 3H), 7.42-7.38 (m, 2H), 0.12 (s, 9H) ppm. $^{13}$C NMR (100 MHz, $CDCl_3$): δ 146.4, 144.1, 138.9, 134.6, 132.9, 131.7, 130.2, 130.1, 129.7, 129.3, 127.9, 127.7, 127.4, 119.7, 0.77 ppm. IR (solid, ATR) 2952, 1570, 1495, 1472, 1443, 1408, 1387, 1172, 1078, 1062, 1029, 1001, 970, 898, 834, 802, 755, 732, 701 cm$^{-1}$. HRMS (EI-TOF) calcd for $C_{19}H_{19}BrSi$ ($[M]^+$) 354.0439, found 354.0433.

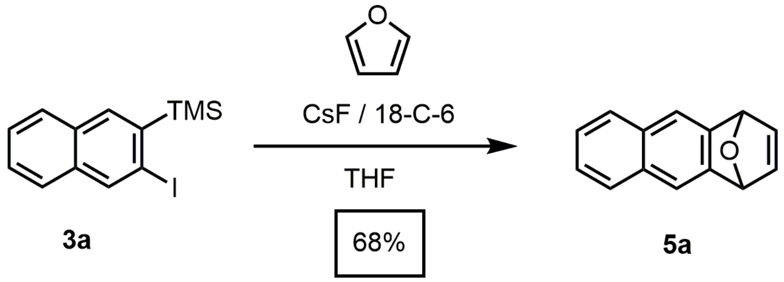


**Synthesis of 5a:** Cesium fluoride (0.052 g, 0.342 mmol) and 18-crown-6 (0.107g, 0.406 mmol) were placed in a flame dried round bottom flask which was evacuated and backfilled with nitrogen. Compound **3a** (0.055 g, 0.169 mmol) and furan (0.12 mL, 1.5 mmol) were then dissolved into anhydrous THF and this solution was added to the reaction flask and let to stir at rt for 12 hours. When the reaction was finished, water (2 mL) was added and it was extracted with $CH_2Cl_2$ (3 × 3 mL). The organic fractions were collected and dried using $MgSO_4$ before being purified by column chromatography using $CH_2Cl_2$ as the eluent. Compound **5a** was isolated as a white solid (0.022 g, 68 % Yield). $^{1}$H and $^{13}$C NMR were consistent with literature values.[5]

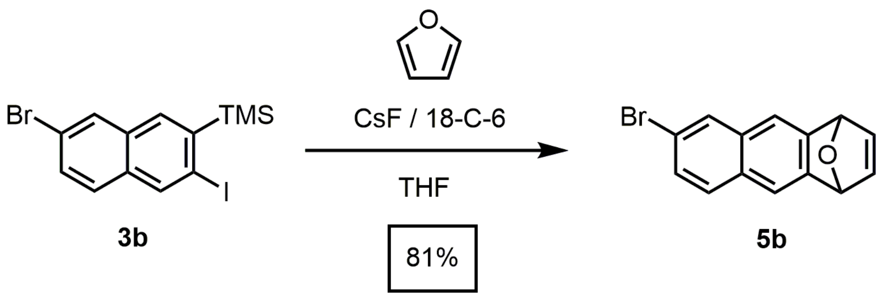

**Synthesis of 5b:** Cesium fluoride (0.018 g, 0.12 mmol) and 18-crown-6 (0.058 g, 0.22 mmol) were placed in a flame dried round bottom flask which was evacuated and backfilled with nitrogen. Compound **3b** (0.022 g, 0.054 mmol) and furan (0.05 mL, 0.7 mmol) were then dissolved into anhydrous acetonitrile and this solution was added to the reaction flask. The reaction was stirred for 24 hours and monitored by TLC. When the reaction was finished, water (2 mL) was added and it was extracted with $CH_2Cl_2$ (3 × 3 mL). The organic fractions were collected, dried using $MgSO_4$, and filtered before being purified by silica gel column chromatography (70% Hexanes : 30 % $CH_2Cl_2$). **5b** was isolated as a white solid (0.011 g, 81 % Yield). $^1$H NMR (500 MHz, $CDCl_3$): δ 7.86 (s, 1H), 7.57 (d, $J$ = 8.47 Hz, 1H), 7.54 (s, 1H), 7.51 (dd, $J$ = 1.93, 8.47 Hz, 1H), 7.49 (s, 1H), 6.97 (s, 2H), 5.80 (s, 1H) ppm, $^{13}$C NMR (125 MHz, $CDCl_3$): δ 145.6, 144.9, 141.8, 141.7, 133.3, 130.6, 130.3, 129.7, 129.5, 120.0, 118.6, 117.9, 81.9, 81.9 ppm. IR (solid, ATR) 3018, 1593, 1495, 1424, 1305, 1285, 1224, 1197, 1147, 1068, 986, 907, 83, 846, 809, 731 cm$^{-1}$. HRMS (ESI) calcd for $C_{14}H_{11}BrO$ ($[M + H]^+$) 272.9910, found 272.9905.

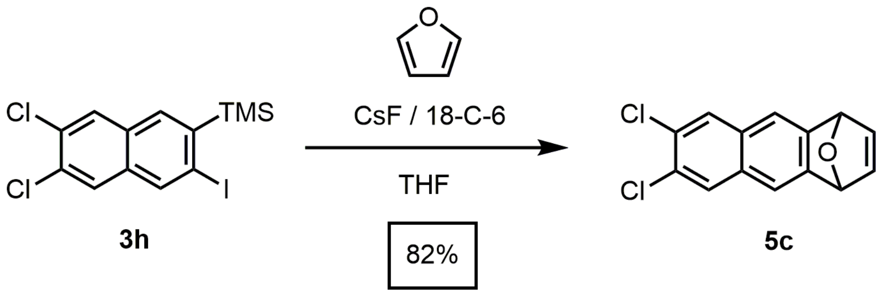

**Synthesis of 5c:** Cesium fluoride (0.019 g, 0.13 mmol) and 18-crown-6 (0.057 g, 0.22 mmol) were placed in a flame dried round bottom flask which was evacuated and backfilled with nitrogen. Compound **3h** (0.020 g, 0.051 mmol) and furan (0.05 mL, 0.7 mmol) were then dissolved into anhydrous acetonitrile and this solution was added to the reaction flask. The reaction was stirred for 24 hours and monitored by TLC. When the reaction was finished, 2 mL of water was added and it was extracted with $CH_2Cl_2$ (3 × 3 mL). The organic fractions were collected, dried using $MgSO_4$, and filtered before being purified by silica gel column chromatography (70% Hexanes : 30 % $CH_2Cl_2$). **5c** was isolated as a white solid (0.011 g, 82 % Yield). $^1$H NMR (500 MHz, $CDCl_3$): δ 7.49 (s, 2H), 7.37 (s, 2H), 6.94 (s, 2H), 5.77 (s, 2H), 2.73 (t, $J$ = 7.64, 4H), 1.66-1.59, (m, 4H), 1.48-1.40 (m, 4H), 0.97 (t, $J$ = 8.01 Hz, 6H) ppm. $^{13}$C NMR (125 MHz, $CDCl_3$) δ 143.3, 141.7, 139.8, 130.5, 127.7, 118.1, 82.0, 33.6, 32.7, 23.0, 14.2 ppm. IR (solid, ATR) 3037, 2928, 2857, 1589, 1475, 1426, 1413, 1399, 1280, 1197, 1176, 1124, 1069, 1059, 981, 965, 910, 877, 861, 847, 737, 705, 661 cm$^{-1}$. HRMS (APPI) calcd for $C_{14}H_7Cl_2O$ ($[M]^-$) 260.9879, found 260.9891.

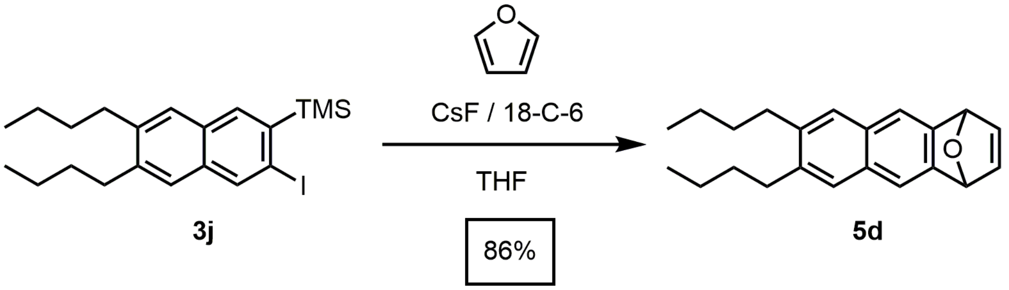


3j 5d

**Synthesis of 5d:** Cesium fluoride (0.023 g, 0.151 mmol) and 18-crown-6 (0.0508 g, 0.192 mmol) were placed in a flame dried round bottom flask which was evacuated and backfilled with nitrogen. Compound **3j** (0.021 g, 0.084 mmol) and furan (0.05 mL, 0.7 mmol) were then dissolved into anhydrous acetonitrile and this solution was added to the reaction flask. The reaction was stirred for 24 hours and monitored by TLC. When the reaction was finished, 2 mL of water was added and it was extracted with $CH_2Cl_2$ (3 × 3 mL). The organic fractions were collected, dried using $MgSO_4$, and filtered before being purified by silica gel column chromatography (70% Hexanes : 30 % $CH_2Cl_2$). **5d** was isolated as a white solid (0.012 g, 86 % Yield). $^1$H NMR (500 MHz, $CDCl_3$): δ 7.49 (s, 2H), 7.37 (s, 2H), 6.94 (s, 2H), 5.77 (s, 2H), 2.73 (t, $J$ = 7.64, 4H), 1.66-1.59, (m, 4H), 1.48-1.40 (m, 4 H), 0.97 (t, $J$ = 8.01 Hz, 6H) ppm. $^{13}$C NMR (125 MHz, $CDCl_3$): δ 143.3, 141.7, 139.8, 130.5, 127.7, 118.1, 82.0, 33.6, 32.7, 23.0, 14.2 ppm. IR (solid, ATR) 2954, 2930, 2868, 1497, 1464, 1377, 1279, 1194, 1148, 1103, 1060, 984, 913, 877, 865, 847, 828, 734, 697 cm$^{-1}$. HRMS (ESI) calcd for $C_{22}H_{27}O$ ($[M + H]^+$) 307.2065, found 307.2056.

**General Procedure for Copper Catalyst:**

Copper (I) cyanide (19.9 mg, 0.222 mmol) was transferred to a flame dried 20 mL round bottom flask, evacuated, and back filled with $N_2$. To this was added 20 mL of anhydrous THF and the flask was cooled to -78 °C. After *n*-BuLi (0.18 mL, 2.5 M in hexanes) was slowly added the flask was warmed to 0 °C for 30 min.

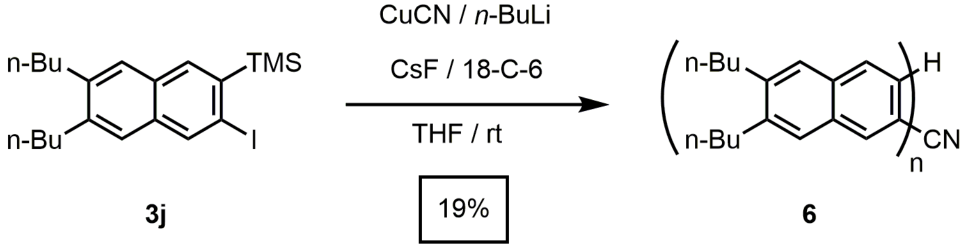


3j 6

**Synthesis of 6:** Cesium fluoride (0.0733 g, 0.483 mmol) was transferred to a vial and flame dried under vacuum and backfilled with $N_2$. Once the reaction vessel was cooled to rt, anhydrous 18-crown-6 (0.243 g, 0.920 mmol) was added to the reaction vessel which was evacuated and backfilled with $N_2$ before being dissolved in anhydrous THF (3 mL). To this stirring solution, a 2 mL solution of **3j** (0.103 g, 0.235 mmol) in anhydrous THF and 0.1 mL of the previously prepared Lipshultz cuprate was added simultaneously. The reaction was allowed to stir for 12 hours until completion. When the reaction had finished, water (5 mL) was added and the reaction is extracted using $CH_2Cl_2$ (3 × 5 mL). The organic fractions were collected and dried using $MgSO_4$. This solution was concentrated under vacuum and **6** was precipitated into MeOH and isolated as a yellow solid (0.011 g, 19%). $^1$H NMR (500 MHz, $CDCl_3$): δ 7.48 (bs, 4H), 2.76 (br, 4H), 1.46 (br, 8H), 0.98 (br, 6H) ppm. $^{13}$C NMR (125 MHz, $CDCl_3$): δ 138.3, 129.8, 125.9, 32.5, 31.7, 22.0, 13.2

ppm. IR (solid, ATR) 2954, 2937, 2859, 1487, 1464, 1377, 1260, 1102, 1021, 908, 837, 804, 734 cm$^{-1}$.

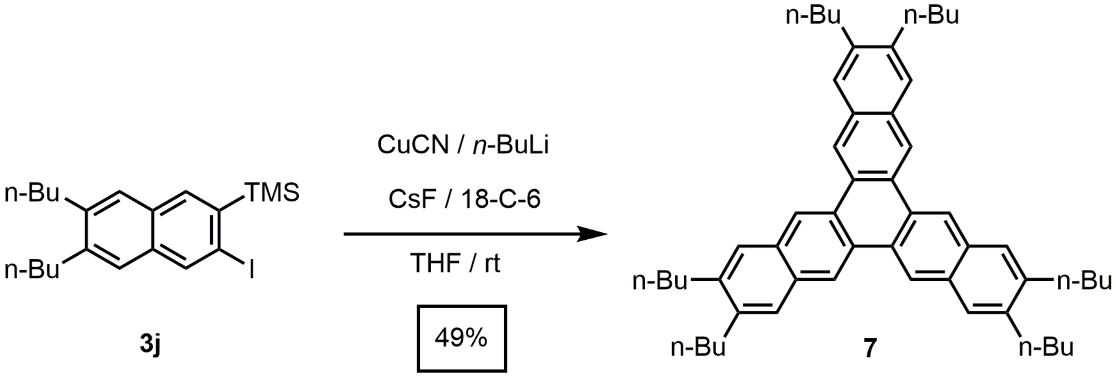


**Synthesis of 7:** Cesium fluoride (0.070 g, 0.461 mmol) was transferred to a vial and flame dried under vacuum and backfilled with $N_2$. Once the reaction vessel was cooled to rt, anhydrous 18-crown-6 (0.255 g, 0.965 mmol) was added to the reaction vessel which was evacuated and backfilled with $N_2$ before being dissolved in 2 mL of anhydrous THF. To this stirring solution, a 1 mL solution of **3j** (0.107 g, 0.249 mmol) in anhydrous THF and 1 mL of the previously prepared Lipshultz cuprate was added simultaneously. The reaction was allowed to stir for 12 hours until completion. When the reaction had finished, water (5 mL) is added and the reaction is extracted using $CH_2Cl_2$ (3 × 5 mL). The organic fractions were collected and dried using $MgSO_4$ before being purified by column chromatography using hexanes as the eluent. **7** was isolated as a red solid (0.027 g, 49%). $^1$H NMR (400 MHz, $CDCl_3$): δ 8.87 (s, 6H), 7.77 (s, 6H), 2.84 (dd, $J$ = 9.48 Hz, $J$ = 7.66 Hz, 12H) 1.82-1.72 (m, 12H), 1.60-1.49 (s, 12H), 1.06 (t, $J$ = 7.31 Hz, 18H) ppm. $^{13}$C NMR (100 MHz, $CDCl_3$): δ 140.1, 131.7, 128.6, 126.9, 121.4, 33.3, 32.8, 23.1, 14.3 ppm IR (solid, ATR) 2953, 2923, 2857, 1599, 1504, 1461, 1394, 1377, 1260, 1182, 1091, 1024, 939, 892, 800, 737, 702 cm$^{-1}$. HRMS (APPI) calcd for $C_{54}H_{67}$ ($[M + H]^+$) 715.5237, found 715.5241.

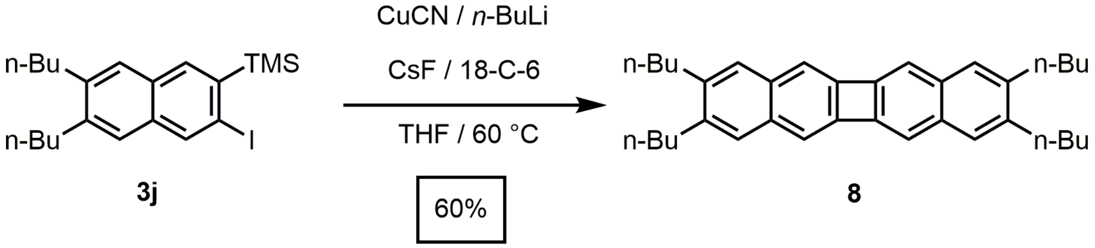


**Synthesis of 8:** Cesium fluoride (0.074 g, 0.487 mmol) was transferred to a vial and flame dried under vacuum and backfilled with $N_2$. Once the reaction vessel was cooled to rt, anhydrous 18-crown-6 (0.254 g, 0.962 mmol) was added to the reaction vessel which was evacuated and backfilled with $N_2$ before being dissolved in anhydrous THF (2 mL). To this stirring solution, a 1 mL solution of **3j** (0.106 g, 0.242 mmol) in anhydrous THF and 1 mL of the previously prepared Lipshultz cuprate was added simultaneously. The reaction is placed in a 60 °C oil bath and allowed to stir for 12 hours to completion. After the reaction has cooled to rt, water (5 mL) is added and the reaction is extracted using $CH_2Cl_2$ (3 × 5 mL). The organic fractions were collected and dried using $MgSO_4$ before being purified by silica gel column chromatography (100% hexanes to 70:30 Hexanes/$CH_2Cl_2$). **8** was isolated as a yellow solid (0.034 g, 60 %). $^1$H NMR (400 MHz, $CDCl_3$): δ 7.35 (s, 4H), 7.15 (s, 4H), 2.69 (dd, $J$ = 9.66 Hz, $J$ = 6.25 Hz, 8H), 1.69-1.59 (m, 8H), 1.51-1.40

(m, 8H), 0.99 (t, *J* = 7.37 Hz, 12H) ppm. $^{13}C$ NMR (100 MHz, $CDCl_3$): δ 145.9, 139.8, 133.8, 128.6, 116.2, 33.5, 32.6, 23.0, 14.2 ppm. IR (solid, ATR) 2956, 2925, 2857, 1486, 1456, 1372, 1325, 1262, 1196, 1148, 1096, 1033, 936, 900, 889, 977, 845, 802, 756, 737 $cm^{-1}$. HRMS (APPI) calcd for $C_{36}H_{45}$ ($[M + H]^+$) 476.3438, found 476.3441.

## C. 1D NMR Spectroscopy

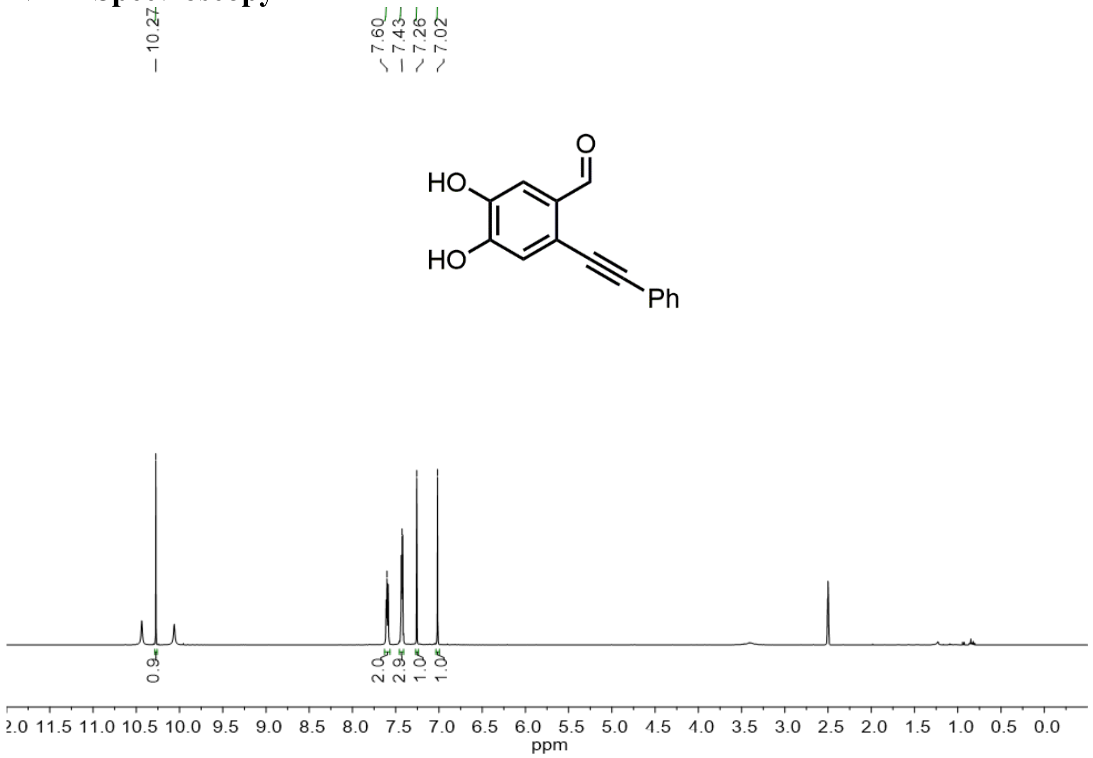


**Figure S1.** $^{1}$H NMR of **S1** (400 MHz, DMSO-$d_6$, 298 K)

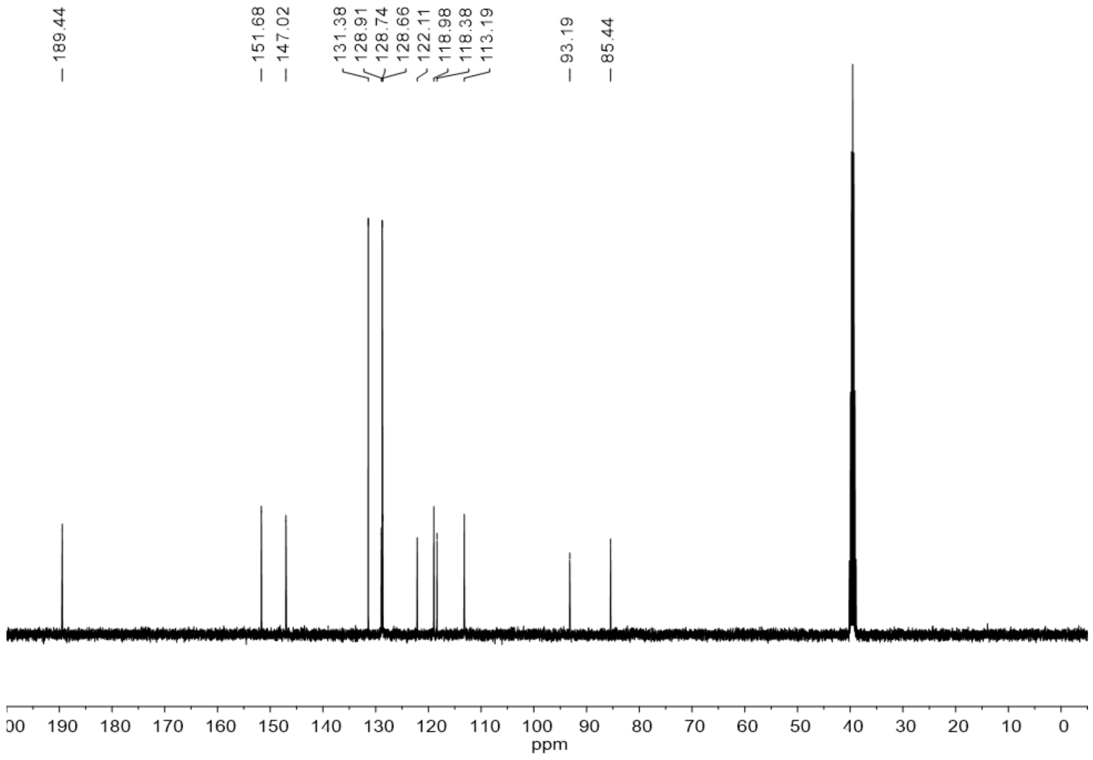


**Figure S2.** $^{13}$C NMR of **S1** (100 MHz, DMSO-$d_6$, 298 K)

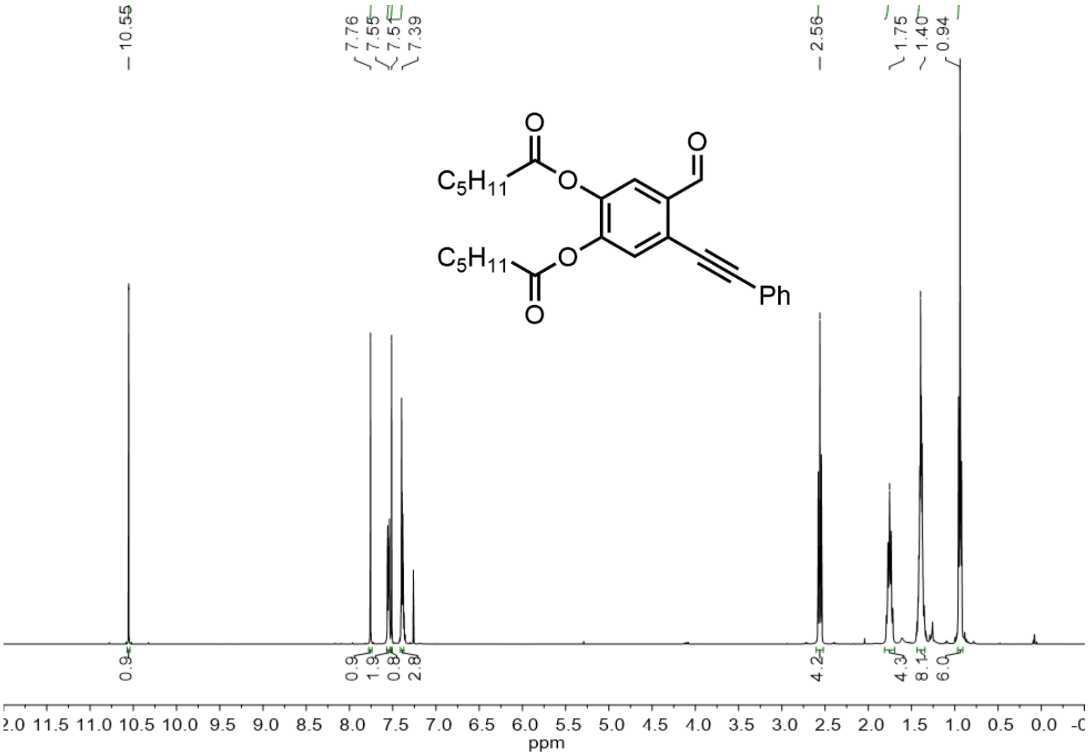


**Figure S3.** $^{1}$H NMR of **1g** (400 MHz, $CDCl_3$, 298 K)

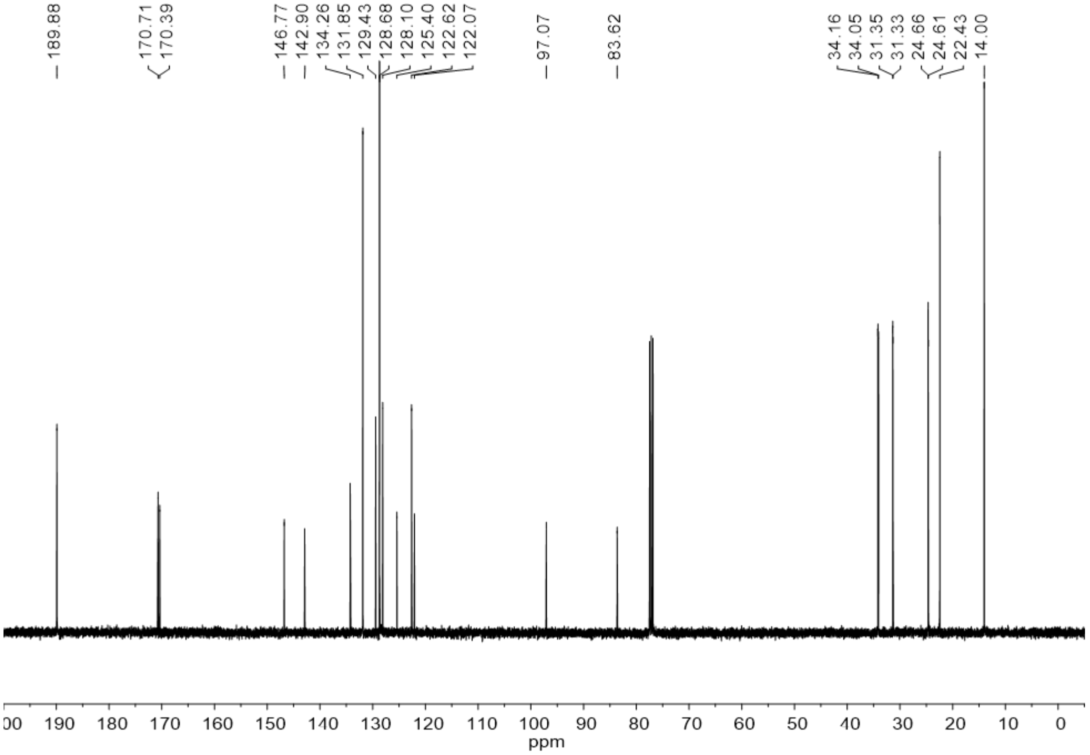


**Figure S4.** $^{13}$C NMR of **1g** (100 MHz, $CDCl_3$, 298 K)

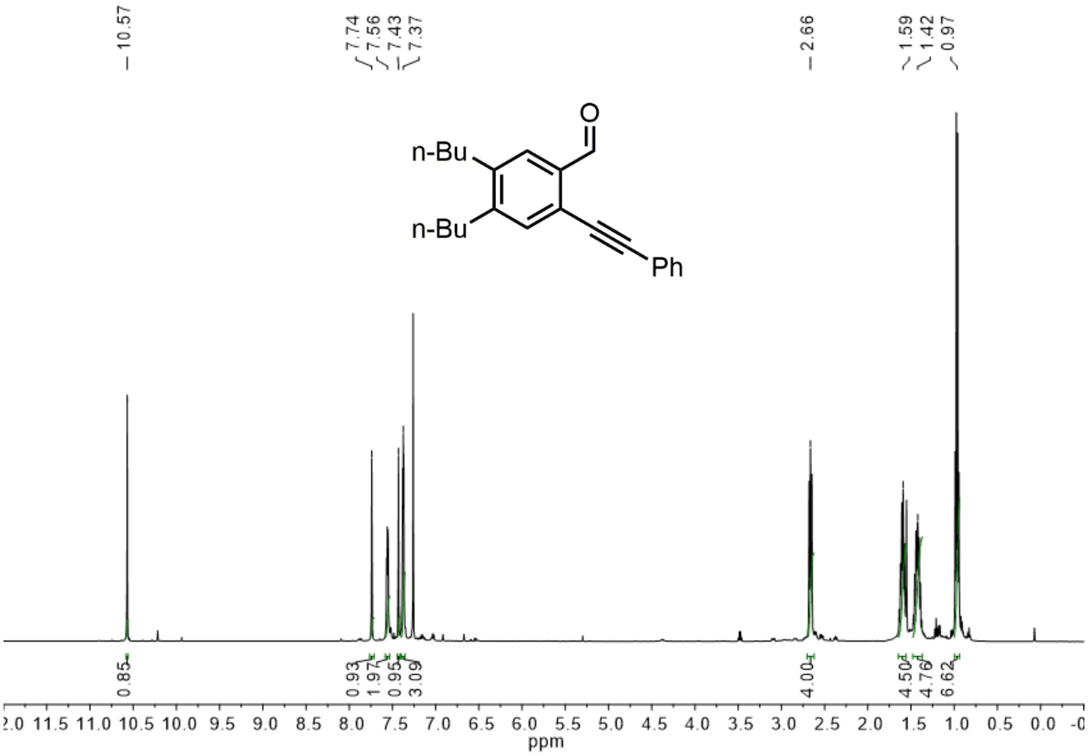


**Figure S5.** $^{1}H$ NMR of **1h** (500 MHz, $CD_3OD$, 298 K)

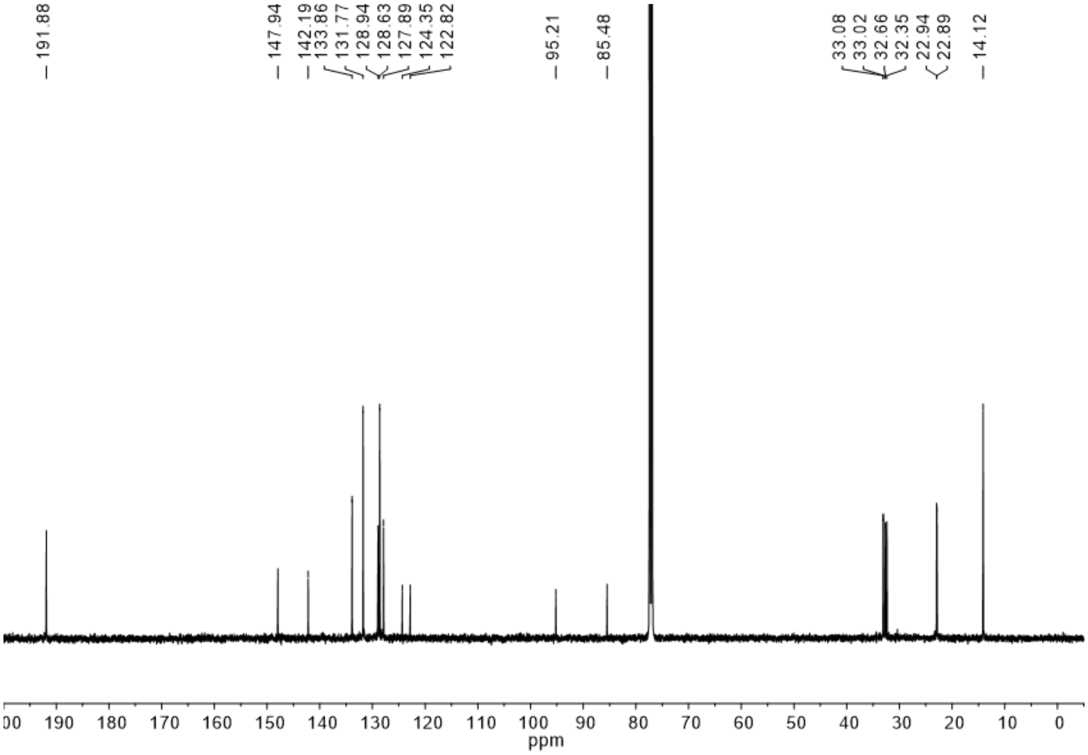


**Figure S6** $^{13}C$ NMR of **1h** (125 MHz, $CDCl_3$, 298 K)

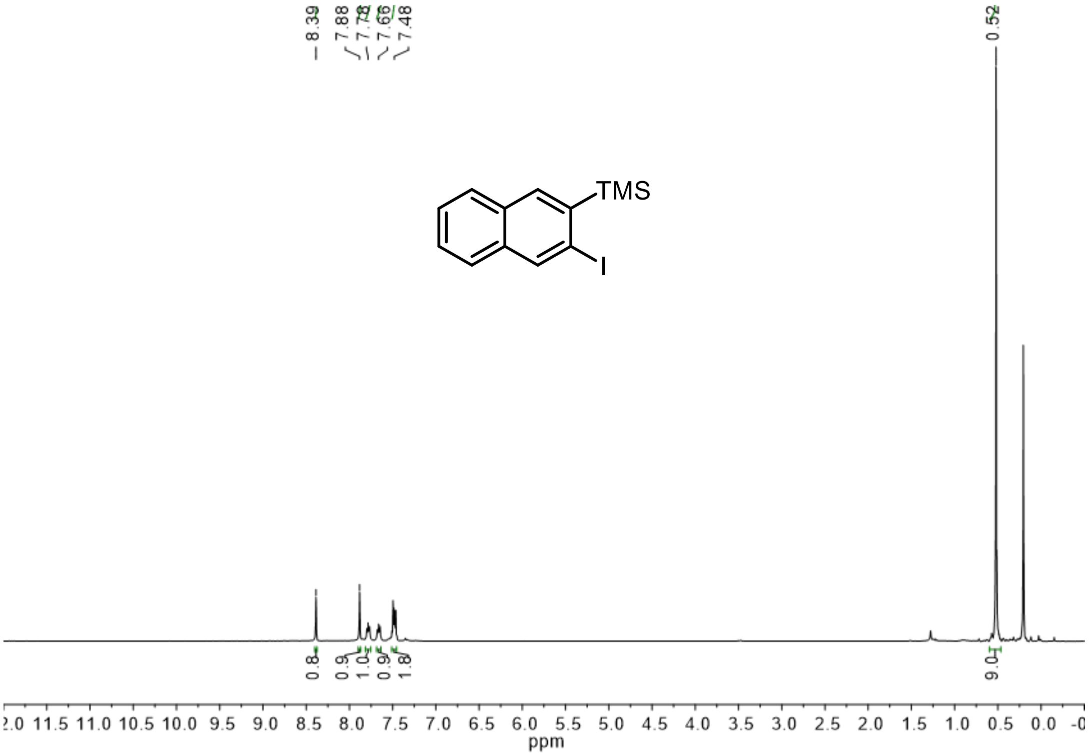


**Figure S7.** $^{1}$H NMR of **3a** (400 MHz, $CDCl_3$, 298 K)

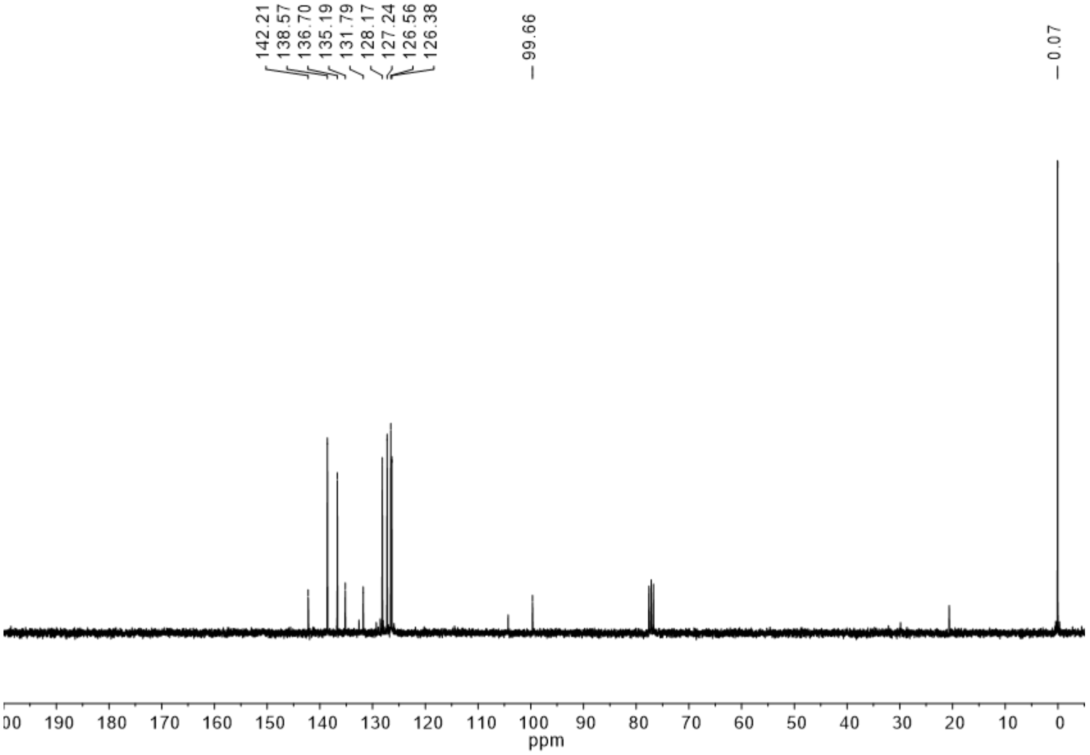


**Figure S8.** $^{13}$C NMR of **3a** (100 MHz, $CDCl_3$, 298 K)

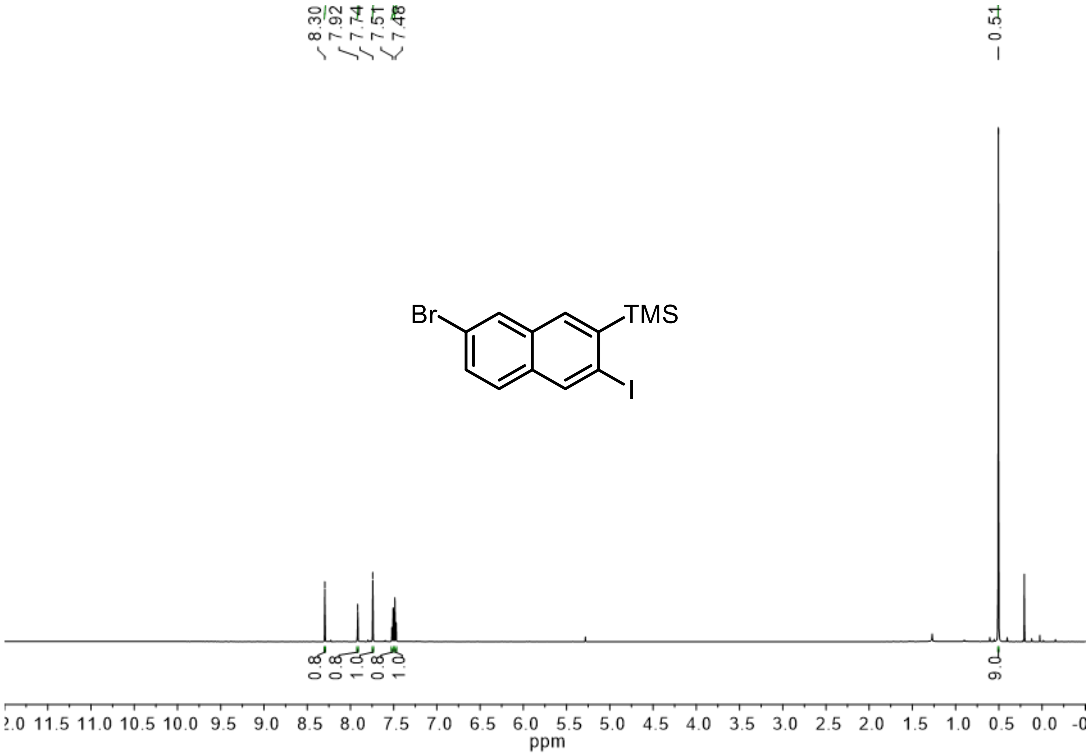


**Figure S9.** $^{1}$H NMR of **3b** (600 MHz, $CDCl_3$, 298 K)

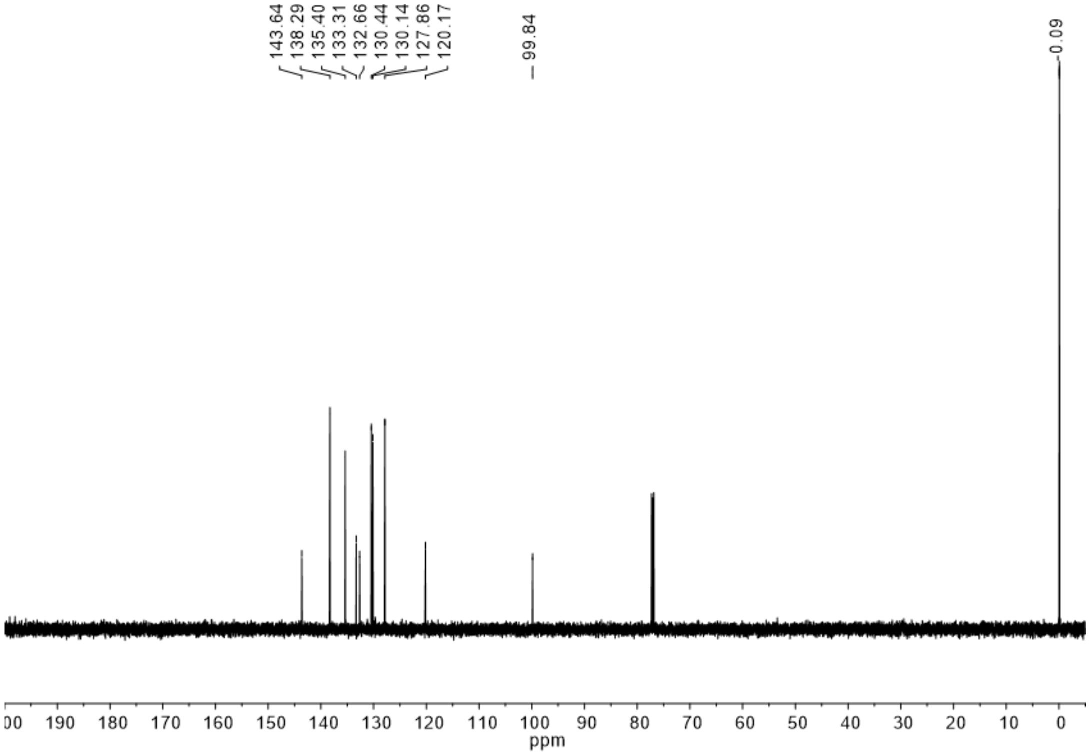


**Figure S10.** $^{13}$C NMR of **3b** (150 MHz, $CDCl_3$, 298 K)

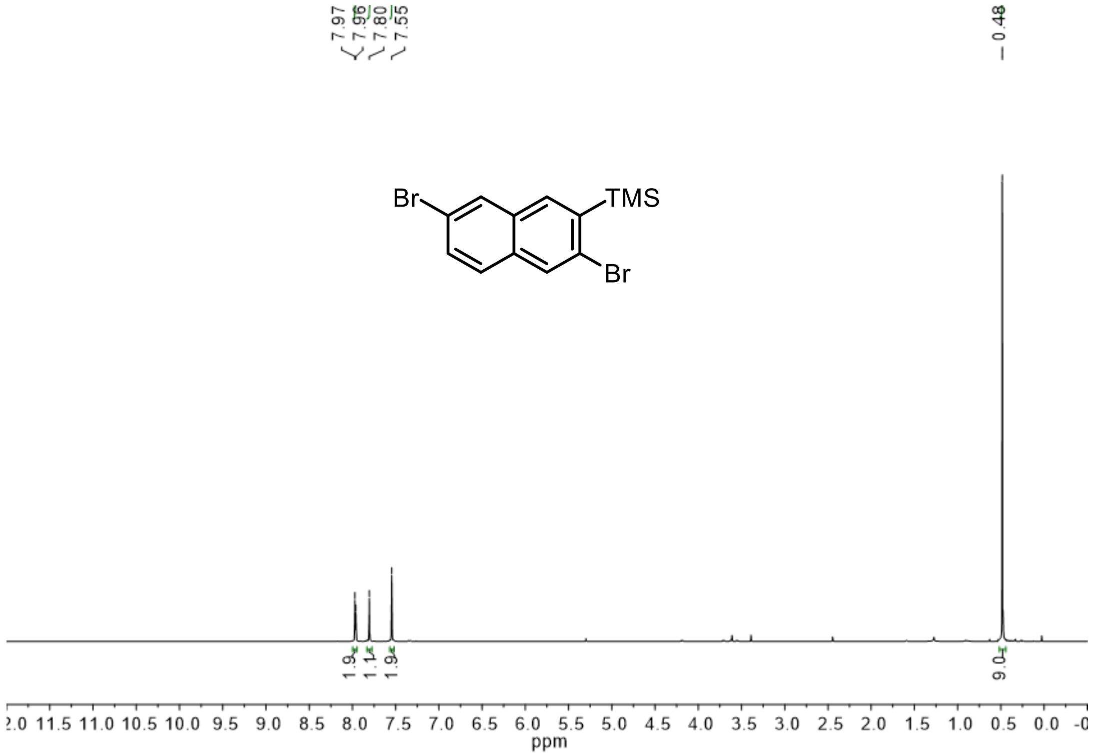


**Figure S11.** $^1$H NMR of **3c** (400 MHz, $CDCl_3$, 298 K)

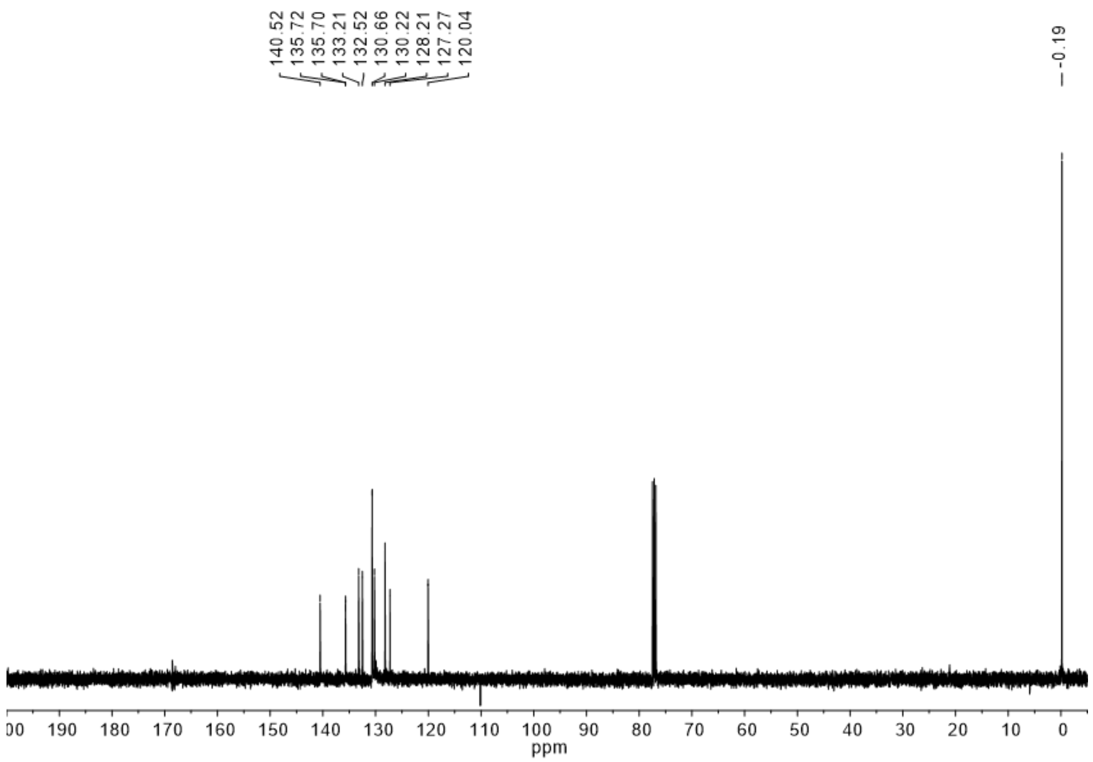


**Figure S12.** $^1$H NMR of **3c** (100 MHz, $CDCl_3$, 298 K)

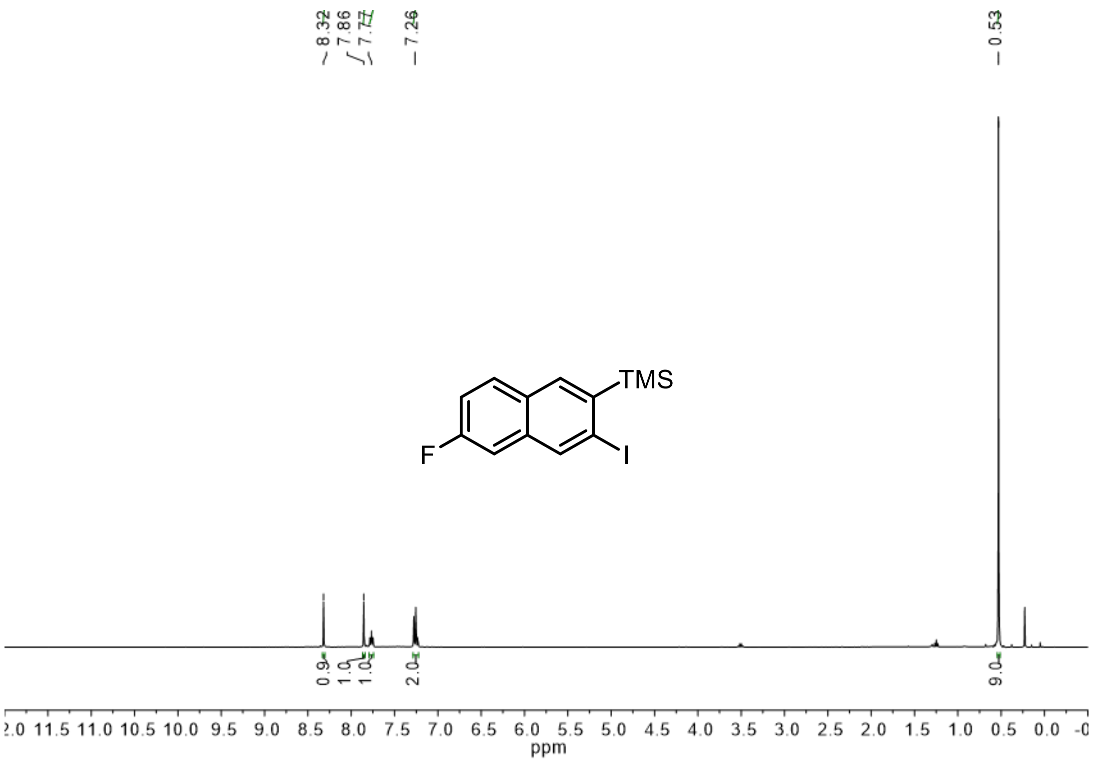


**Figure S13.** $^{1}$H NMR of **3d** (400 MHz, $CDCl_3$, 298 K)

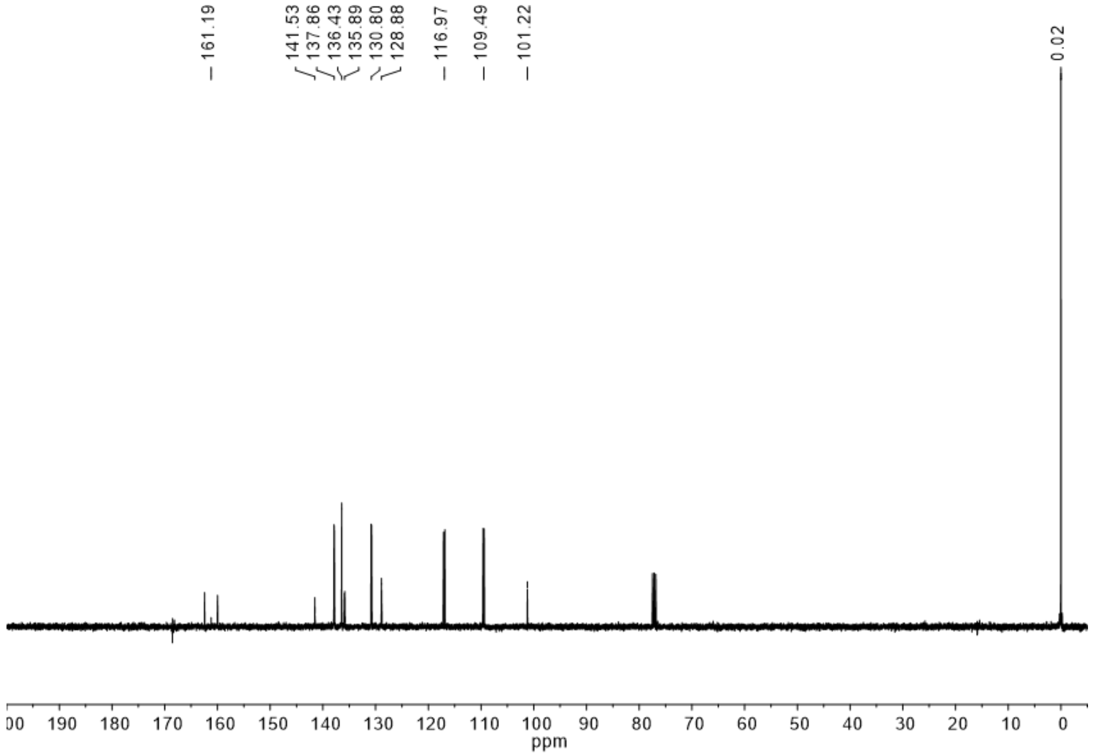


**Figure S14.** $^{13}$C NMR of **3d** (100 MHz, $CDCl_3$, 298 K)

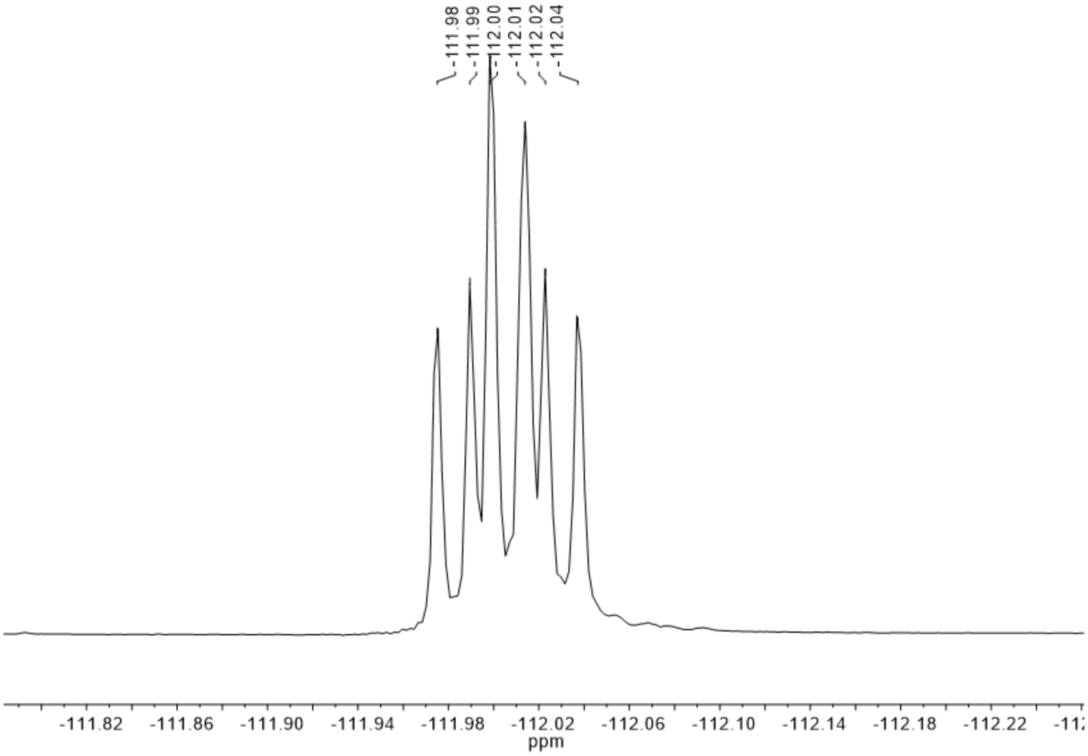


**Figure S15.** $^{19}$F NMR of **3d** (375 MHz, $CDCl_3$, 298 K)

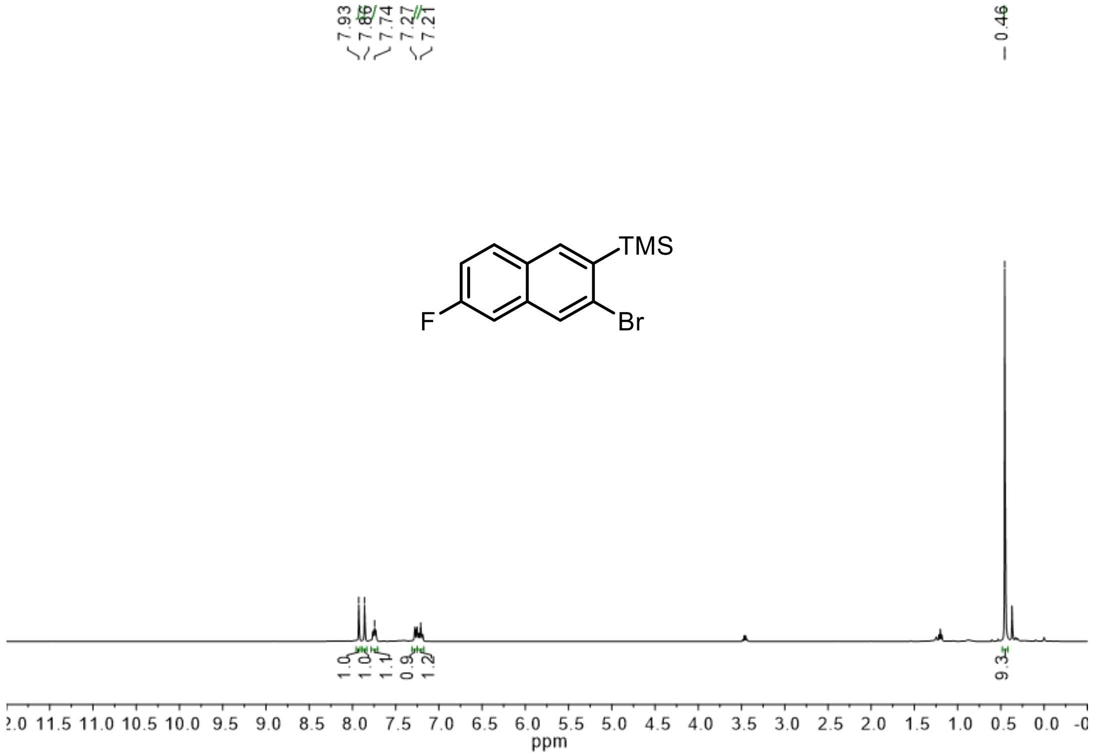


**Figure S16.** $^{1}$H NMR of **3e** (400 MHz, $CDCl_3$, 298 K)

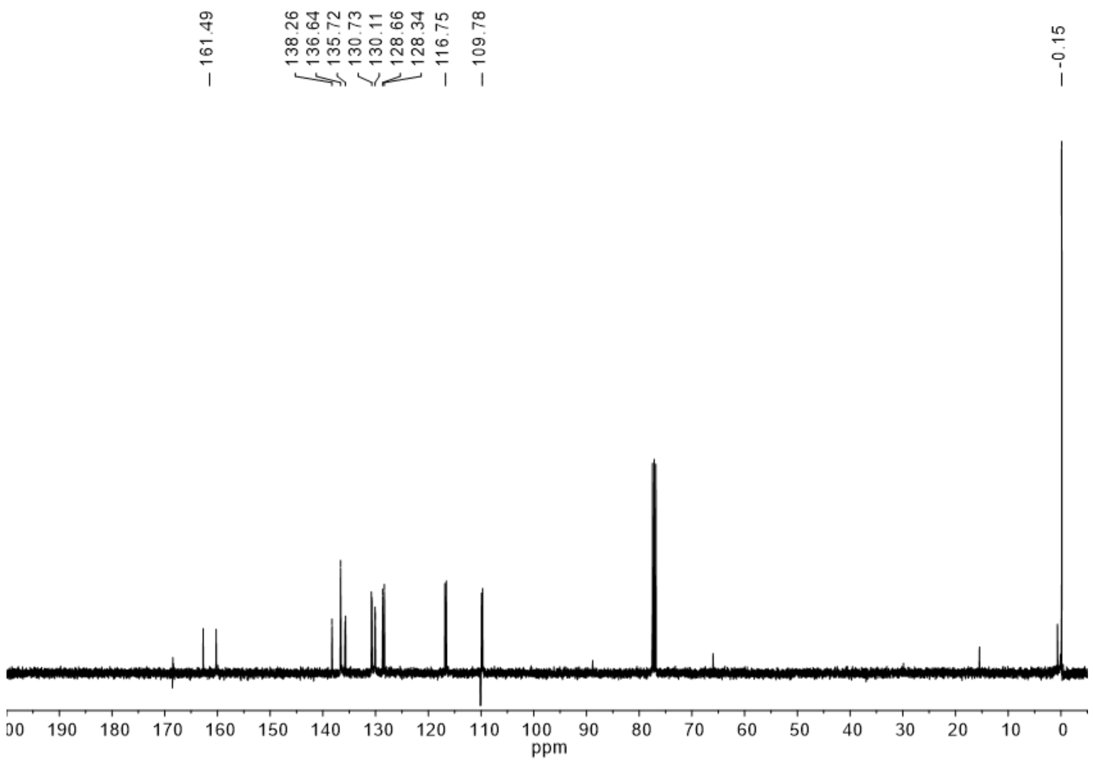


**Figure S17.** $^{13}$C NMR of **3e** (100 MHz, $CDCl_3$, 298 K)

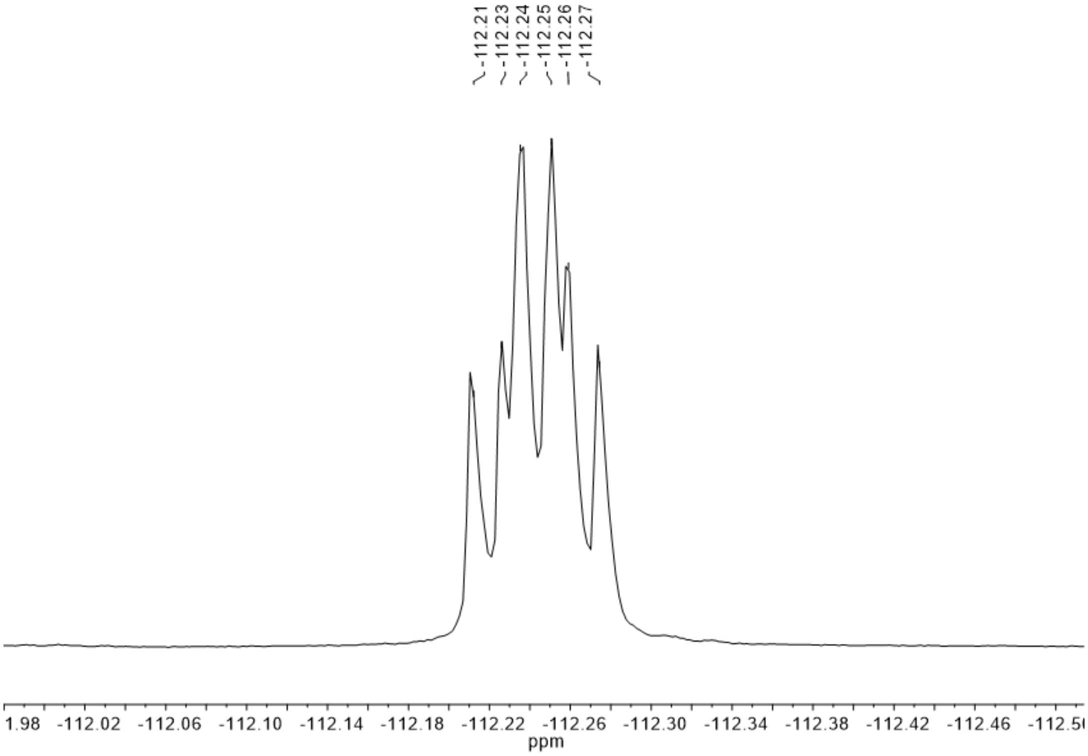


**Figure S18.** $^{19}$F NMR of **3e** (375 MHz, $CDCl_3$, 298 K)

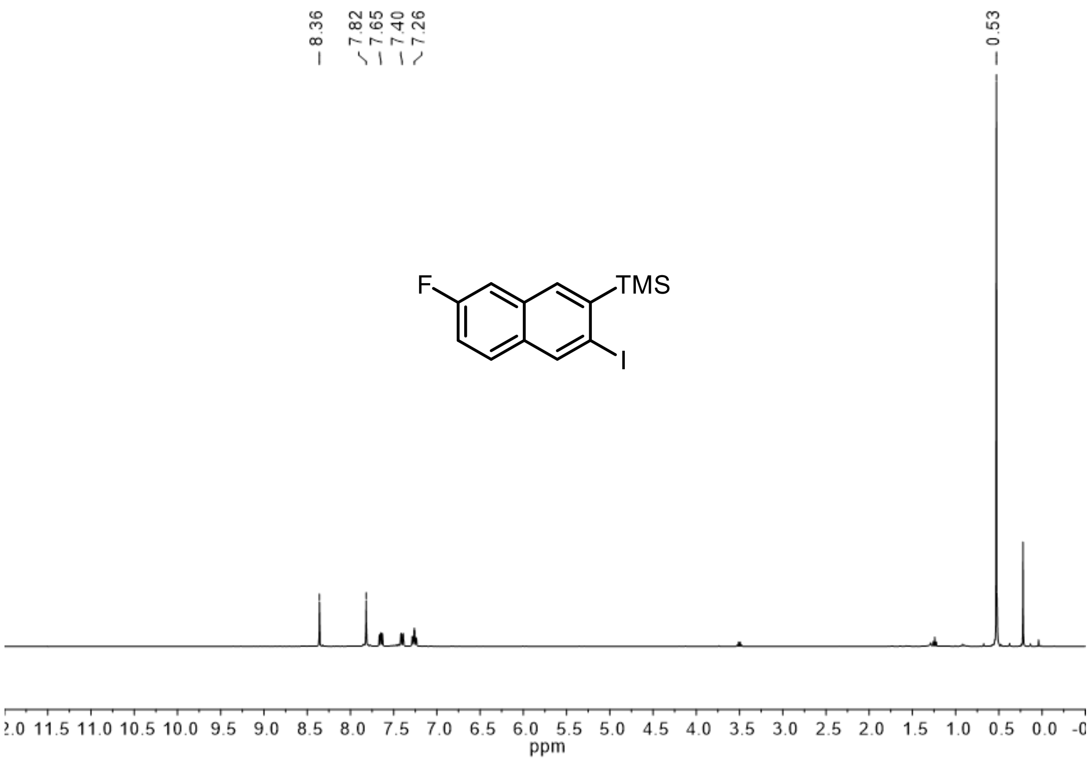


**Figure S19.** $^{1}$H NMR of **3f** (400 MHz, $CDCl_3$, 298 K)

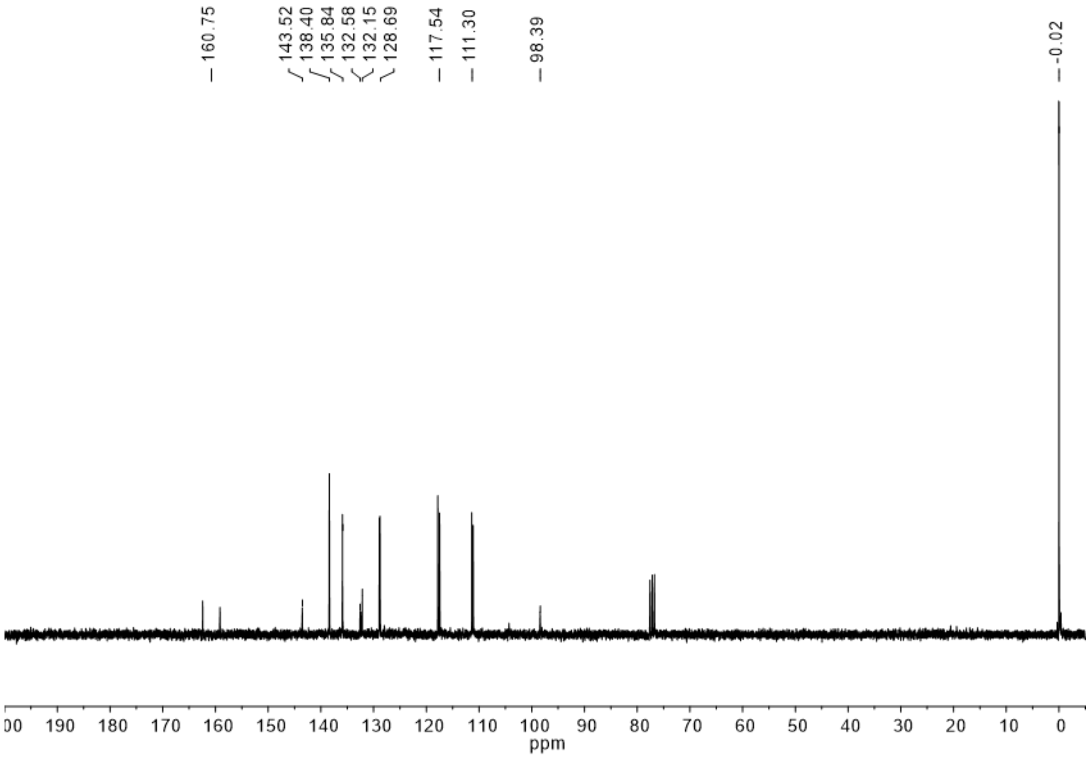


**Figure S20.** $^{13}$C NMR of **3f** (100 MHz, $CDCl_3$, 298 K)

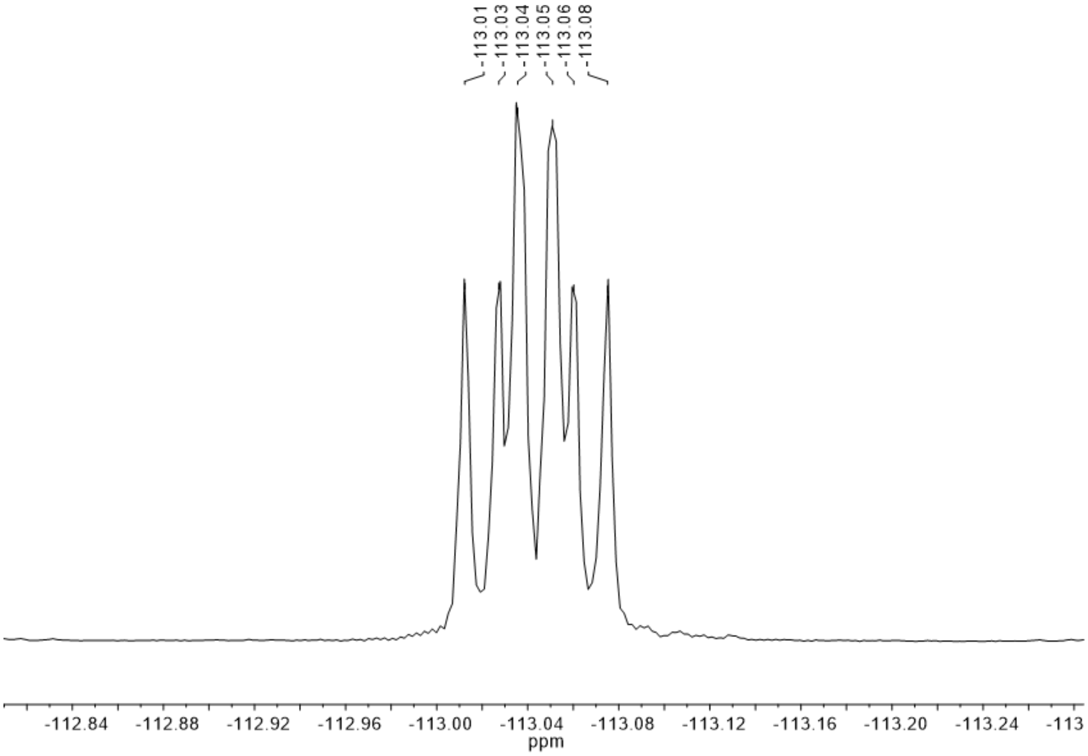


**Figure S21.** $^{19}F$ NMR of **3f** (375 MHz, $CDCl_3$, 298 K)

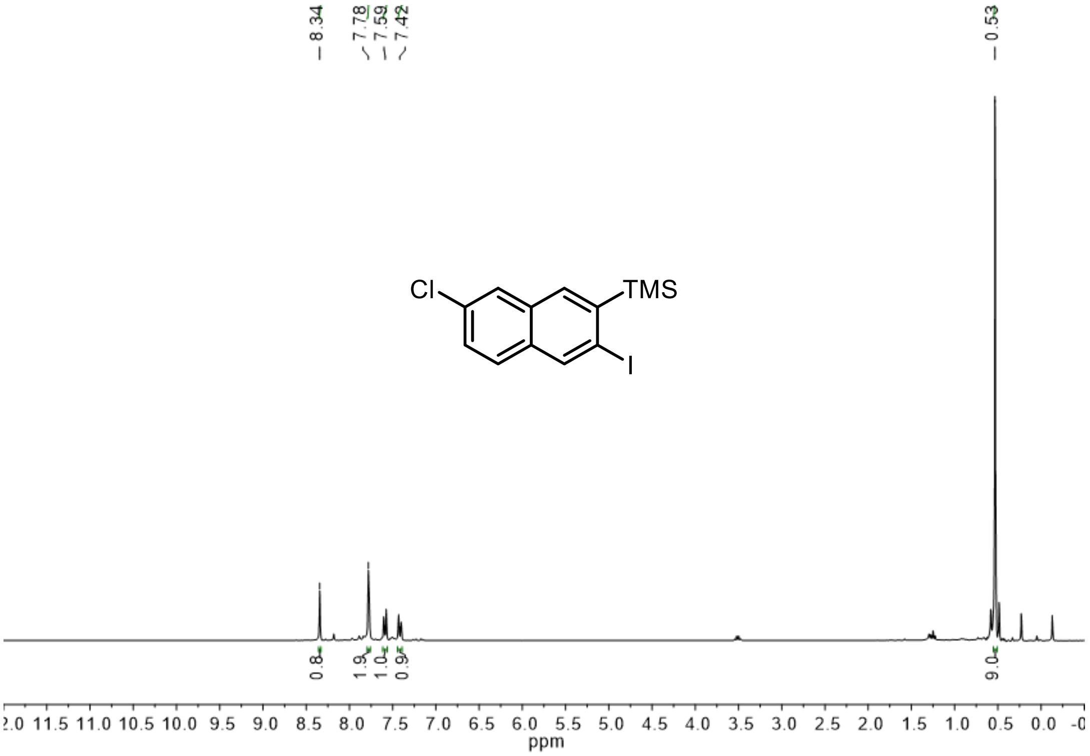


**Figure S22.** $^{1}$H NMR of **3g** (300 MHz, $CDCl_3$, 298 K)

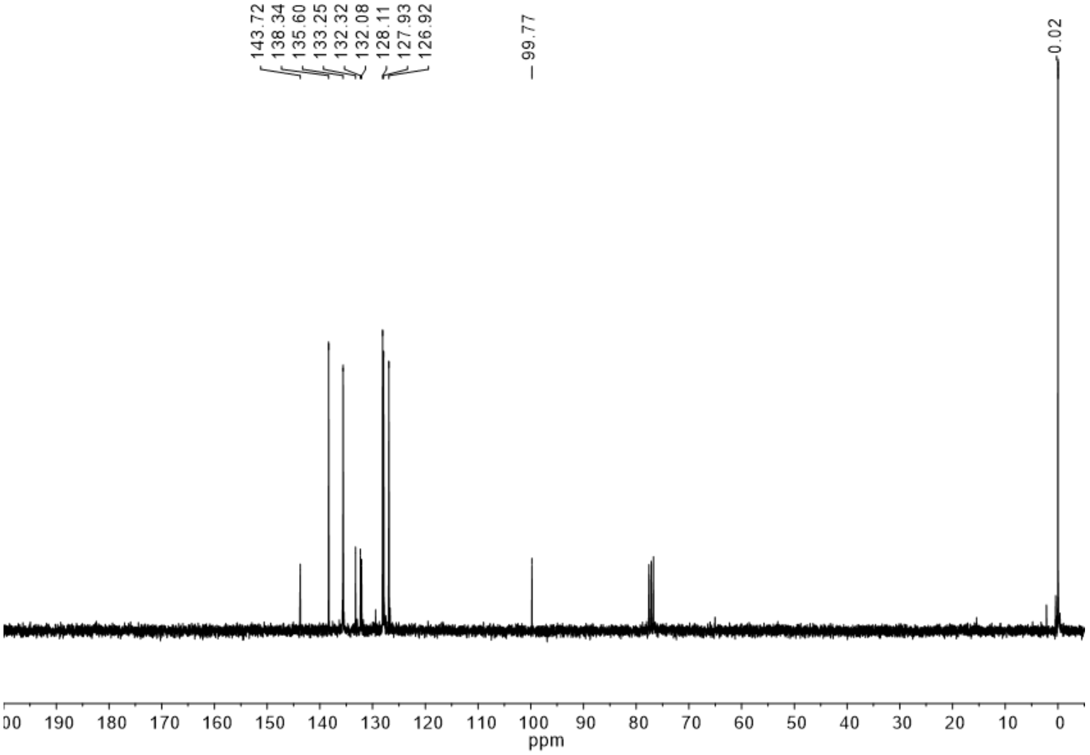


**Figure S23.** $^{13}$C NMR of **3g** (75 MHz, $CDCl_3$, 298 K)

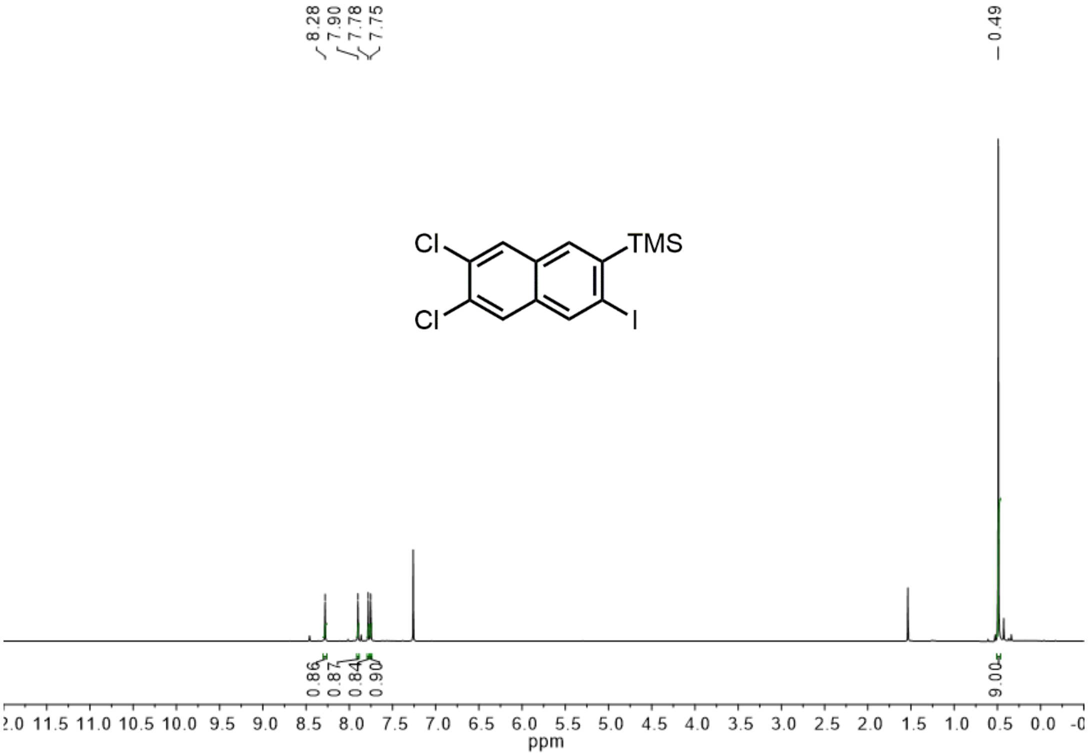


**Figure S24.** $^{1}$H NMR of **3h** (500 MHz, $CDCl_3$, 298 K)

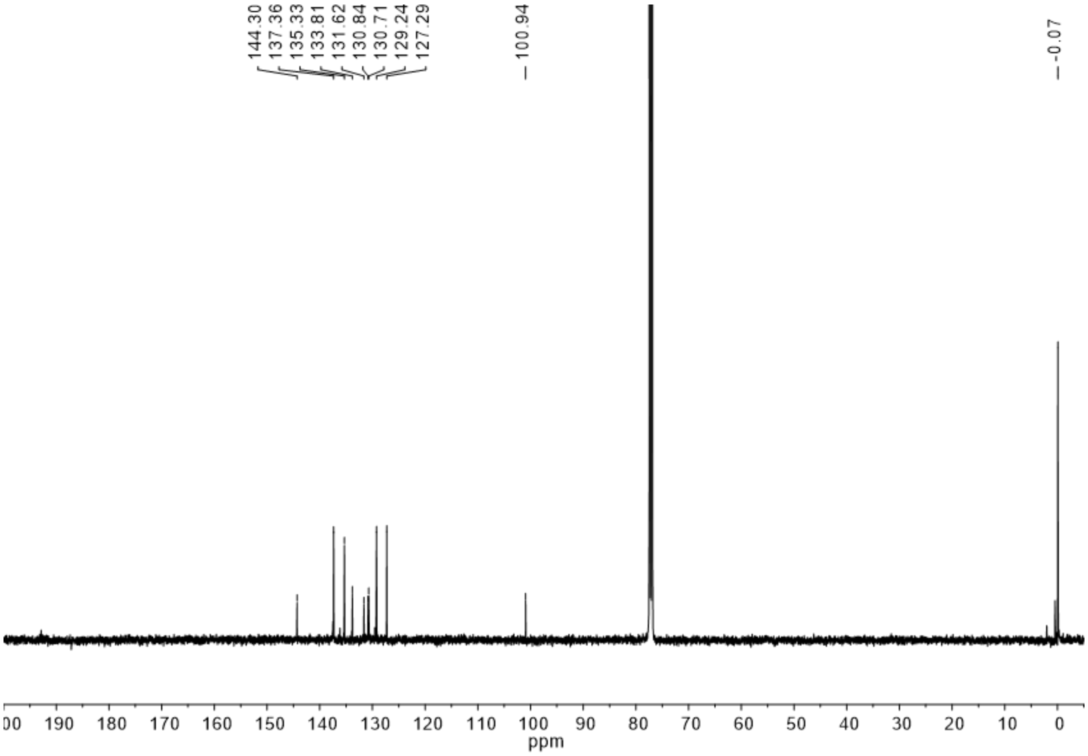


**Figure S25.** $^{13}$C NMR of **3h** (125 MHz, $CDCl_3$, 298 K)

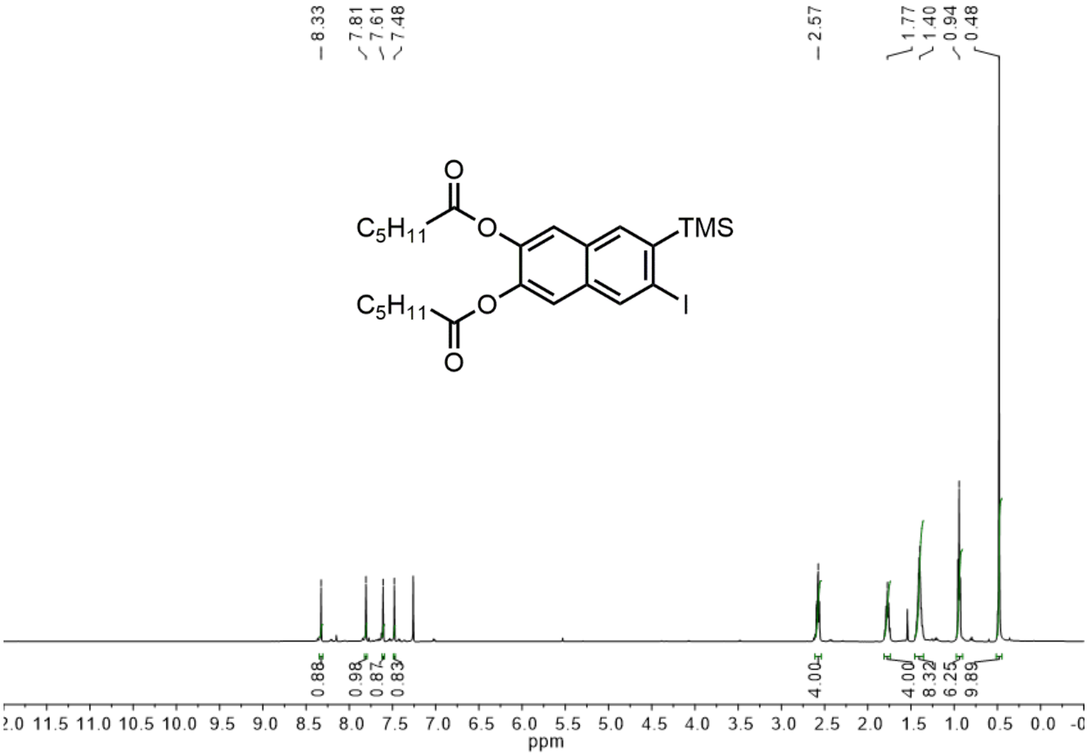

**Figure S26.** $^{1}H$ NMR of **3i** (500 MHz, $CDCl_3$, 298 K)

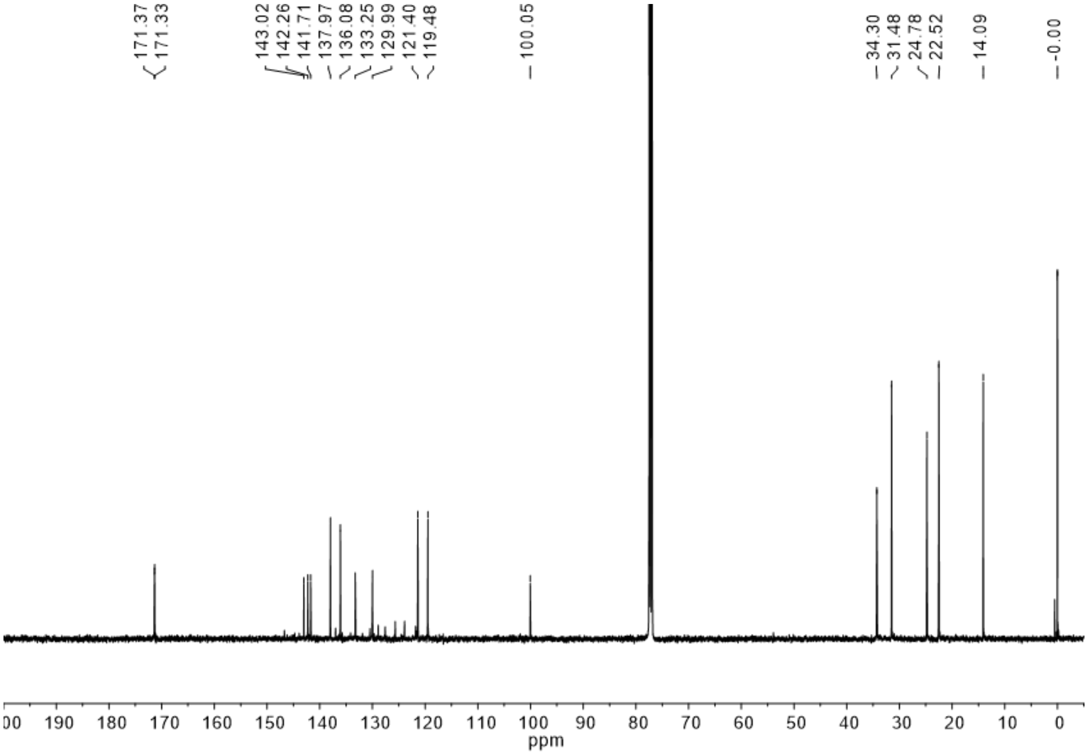

**Figure S27.** $^{13}C$ NMR of **3i** (125 MHz, $CDCl_3$, 298 K)

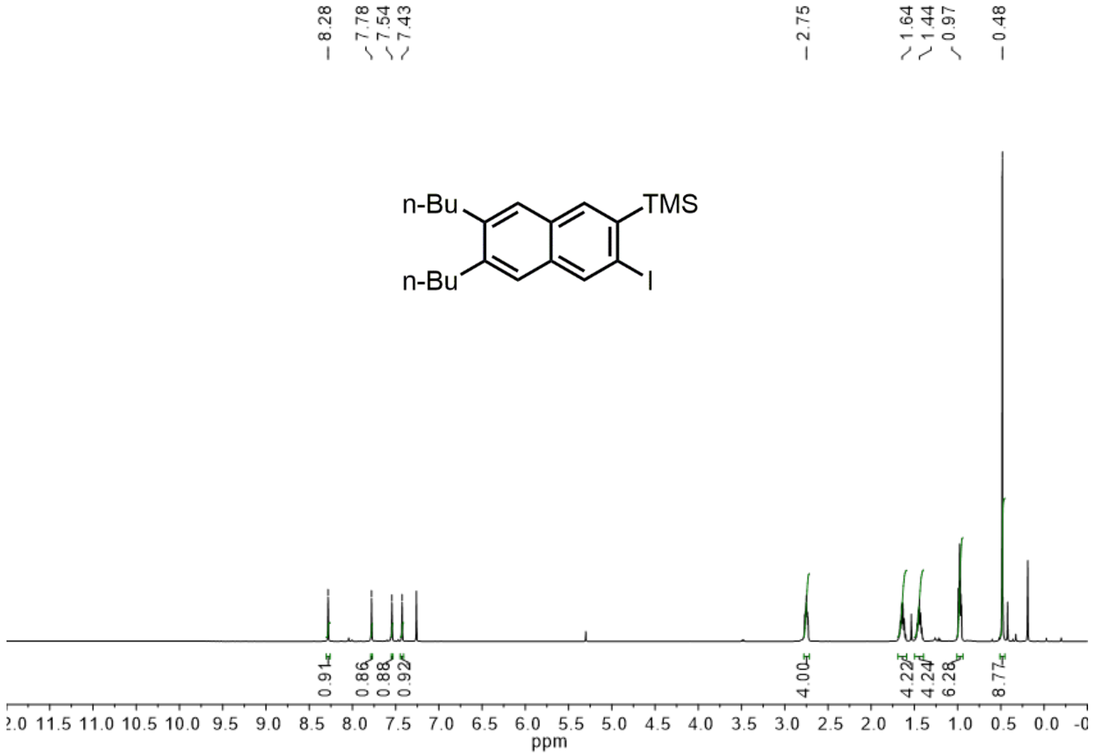


**Figure S28.** $^{1}$H NMR of **3j** (500 MHz, $CDCl_3$, 298 K)

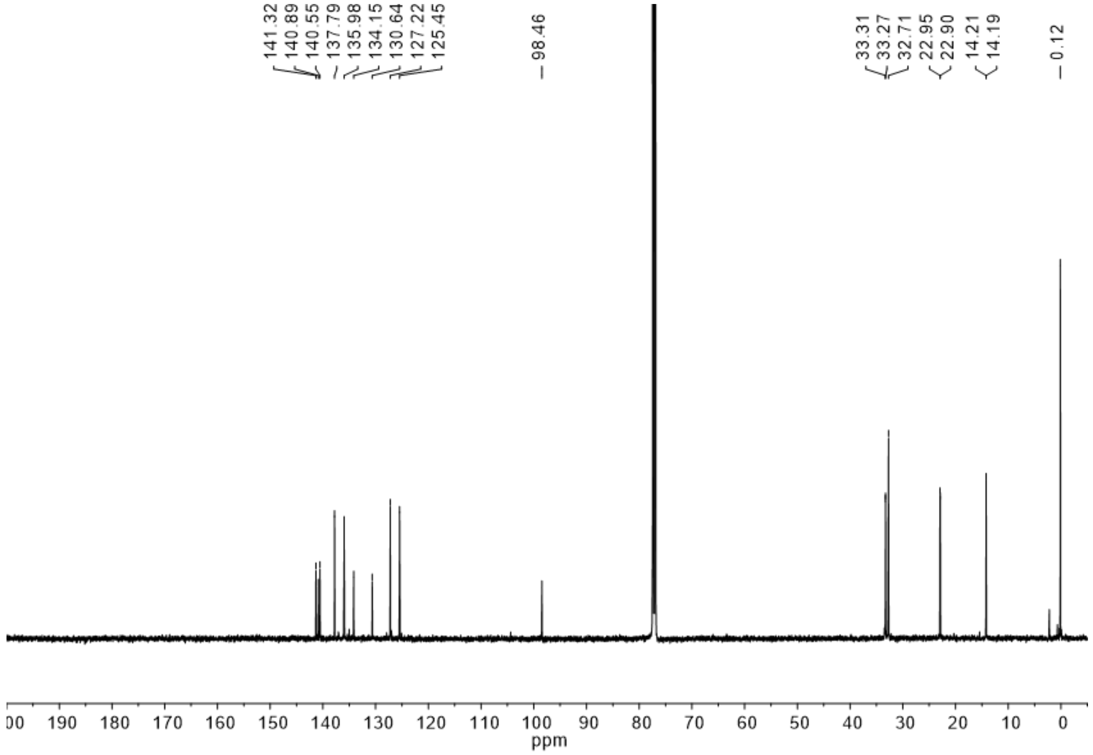


**Figure S29.** $^{13}$C NMR of **3j** (125 MHz, $CDCl_3$, 298 K)

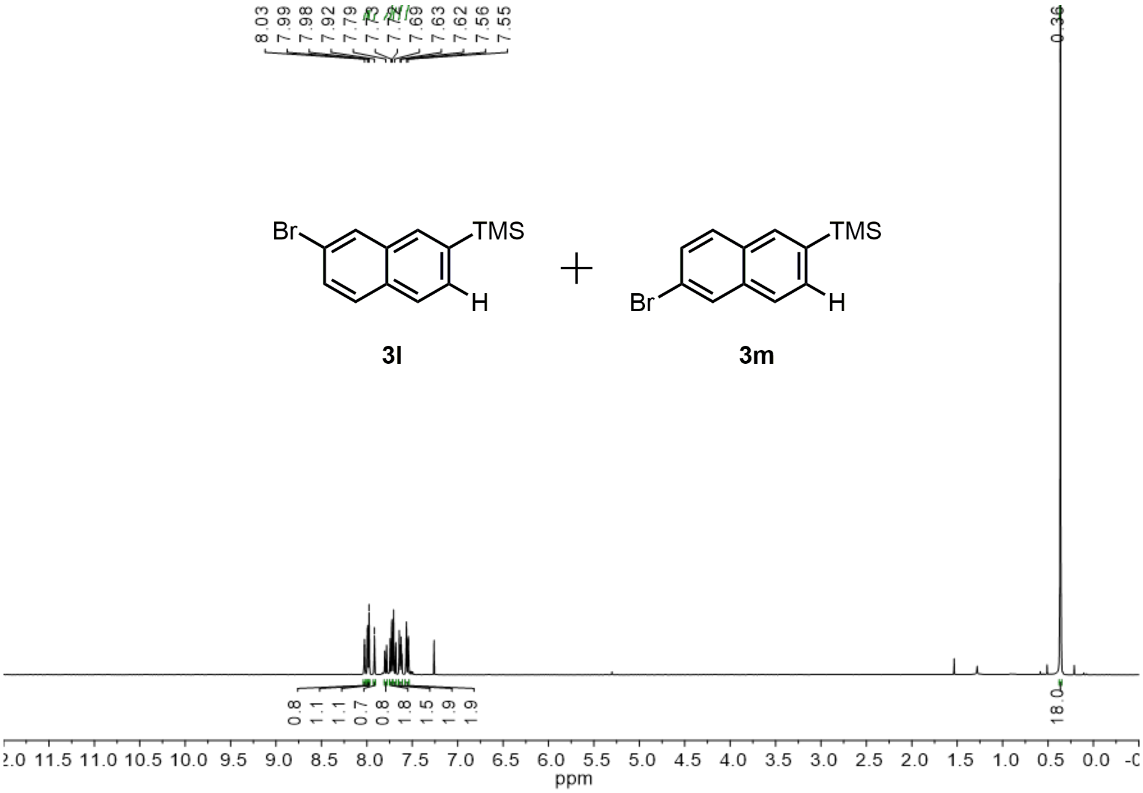


**Figure S30.** $^{1}$H NMR of **3l and 3m** (400 MHz, $CDCl_3$, 298 K)

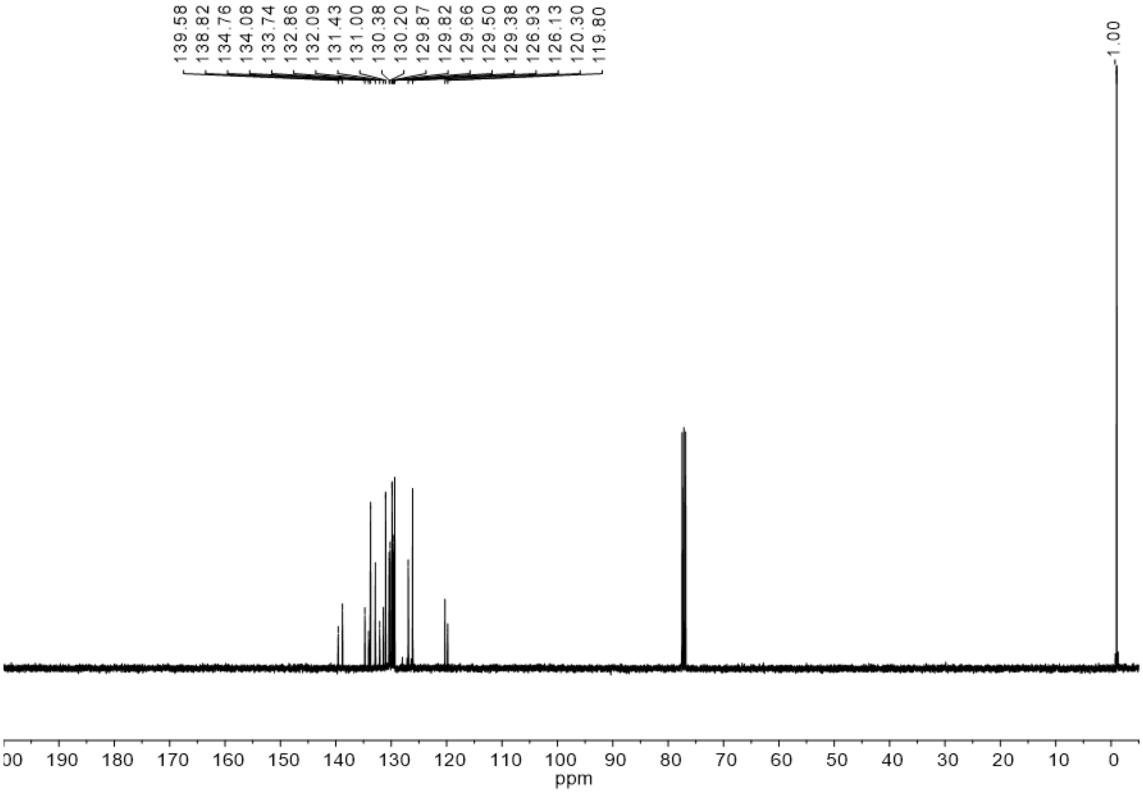


**Figure S31.** $^{13}$C NMR of **3l and 3m** (100 MHz, $CDCl_3$, 298 K)

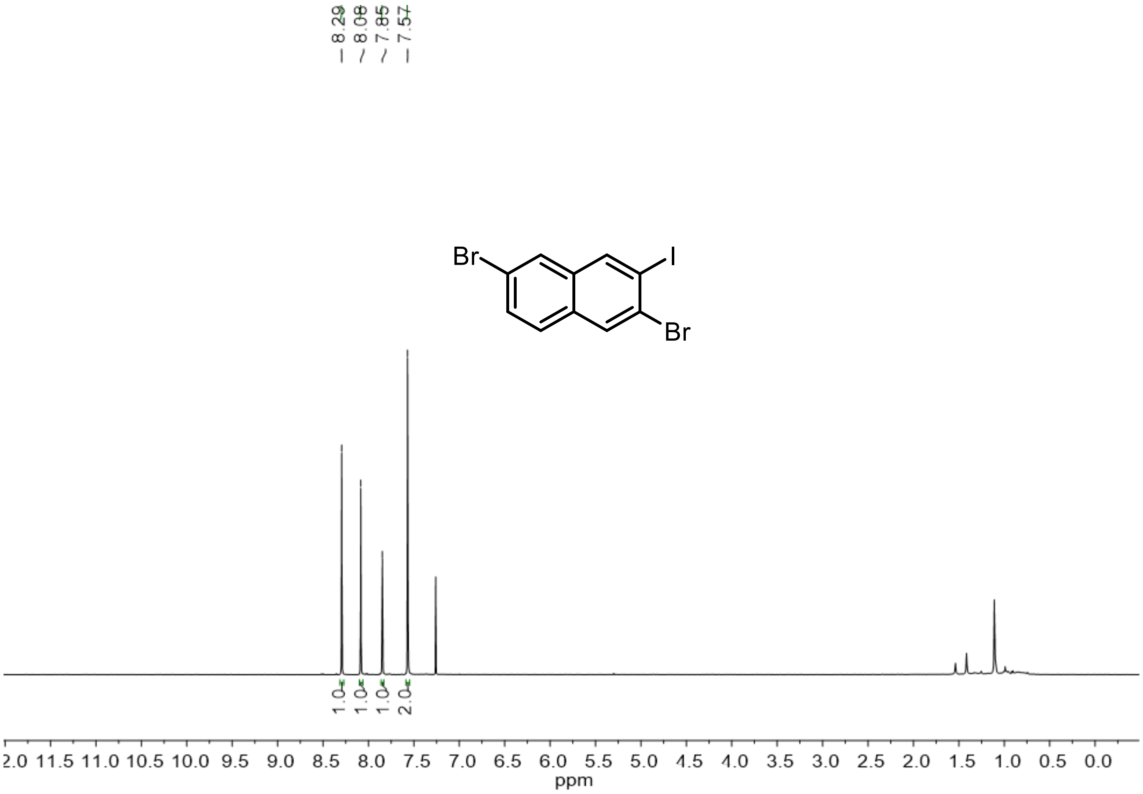


**Figure S31.** $^{1}$H NMR of **4** (400 MHz, $CDCl_3$, 298 K)

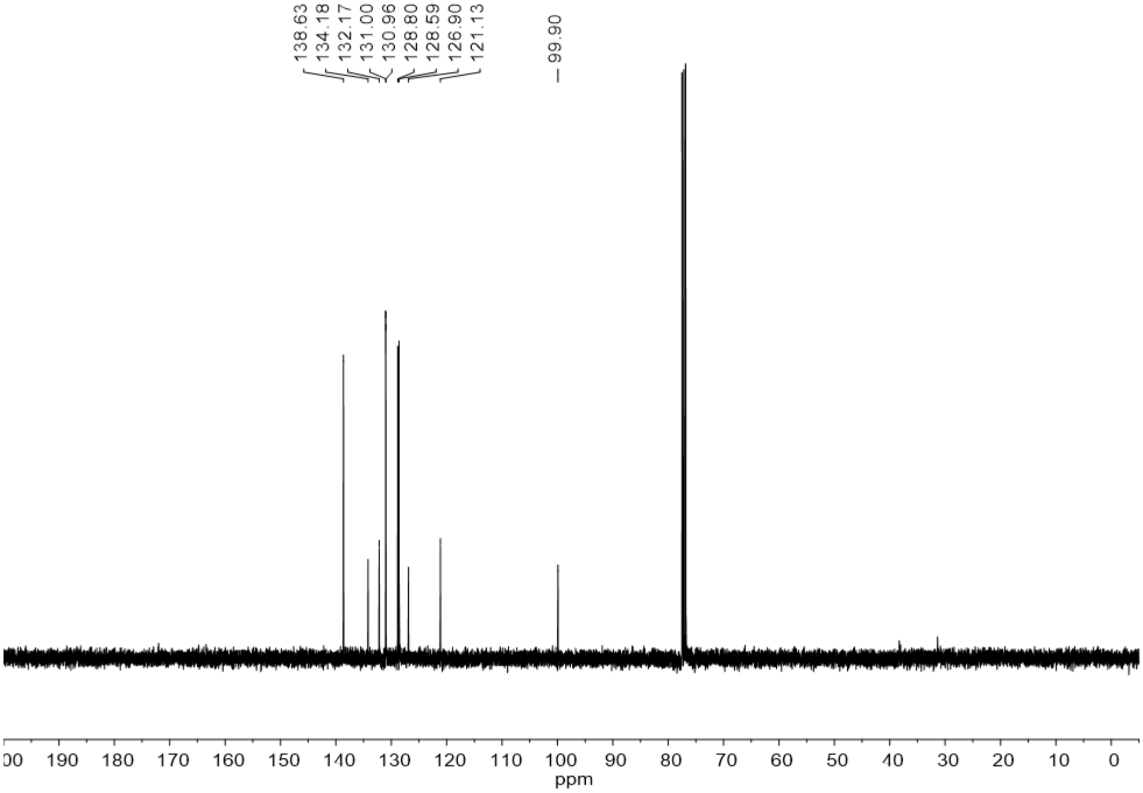


**Figure S32.** $^{13}$C NMR of **4** (100 MHz, $CDCl_3$, 298 K)

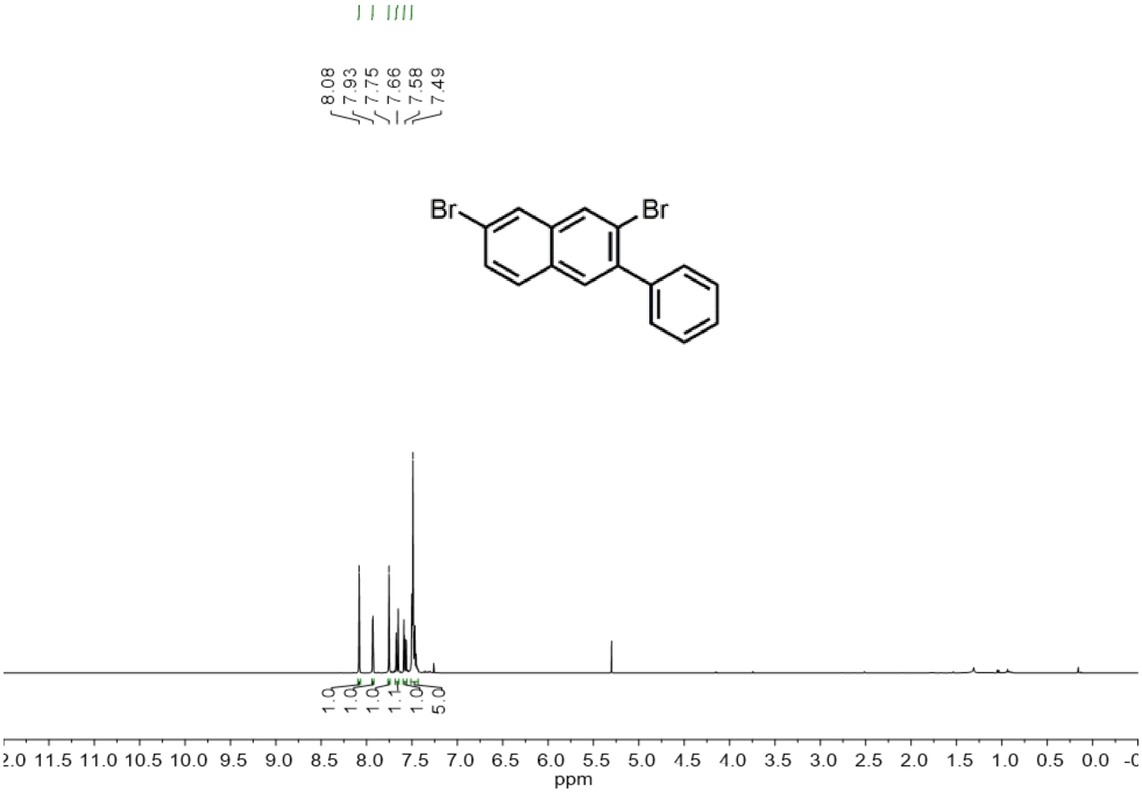


**Figure S33.** $^{1}H$ NMR of **S4** (400 MHz, $CDCl_3$, 298 K)

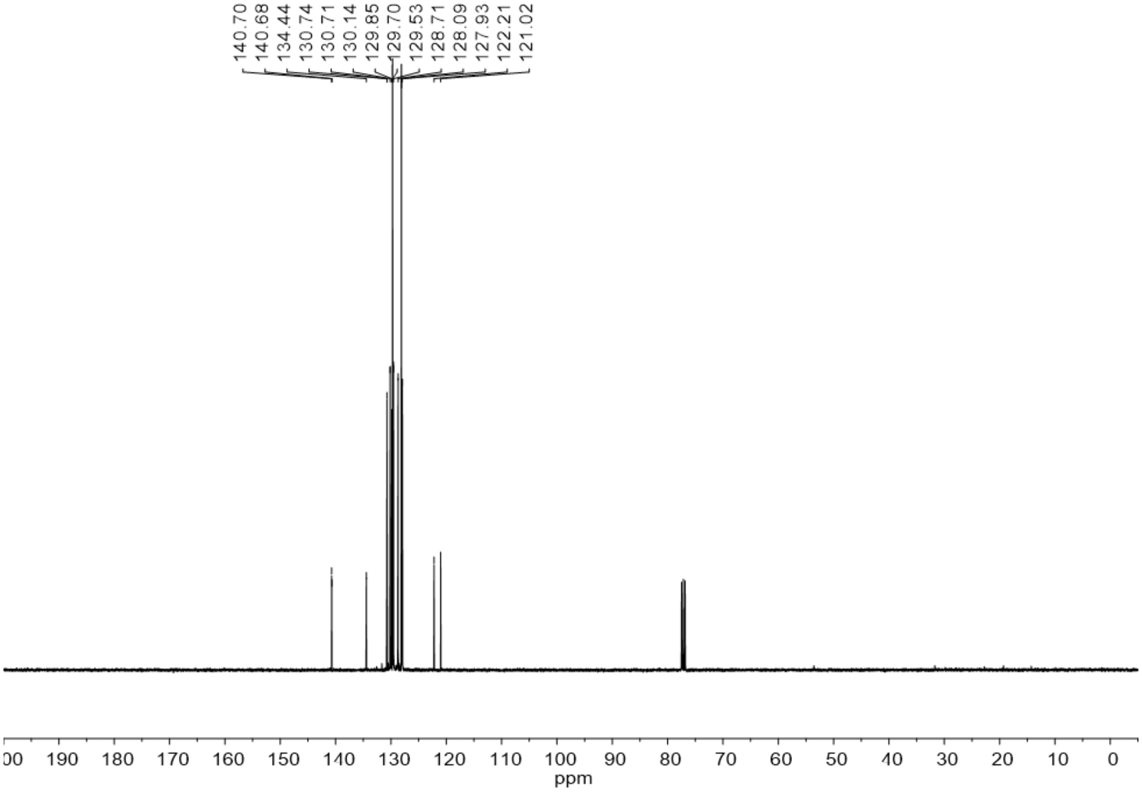


**Figure S34.** $^{13}C$ NMR of **S4** (100 MHz, $CDCl_3$, 298 K)

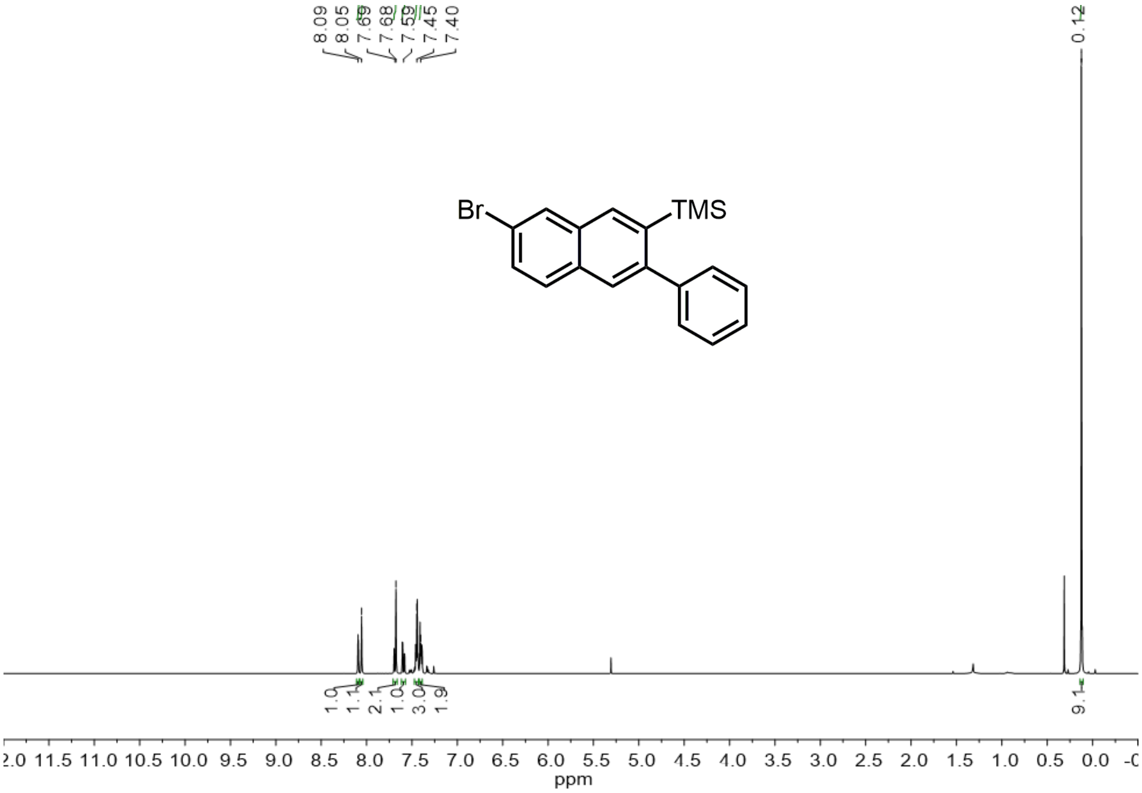


**Figure S35.** $^{1}$H NMR of **S6** (400 MHz, $CDCl_3$, 298 K)

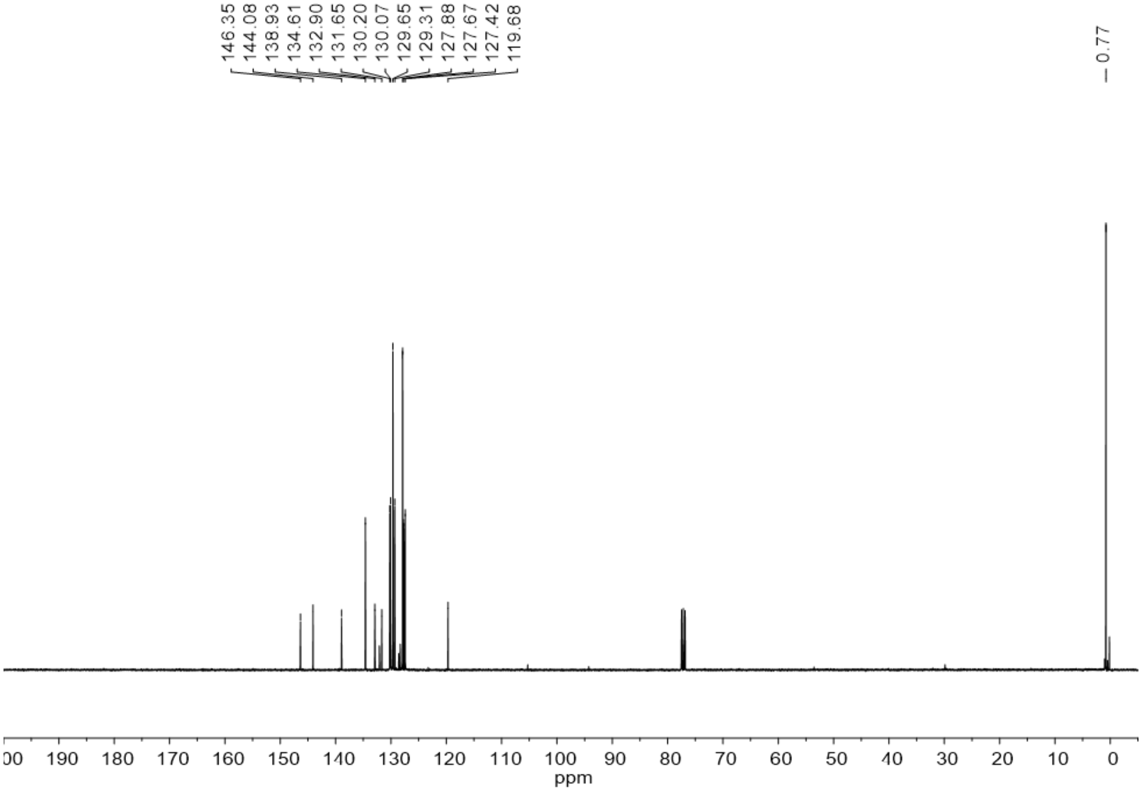


**Figure S36.** $^{13}$C NMR of **S6** (100 MHz, $CDCl_3$, 298 K)

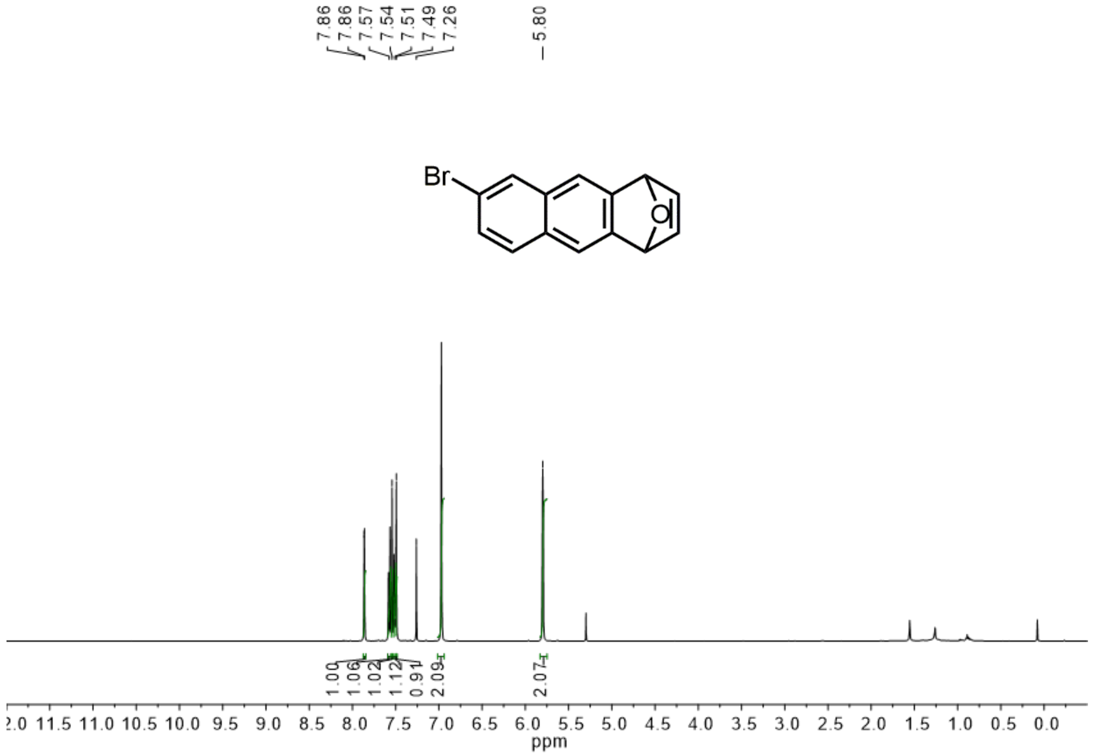


**Figure S37.** $^{1}$H NMR of **5b** (500 MHz, $CDCl_3$, 298 K)

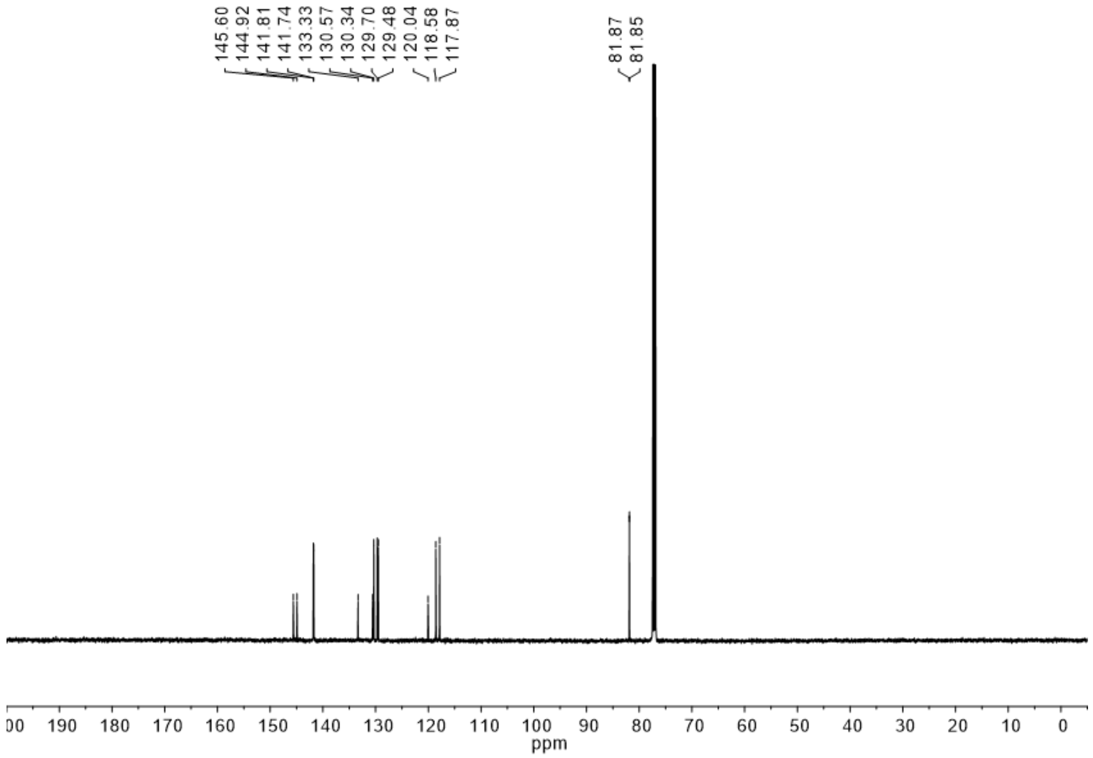


**Figure S38.** $^{13}$C NMR of **5b** (125 MHz, $CDCl_3$, 298 K)

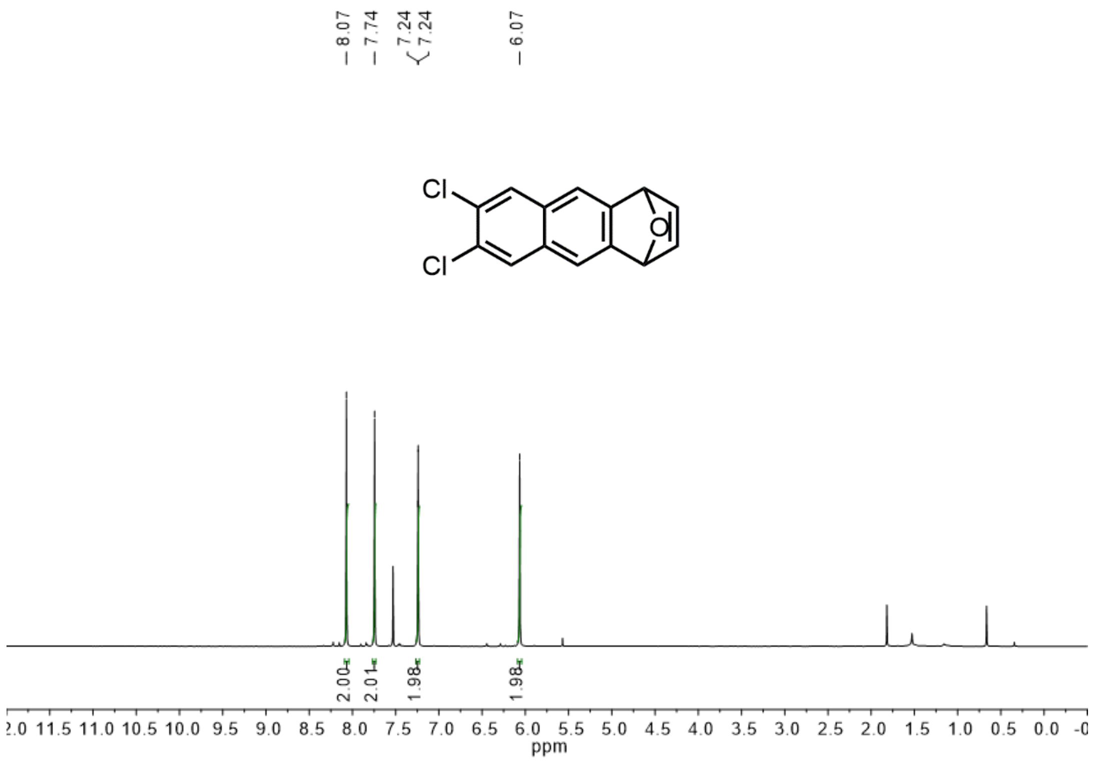


**Figure S39.** $^{1}$H NMR of **5c** (500 MHz, $CDCl_3$, 298 K)

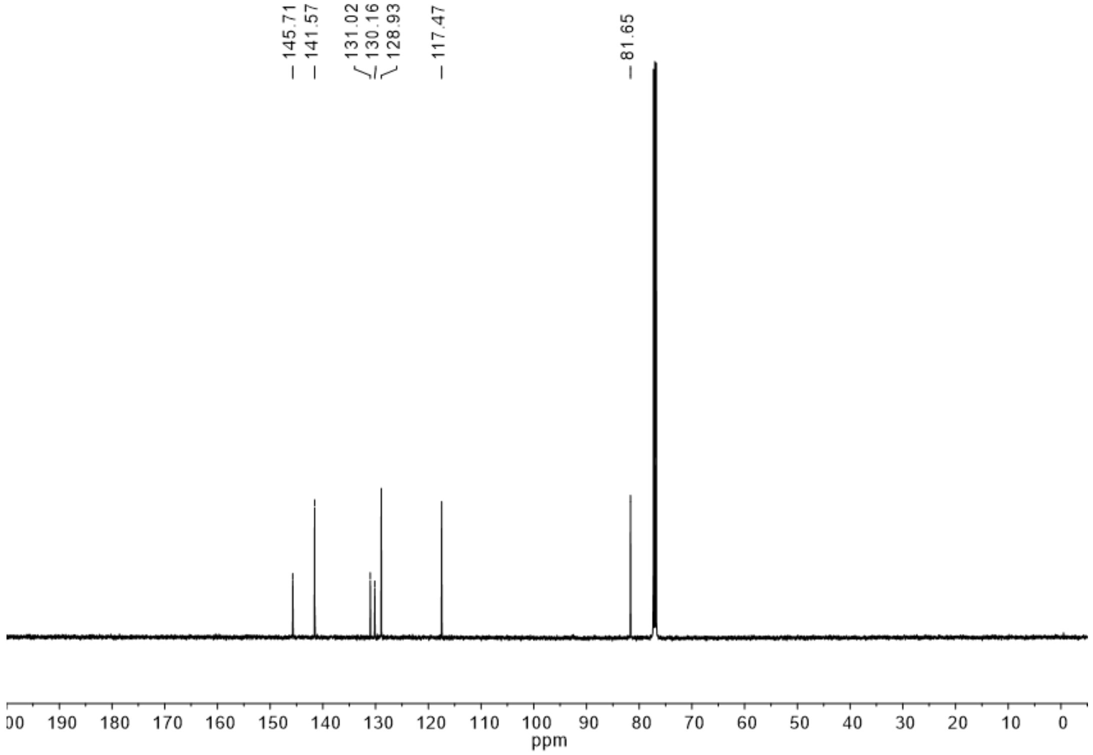


**Figure S40.** $^{13}$C NMR of **5c** (125 MHz, $CDCl_3$, 298 K)

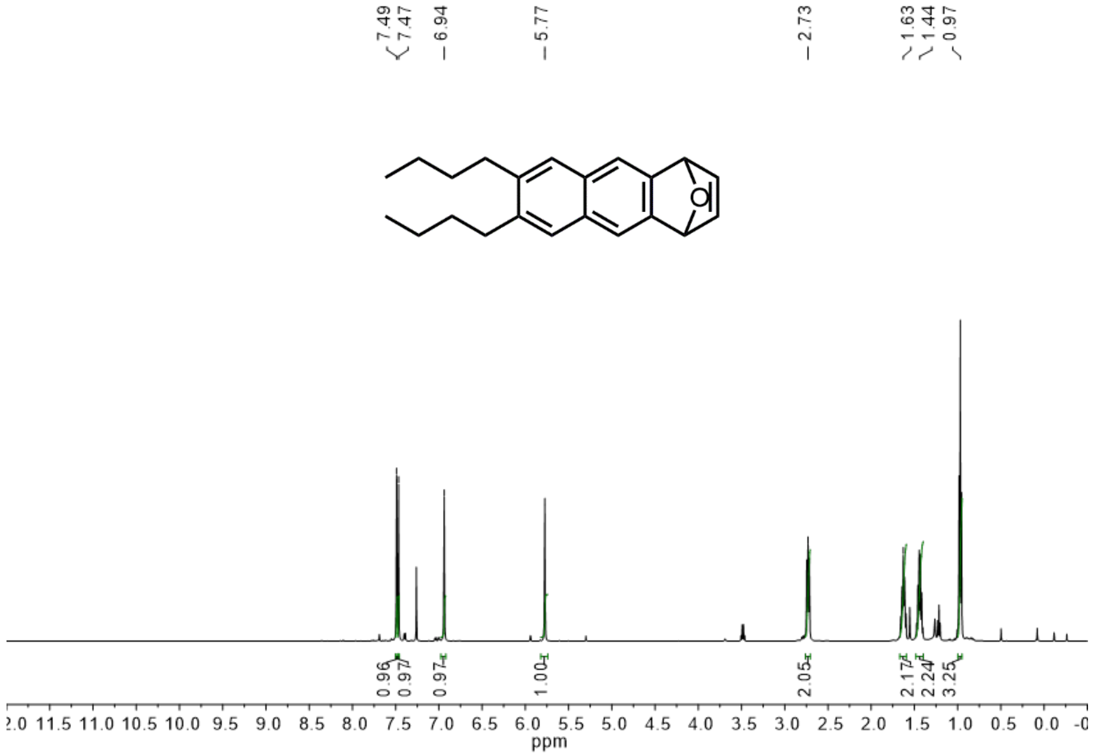


**Figure S41.** $^{1}$H NMR of **5d** (500 MHz, $CDCl_3$, 298 K)

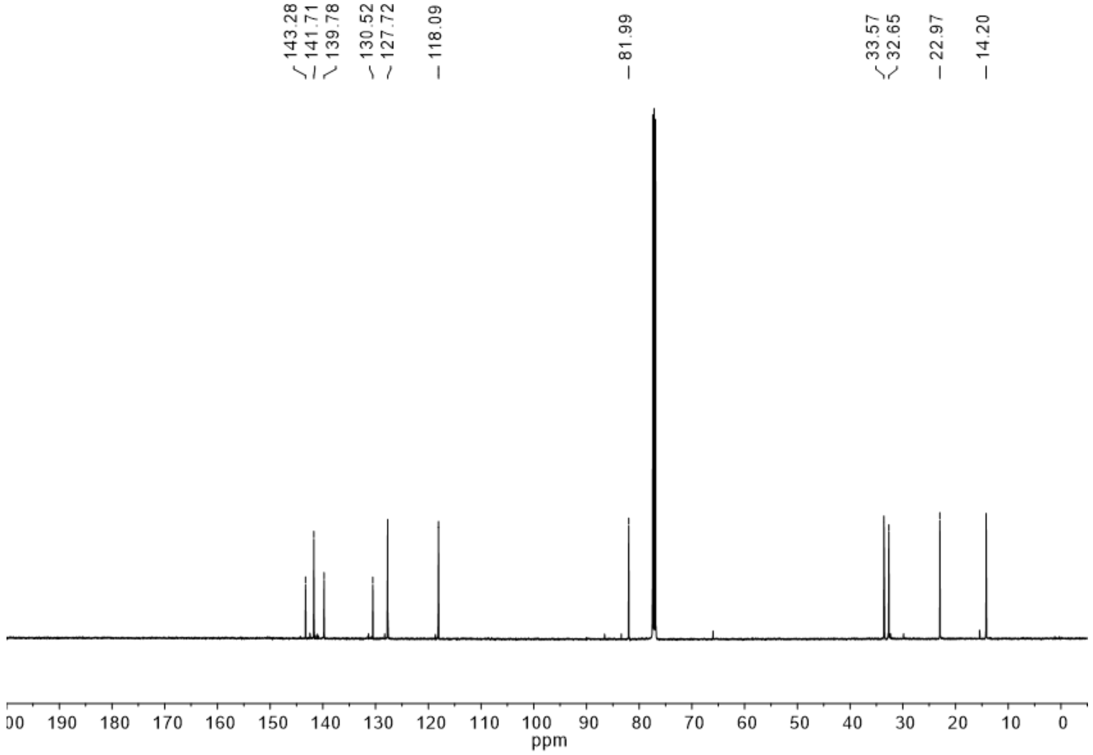


**Figure S42.** $^{13}$C NMR of **5d** (125 MHz, $CDCl_3$, 298 K)

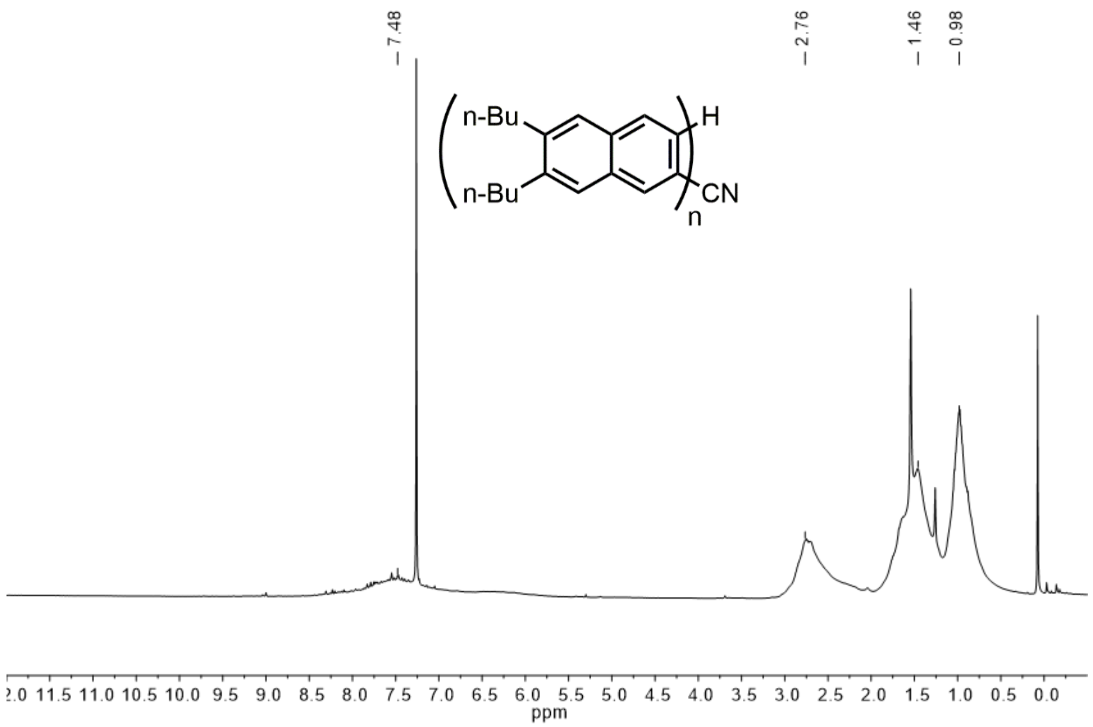


**Figure S43.** $^{1}H$ NMR of **6** (500 MHz, $CDCl_3$, 298 K)

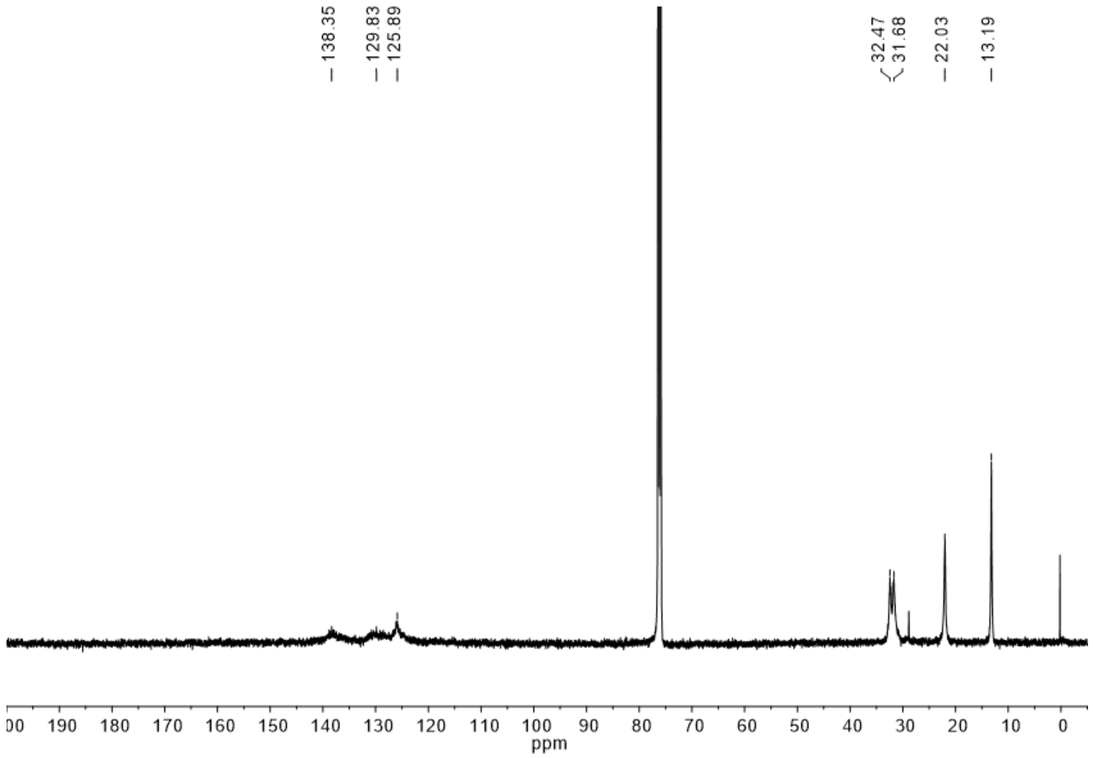


**Figure S44.** $^{13}C$ NMR of **6** (125 MHz, $CDCl_3$, 298 K)

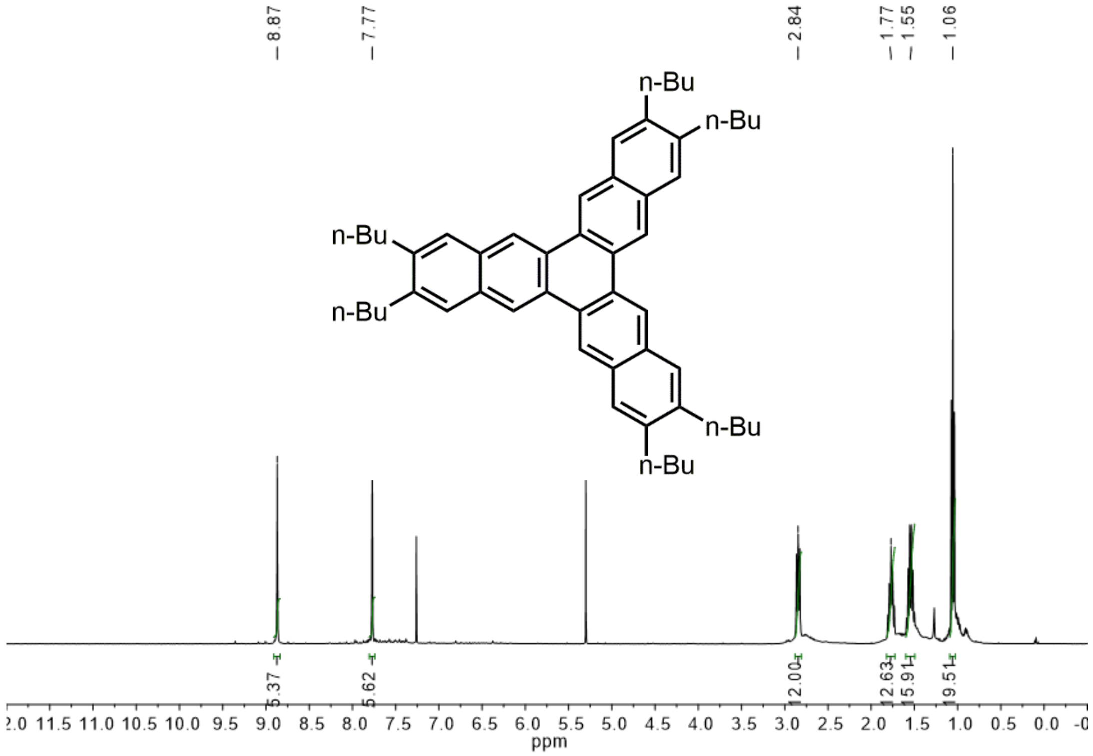


**Figure S45.** $^{1}$H NMR of **7** (500 MHz, $CDCl_3$, 298 K)

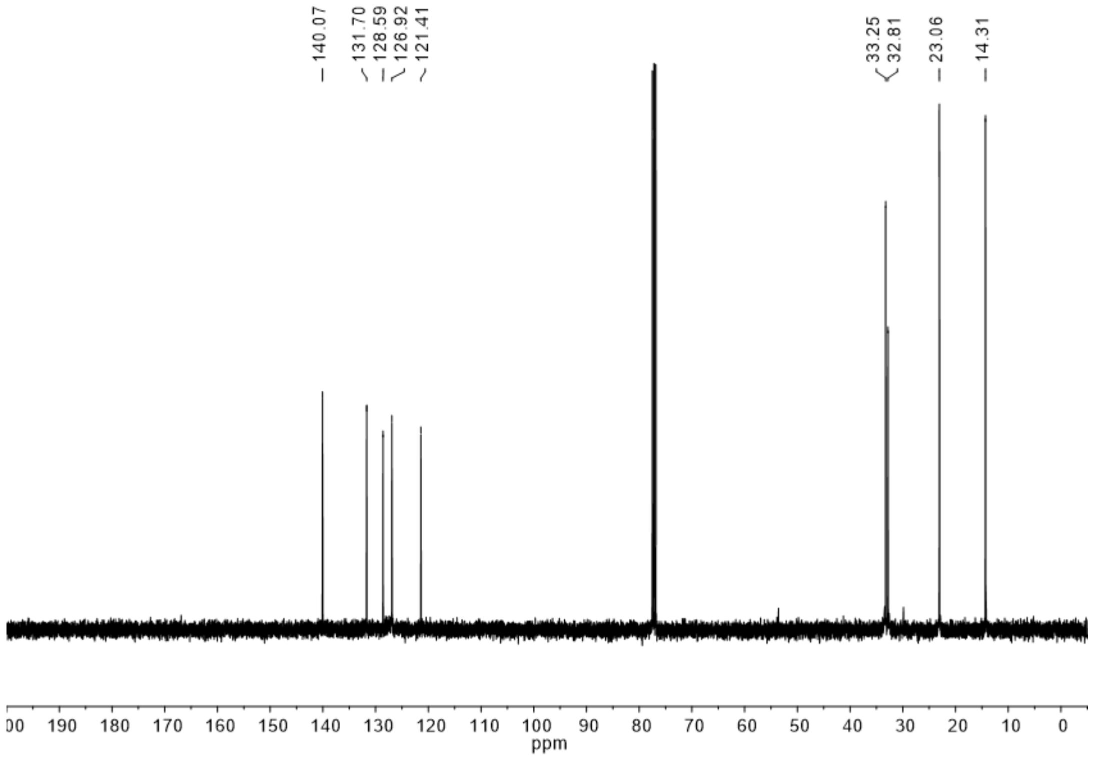


**Figure S46.** $^{13}$C NMR of **7** (125 MHz, $CDCl_3$, 298 K)

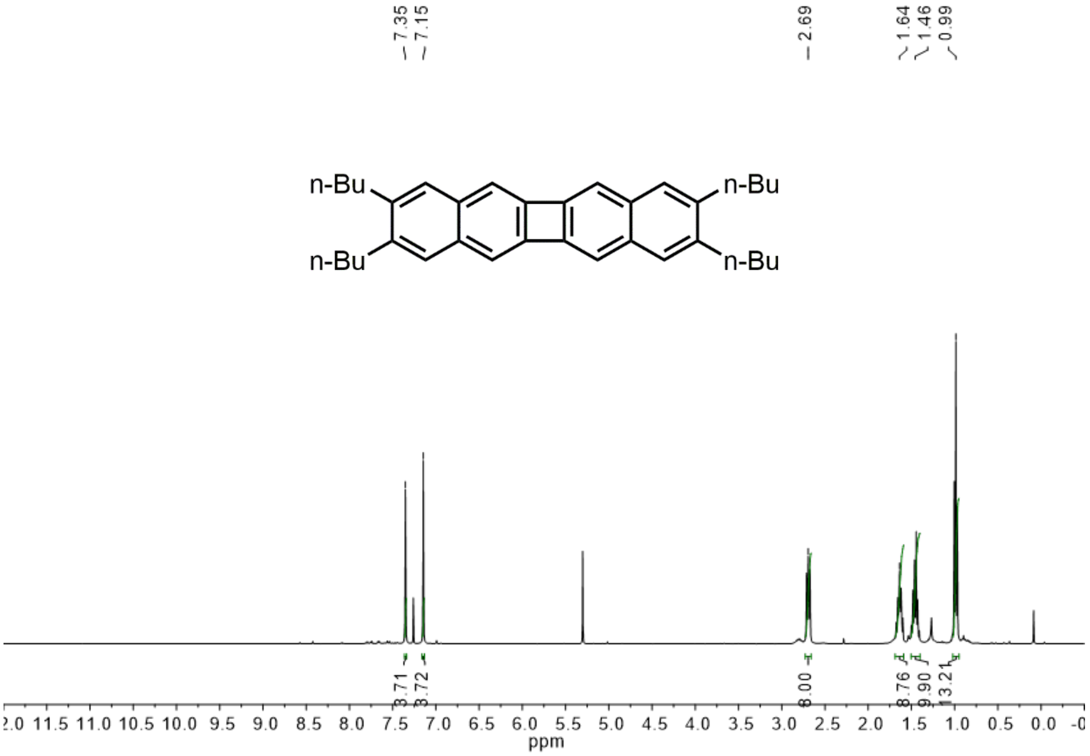


**Figure S47.** $^{1}$H NMR of **8** (500 MHz, $CDCl_3$, 298 K)

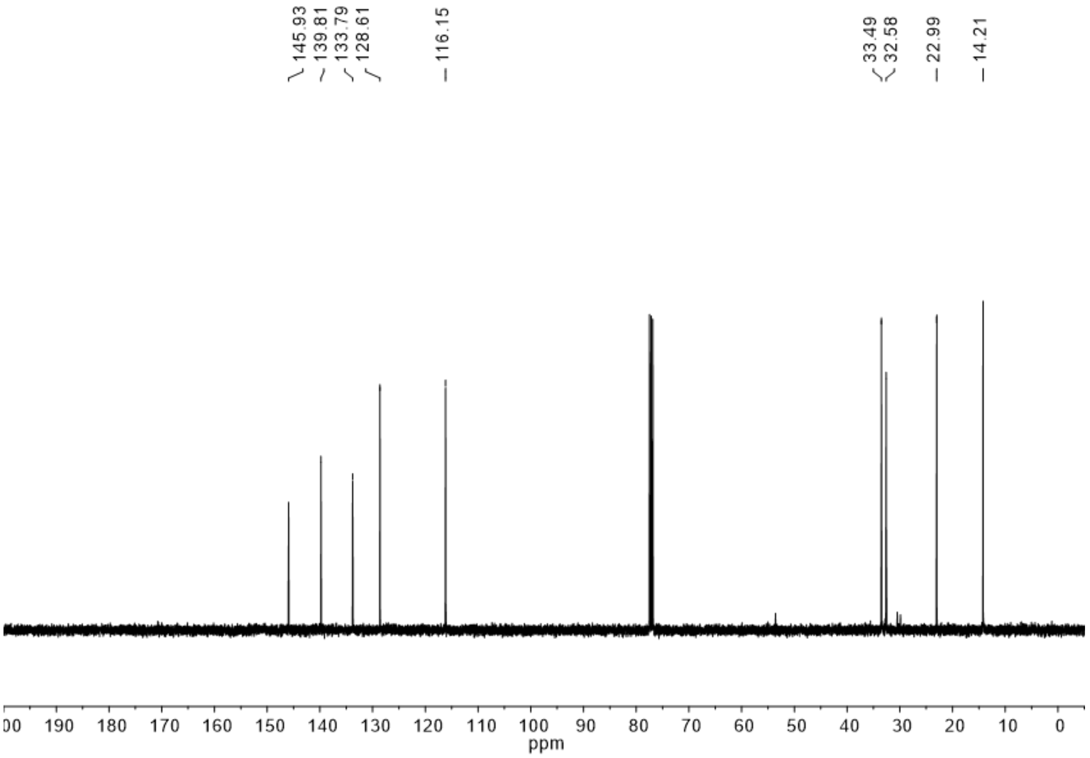


**Figure S48.** $^{13}$C NMR of **8** (125 MHz, $CDCl_3$, 298 K)

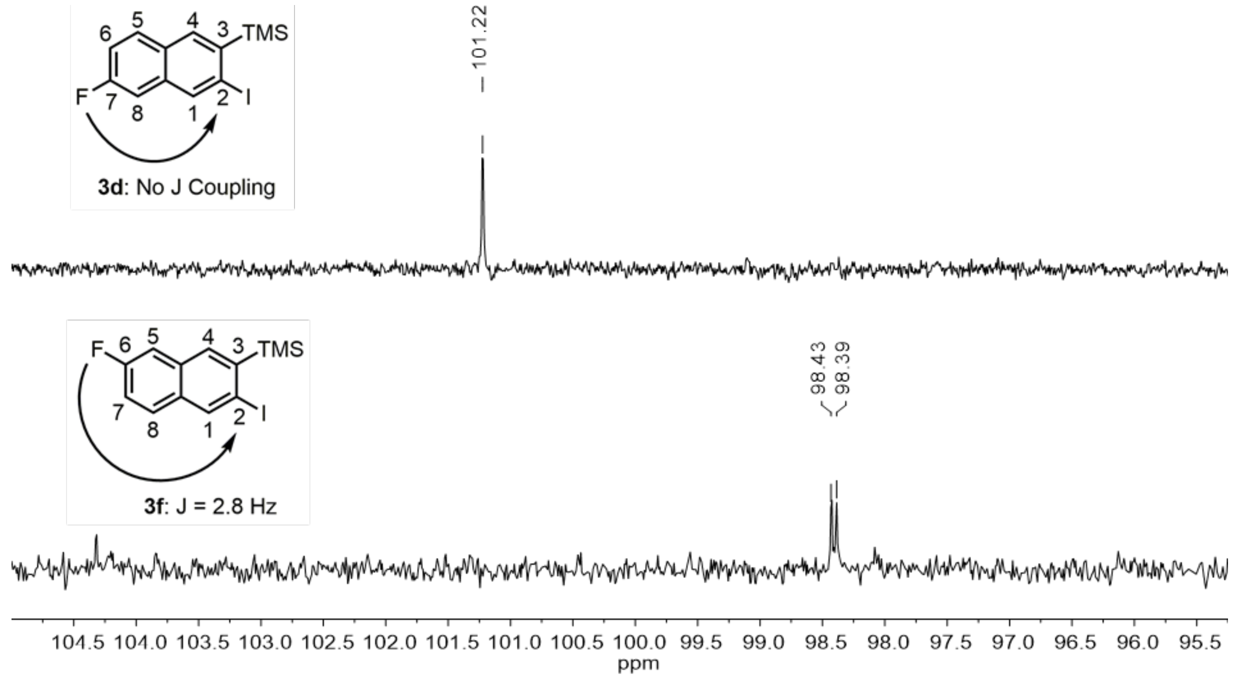


**Figure S49.** Partial $^{13}C$ NMR of **3d** and **3f** showing the long range $^{19}F$-$^{13}C$ coupling between fluorine on C7 and C6 with iodo-carbon C2. The weak J coupling present for **3f** indicates the observed regioisomer was formed.[6]

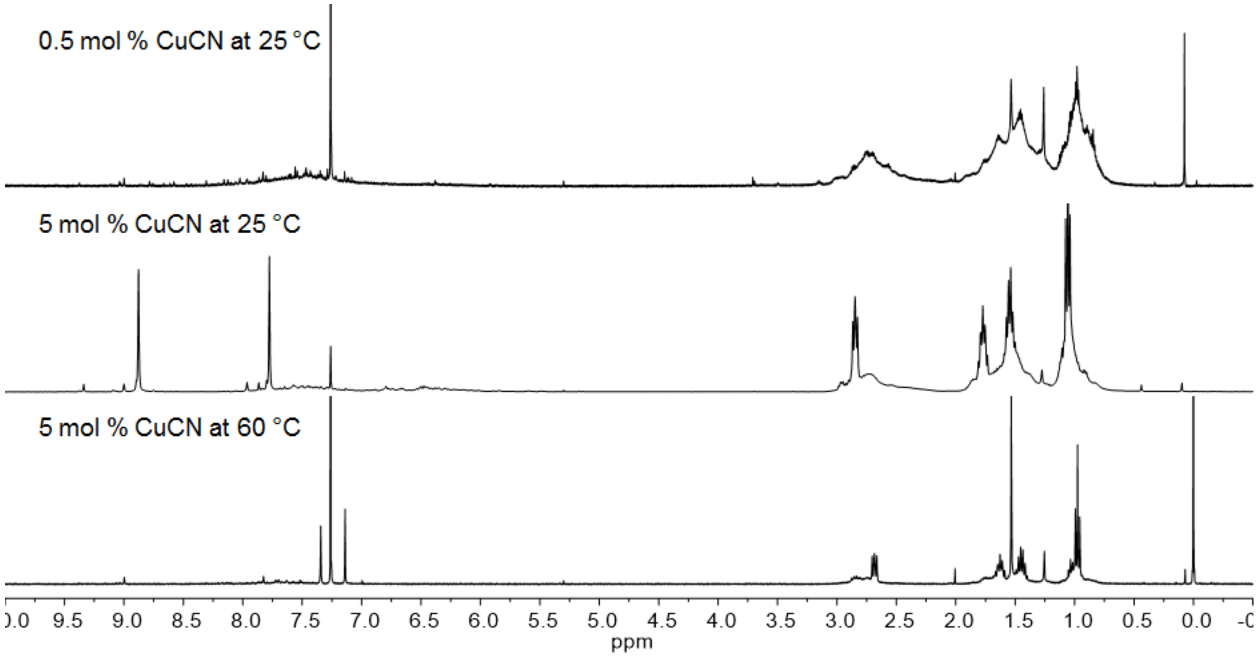


**Figure S50.** Crude $^{1}H$ NMR (400 MHz, $CDCl_3$, 298 K) of **6**, **7**, **8** after precipitation into methanol showing the selective formation of each product.

**D. 2D NMR Spectroscopy**

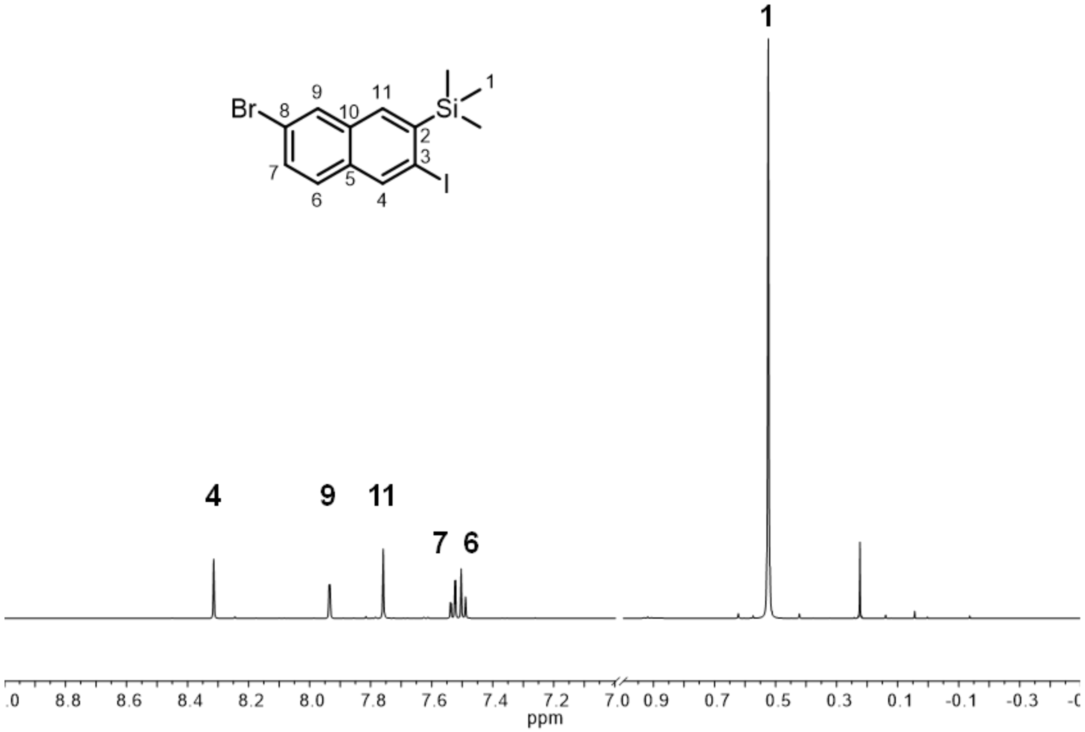


**Figure S51:** Full $^{1}H$ NMR spectrum (600 MHz, $CDCl_3$, 295 K) of **3b**, including assignments derived from HSQC and HMBC 2D NMR experiments (see below). Chemical shift range 7.0-1.0 ppm were removed to clearly show assignments.

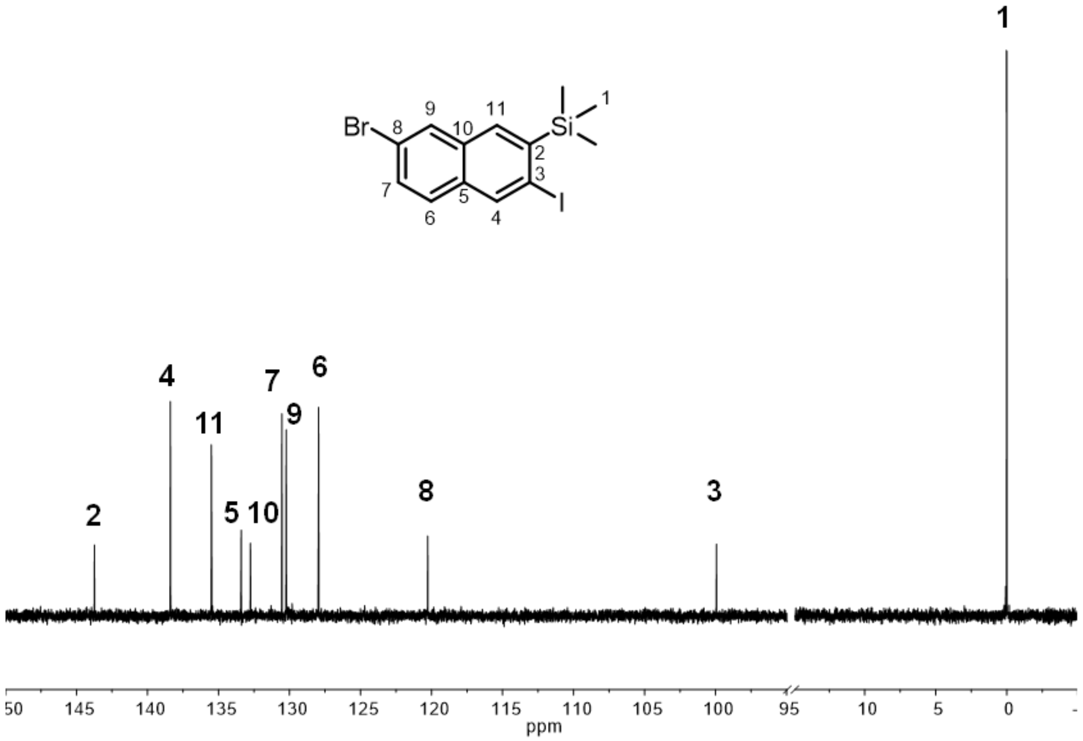


**Figure S52:** Full $^{13}C$ NMR spectrum (600 MHz, $CDCl_3$, 295 K) of **3b**, including assignments derived from HSQC and HMBC 2D NMR experiments (see below). Chemical shift range 95-15 ppm were removed to clearly show assignments.

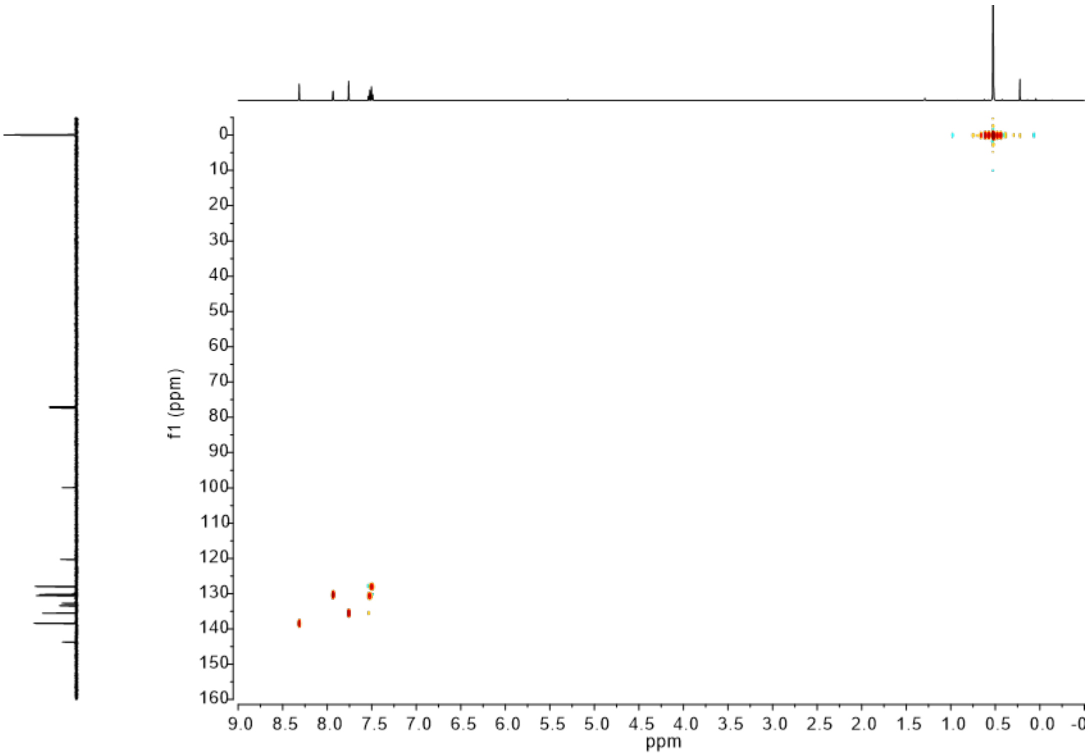


**Figure S53.** Full HSQC of **3b** (600 MHz, $CDCl_3$, 298 K)

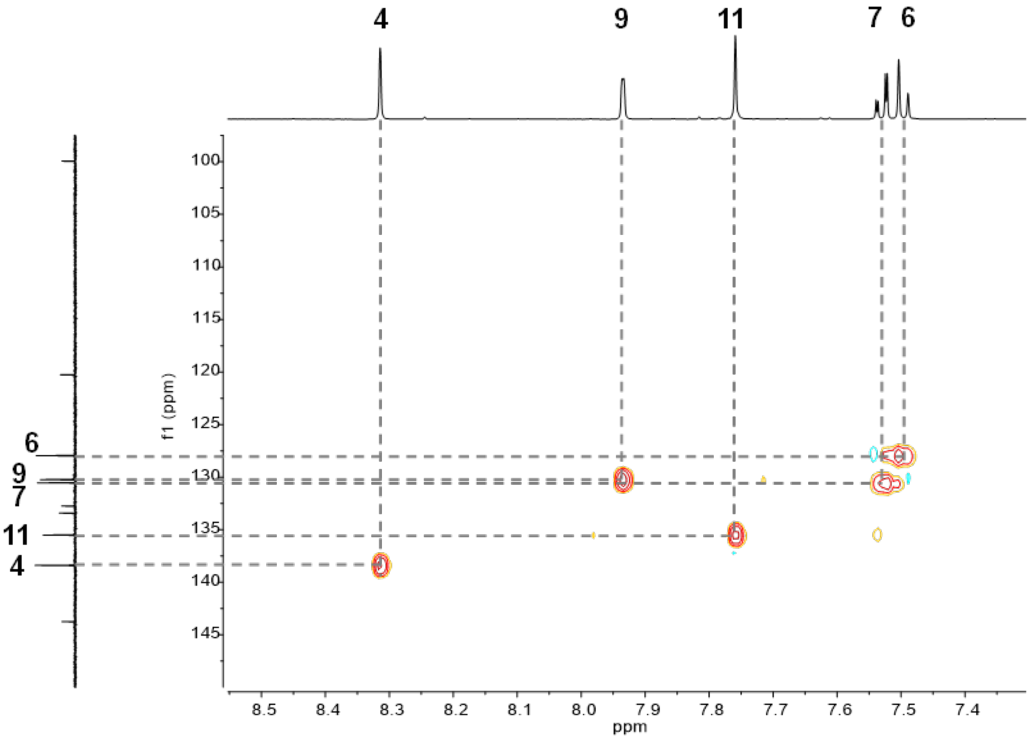


**Figure S54.** Partial HSQC of the aromatic region of **3b** (600 MHz, $CDCl_3$, 298 K)

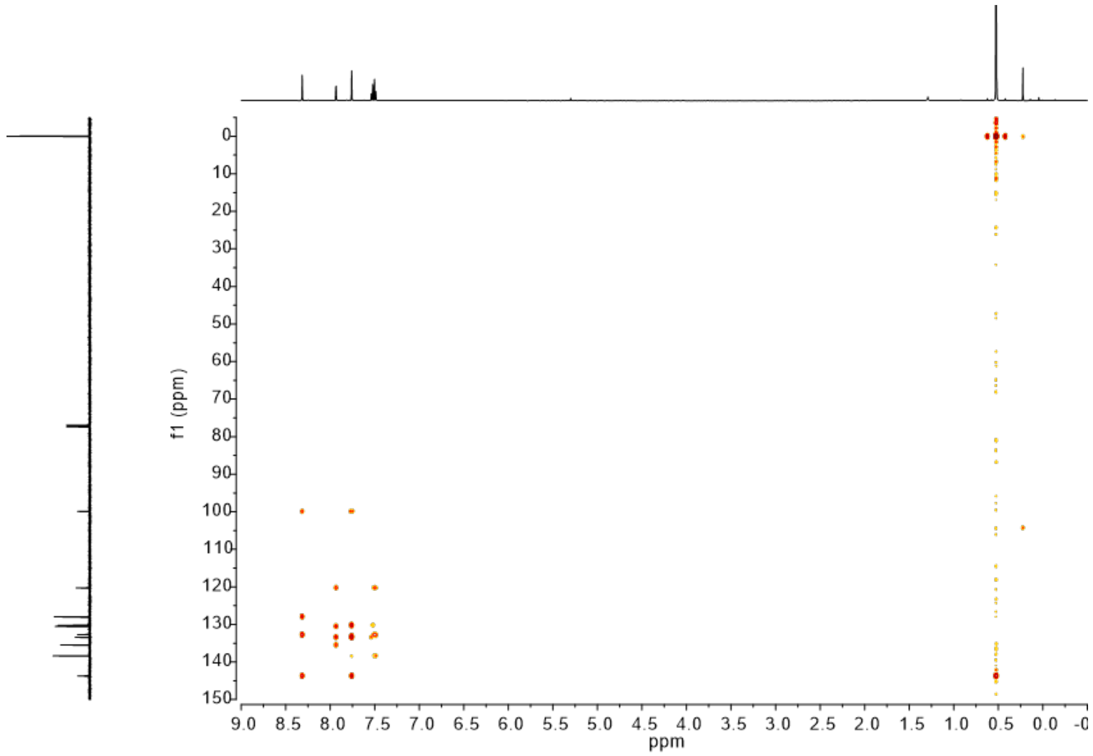


**Figure S55.** Full HMBC of **3b** (600 MHz, $CDCl_3$, 298 K)

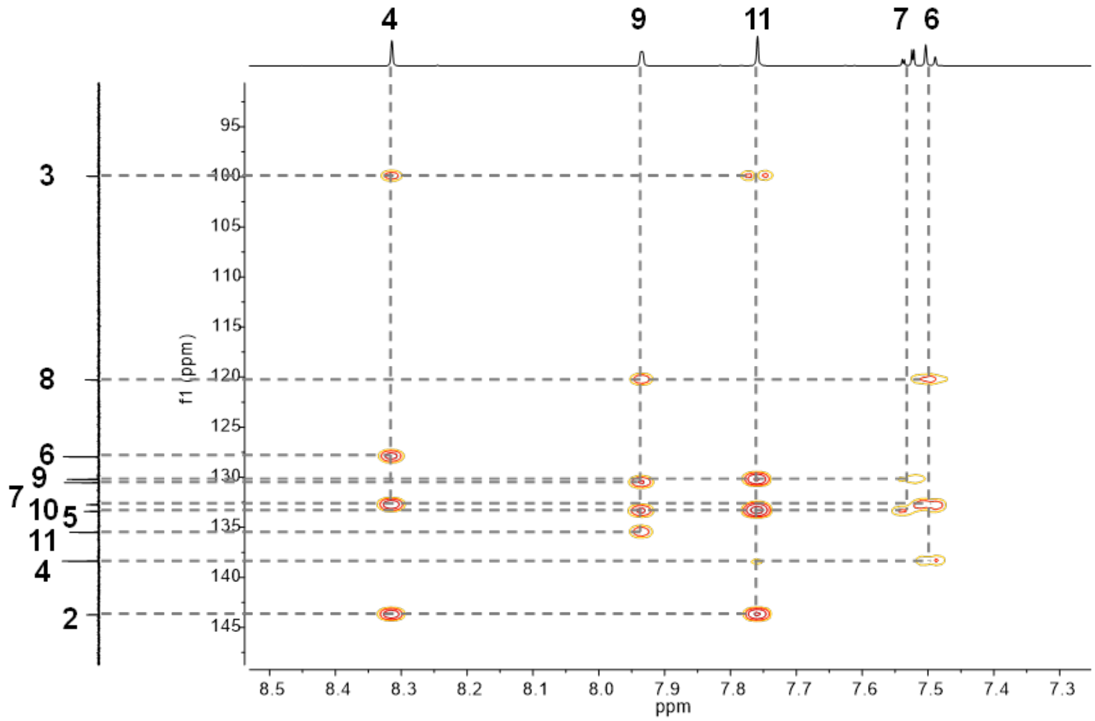


**Figure S56.** Partial HMBC of the aromatic region of **3b** (600 MHz, $CDCl_3$, 298 K)

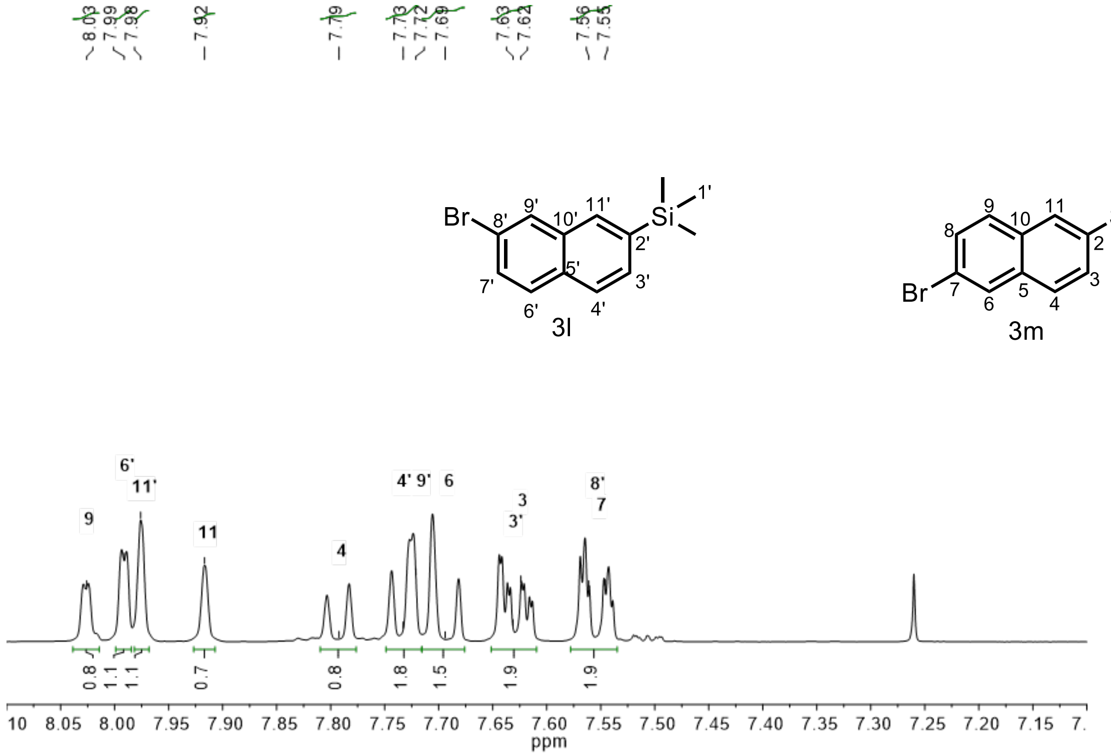


**Figure S57:** Partial $^{1}H$ NMR spectrum (400 MHz, $CDCl_3$, 295 K) of **3l** and **3m**, including assignments derived from HSQC and HMBC 2D NMR experiments (see below).

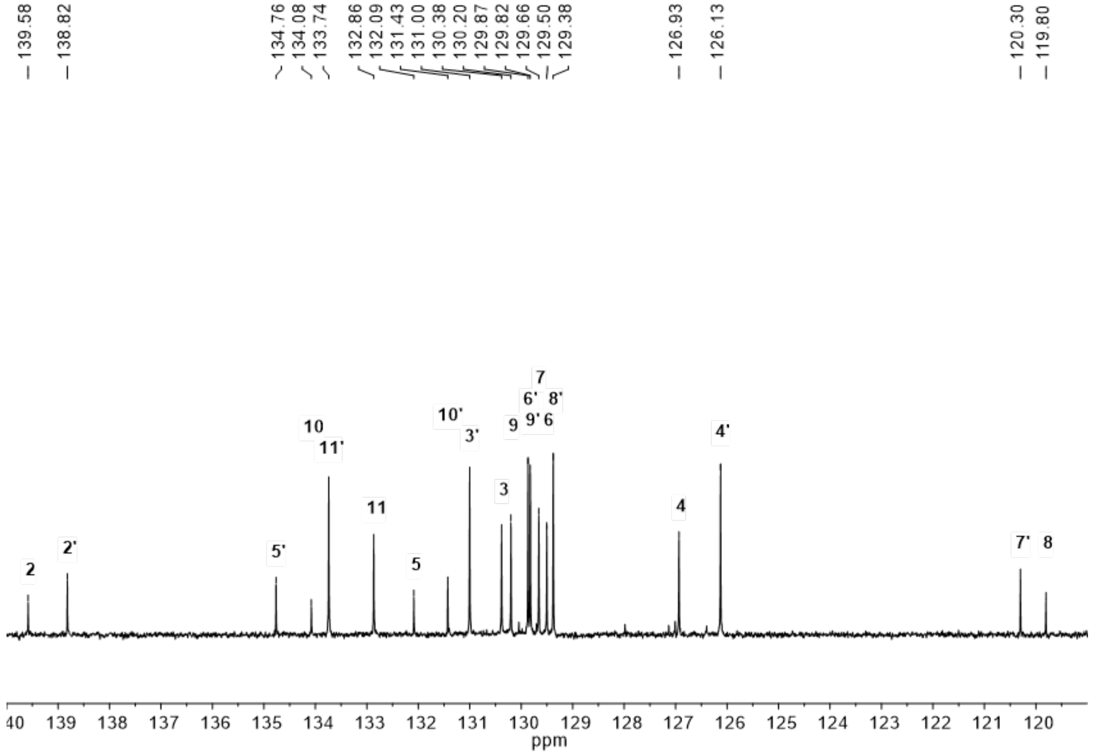


**Figure S58:** Partial $^{13}C$ NMR spectrum (100 MHz, $CDCl_3$, 295 K) of **3l** and **3m**, including assignments derived from HSQC and HMBC 2D NMR experiments (see below).

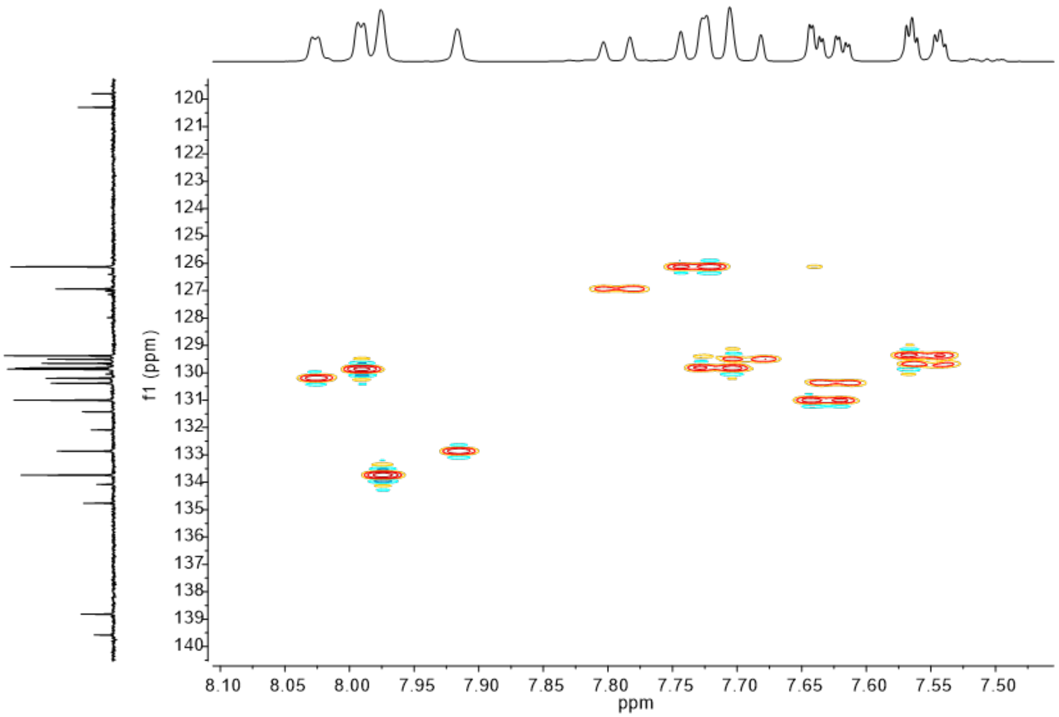


**Figure S59:** Partial band-selective HSQC spectrum of the aromatic region of **3l** and **3m** (100 MHz, $CDCl_3$, 295 K).

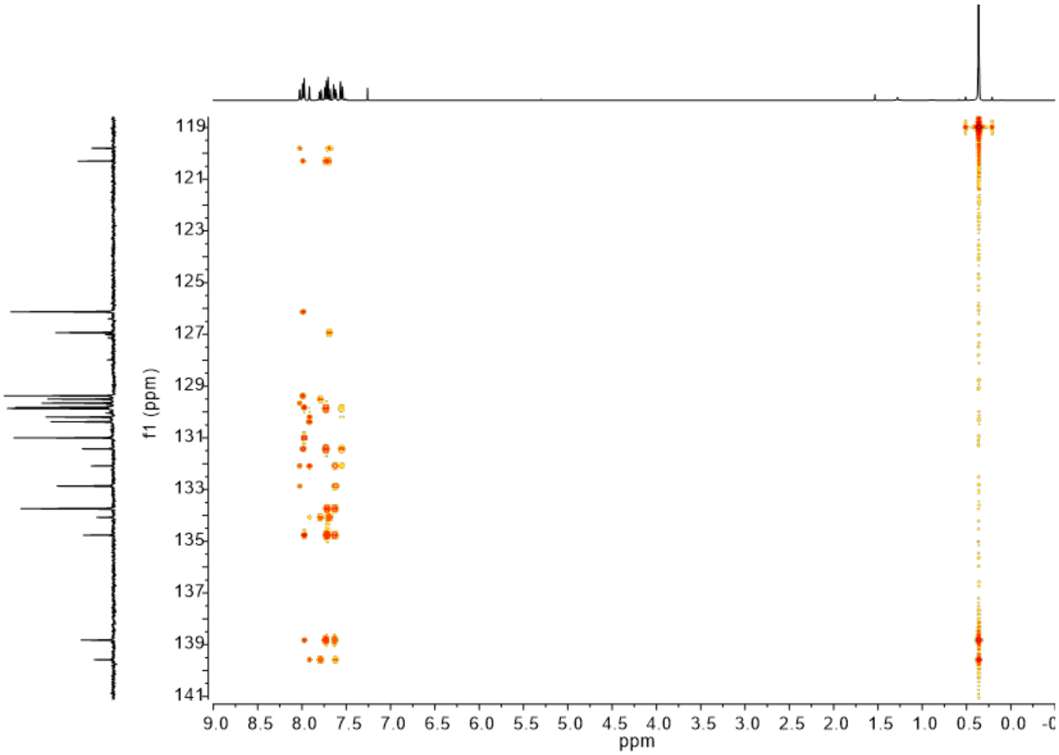


**Figure S60:** Band-selective HMBC spectrum of **3l** and **3m** (100 MHz, $CDCl_3$, 295 K).

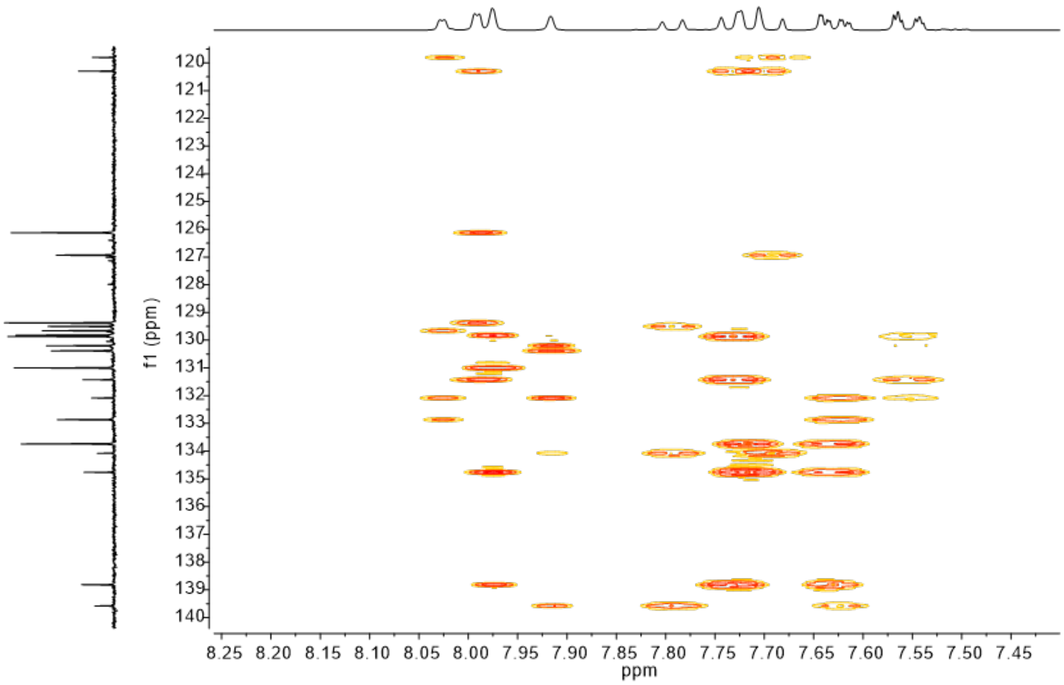


**Figure S61:** Partial band-selective HMBC spectrum of the aromatic region of **3l** and **3m** (100 MHz, $CDCl_3$, 295 K).

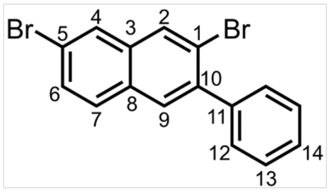


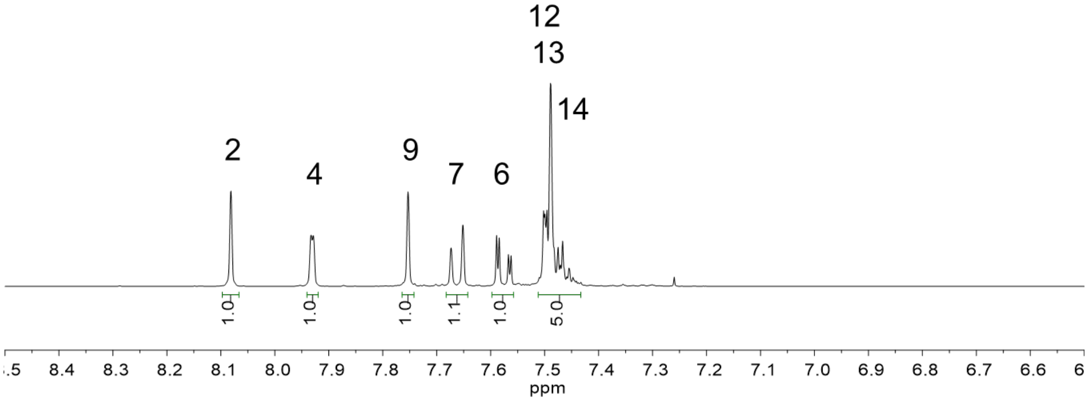


**Figure S62:** Partial $^{1}$H NMR spectrum (400 MHz, $CDCl_3$, 295 K) of **S4**, including assignments derived from HSQC and HMBC 2D NMR experiments (see below).

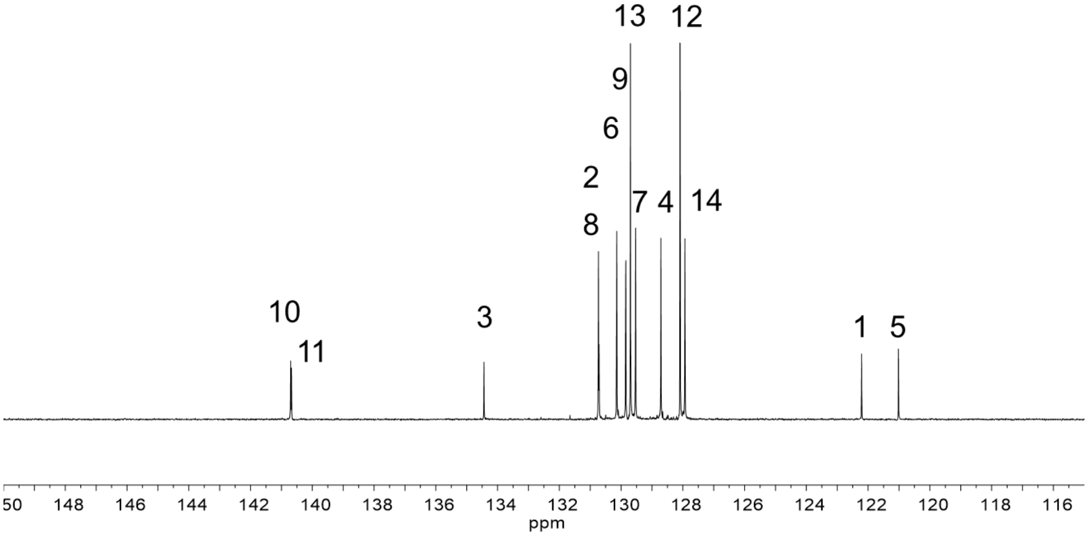


**Figure S63:** Partial $^{13}$C NMR spectrum (100 MHz, $CDCl_3$, 295 K) of **S4**, including assignments derived from HSQC and HMBC 2D NMR experiments (see below).

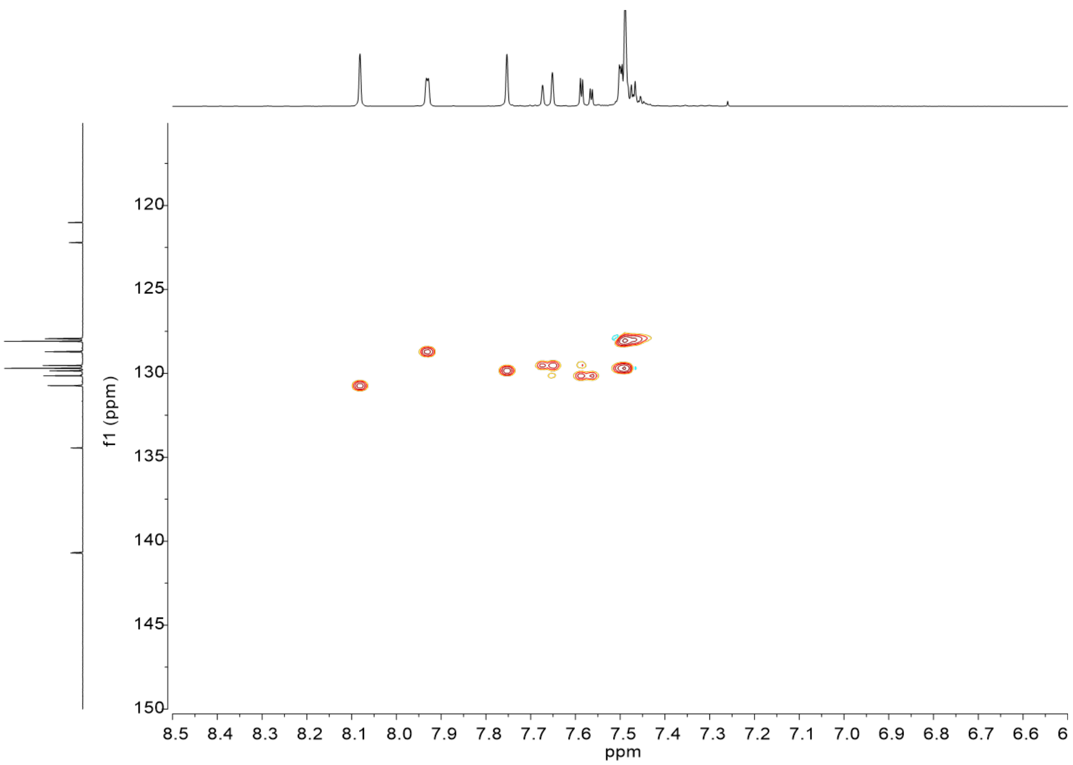


**Figure S64:** Partial band-selective HSQC spectrum of the aromatic region of **S4** (100 MHz, $CDCl_3$, 295 K).

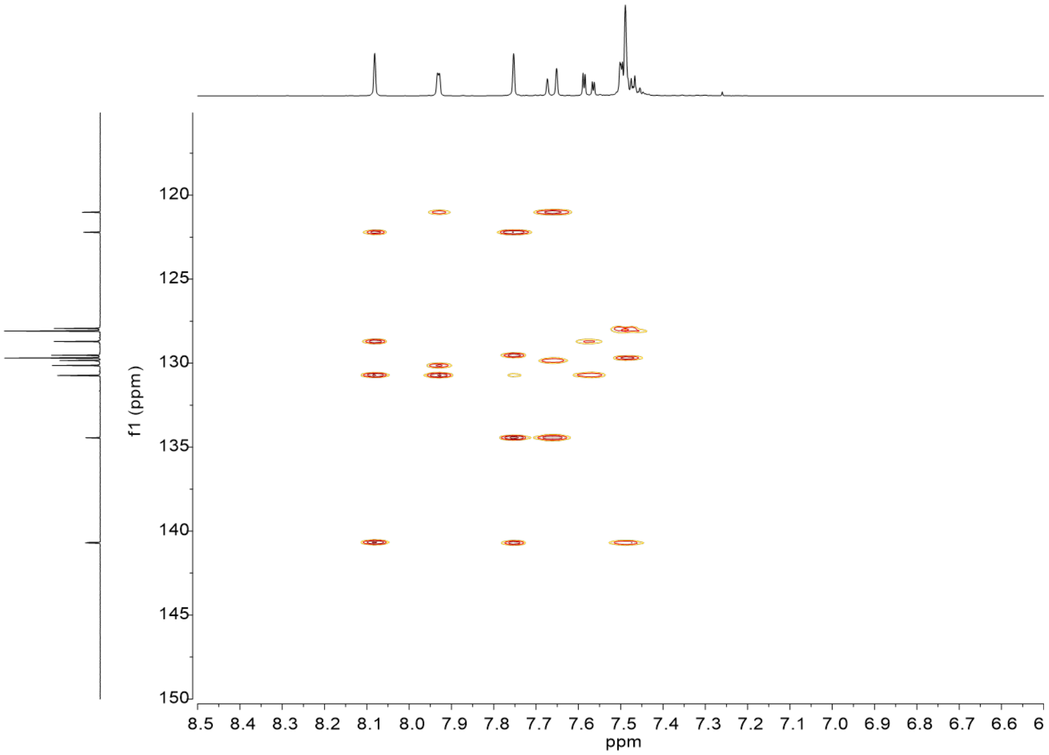


**Figure S65:** Partial band-selective HMBC spectrum of the aromatic region of **S4** (100 MHz, $CDCl_3$, 295 K).

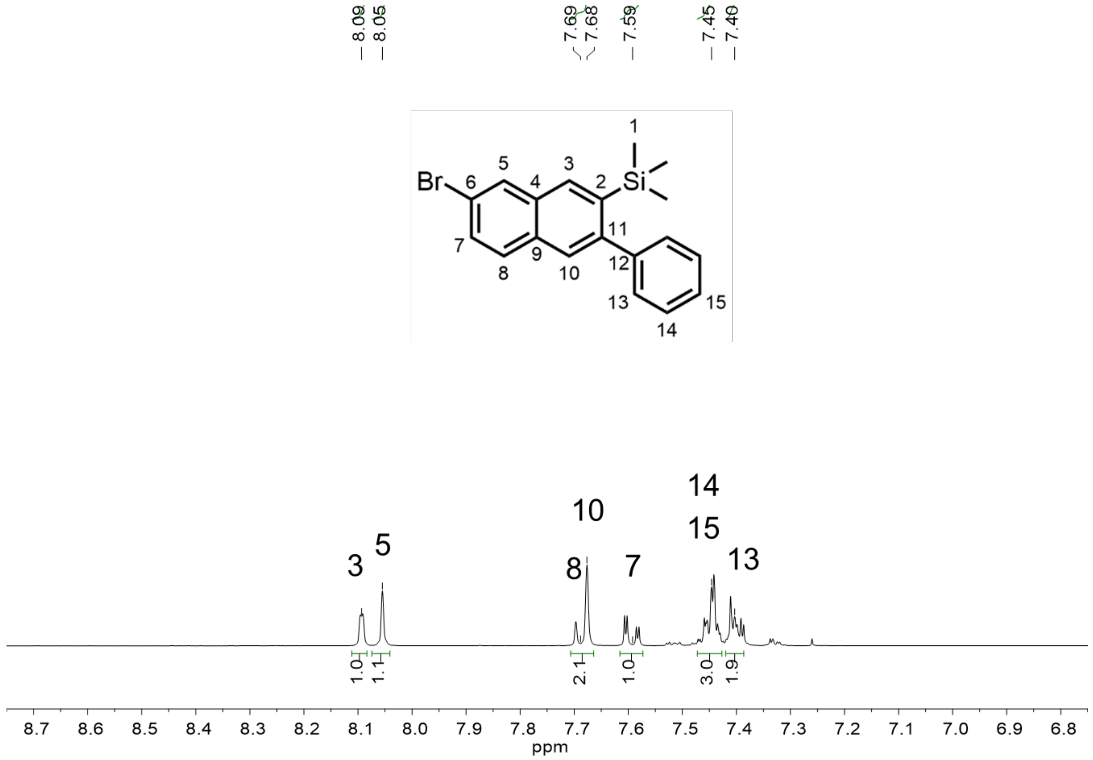


**Figure S66:** Partial $^{1}H$ NMR spectrum (400 MHz, $CDCl_3$, 295 K) of **S6** from, including assignments derived from HSQC and HMBC 2D NMR experiments (see below).

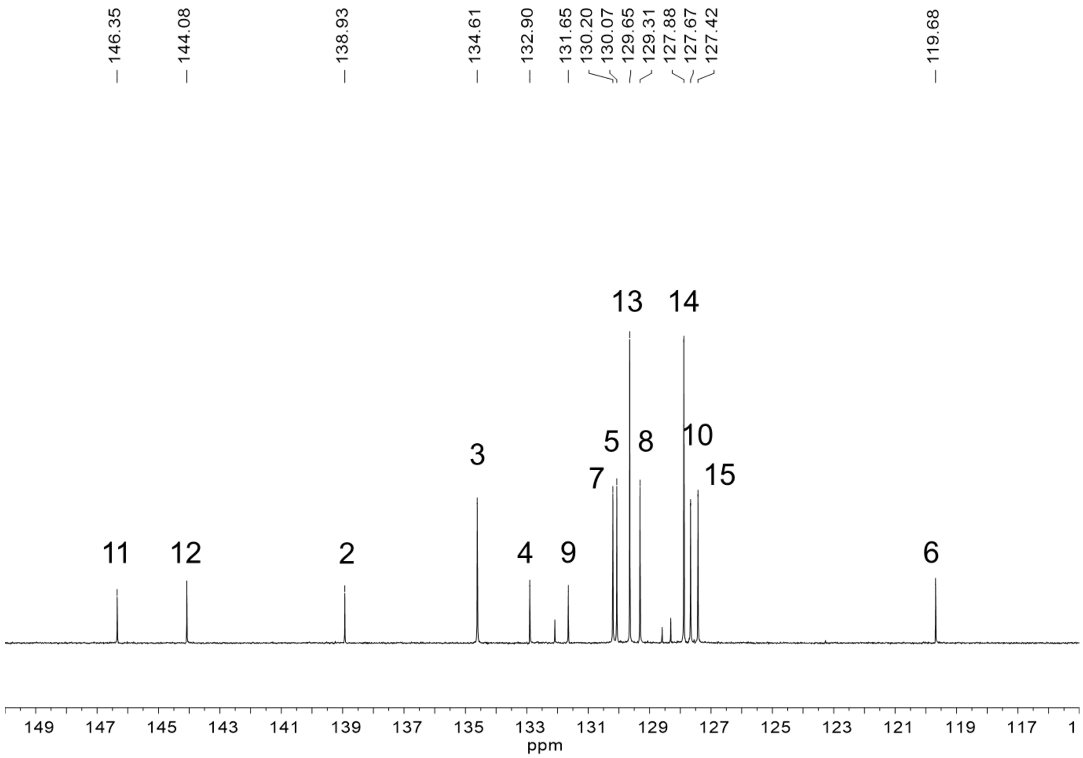


**Figure S67:** Partial $^{13}C$ NMR spectrum (100 MHz, $CDCl_3$, 295 K) of **S6**, including assignments derived from HSQC and HMBC 2D NMR experiments (see below).

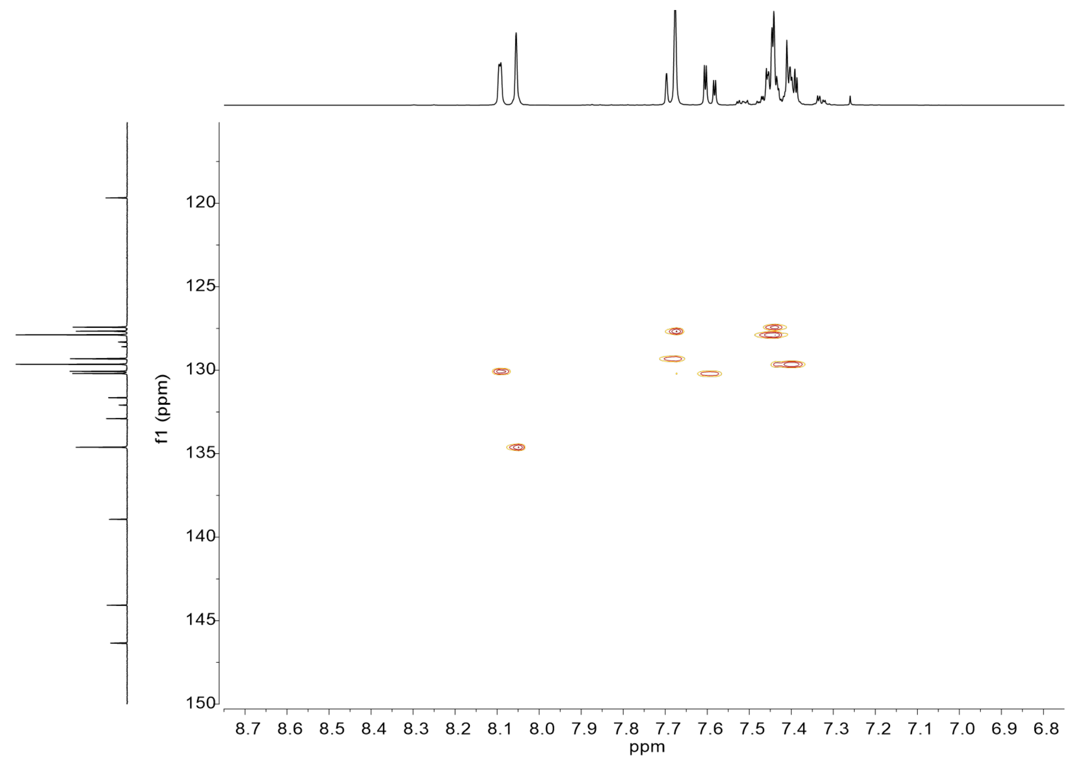


**Figure S68:** Partial band-selective HSQC spectrum of the aromatic region of **S6** (100 MHz, $CDCl_3$, 295 K).

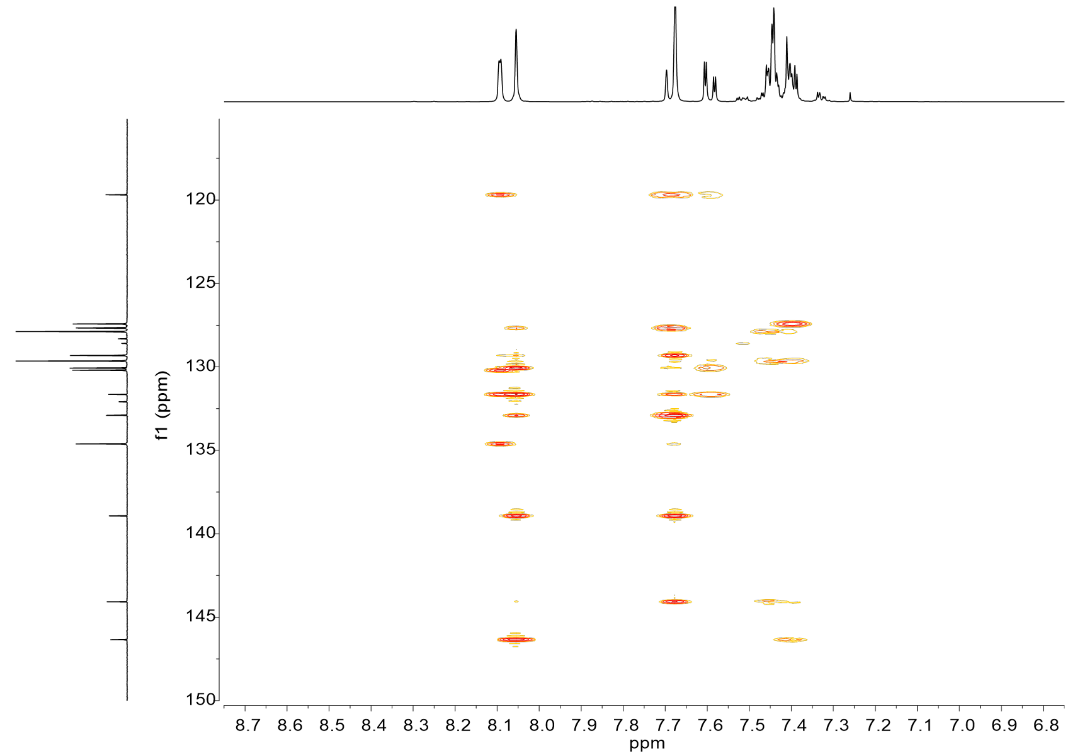


**Figure S69:** Partial band-selective HMBC spectrum of the aromatic region of **S6** (100 MHz, $CDCl_3$, 295 K).

## E. DFT Calculations

### Discussion of Proposed Reaction Mechanism Associated Regiochemical Outcomes

Mechanistic aspects of the $Cu(OTf)_2$ catalyzed benzannulation of arylhaloalkynes was reported by Dichtel and coworkers in *Chem. Eur. J.* **2015**, *21*, 18122. A comprehensive discussion of experimental and computational studies related to the mechanism and evidence for route 2 can be found therein.

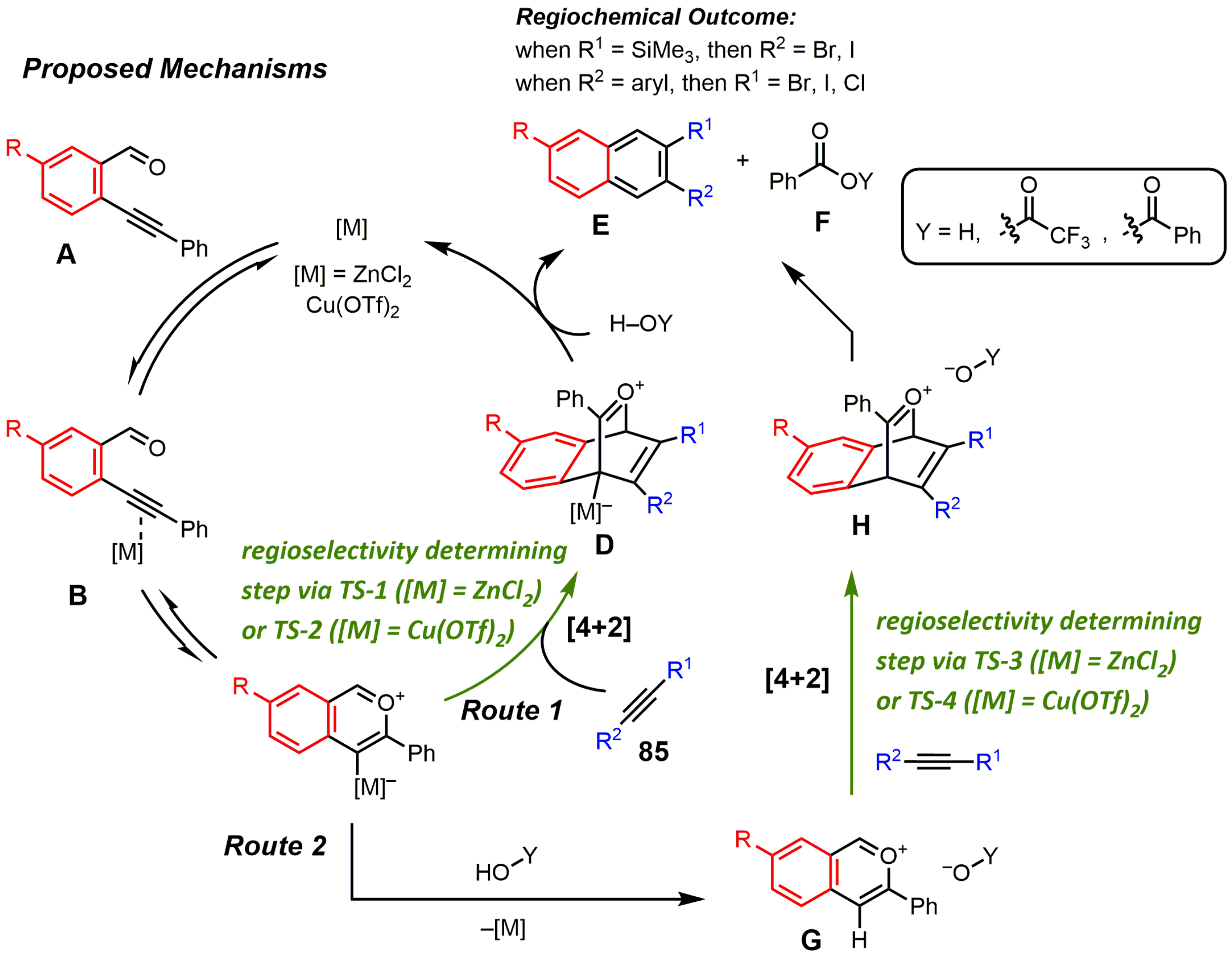


**Figure S70** Proposed mechanism based on reported mechanistic studies of the $Cu(OTf)_2$ catalyzed benzannulation of arylhaloalkynes (see reference 1 in the Supporting Information).

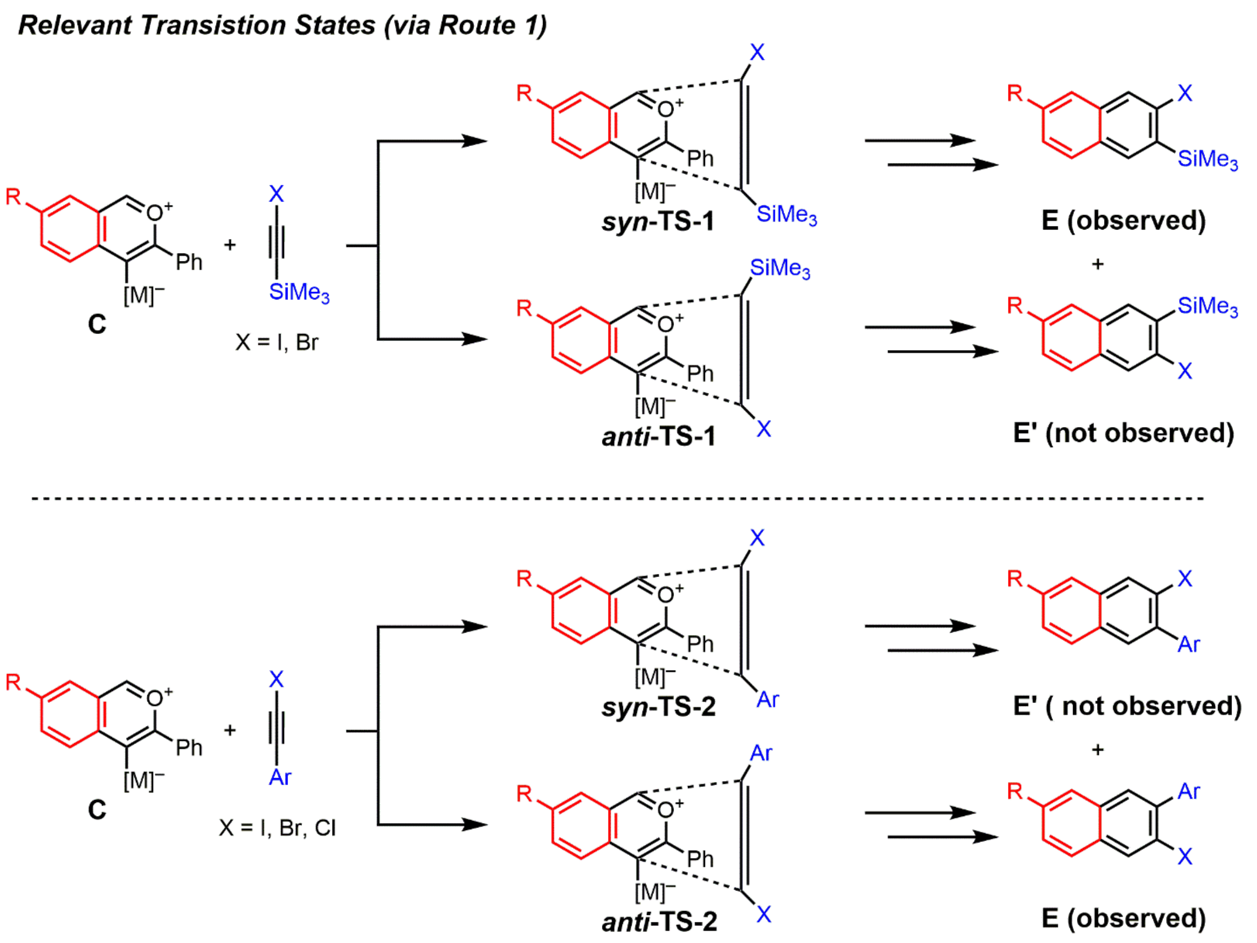


**Figure S71.** Regioselectivity determining transition state relevant to route 1 of mechanism proposed in Figure **S57**. [M] = $ZnCl_2$ or $Cu(OTf)_2$.

***Relevant Transistion States (via Route 2)***

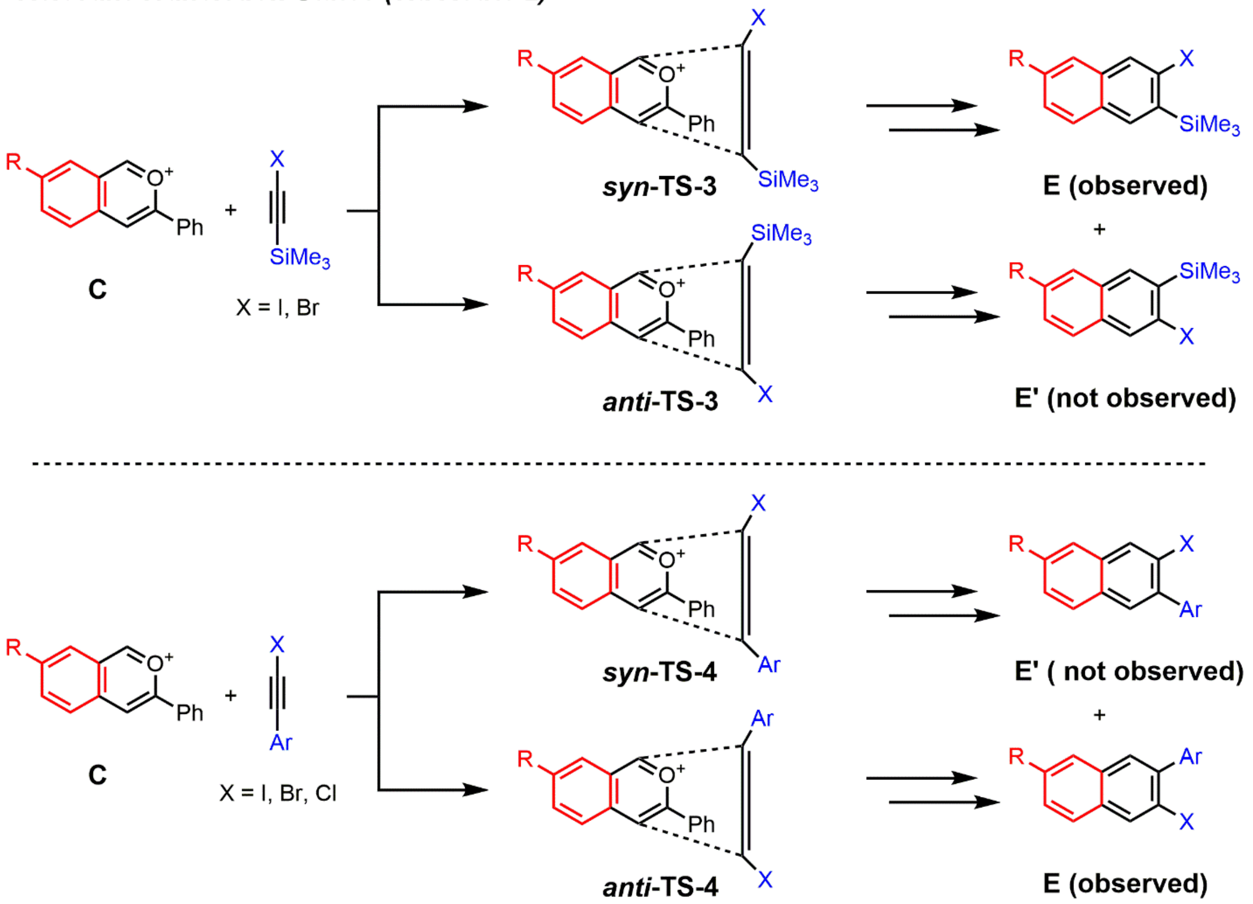


**Figure S72.** Regioselectivity determining transition state relevant to route 2 of mechanism proposed in Figure **S59**. The trifluoroacetate counter-anion has been omitted from **C**, **TS-3**, **TS-4** for computational simplification.

**Computational Method and Graphical Representation.** Calculations were performed using the Gaussian 09 program.[7] Default geometric and SCF convergence criteria were used. Stationary points were characterized by the presence of all positive eigenvalues of the Hessian for minima or a single negative eigenvalue of the Hessian for transition states. All DFT methods were used as their default implementations in Gaussian 09. All electronic energies reported are corrected energies with regards to zero point energy (ZPE) corrections. Cartesian coordinates for **TS-2** and **TS-4** were previously reported.[1] All molecular structures were rendered in CYLView.[8] All CYLView generated images have the following color-coding for labeling elements: C = silver, H = white, O = light red, Zn = blue, Cl = dark green, F = light green, Si = beige, S = yellow, Br = dark red, Cu = bronze.

**Table S1.** Comparison of regioisomeric transition state energies calculated using B3LYP/ 6-31G(d) in the gas phase.[a]

| **Substrate** | **Metal** | ***Regioisomer*** | $E_{ZPE}$ **[kcal/mol]** | $G_{298K}$ **[kcal/mol]** | **Conclusion** |
|---|---|---|---|---|---|
| TMS-CC-Br | Zn | *syn*-TS-Zn | +1.98 | +2.73 | *anti*-isomer preferred |
| | Zn | *anti*-TS-Zn | +0.00 (def.) | +0.00 (def.) | |
| | none (H) | *syn*-TS-H | +2.35 | +2.70 | *anti*-isomer preferred |
| | none (H) | *anti*-TS-H | +0.00 (def.) | +0.00 (def.) | |
| TMS-CC-H | Zn | *syn*-TS-Zn | +2.83 | +3.29 | *anti*-isomer preferred |
| | Zn | *anti*-TS-Zn | +0.00 (def.) | +0.00 (def.) | |
| | none (H) | *syn*-TS-H | –1.83 | –1.52 | *syn*-isomer preferred |
| | none (H) | *anti*-TS-H | +0.00 (def.) | +0.00 (def.) | |
| Ph-CC-Br | Cu | *syn*-TS-Cu | –5.60 | –5.69 | *syn*-isomer preferred |
| | Cu | *anti*-TS-Cu | +0.00 (def.) | +0.00 (def.) | |
| | none (H) | *syn*-TS-H | –10.40 | –10.88 | *syn*-isomer preferred |
| | none (H) | *anti*-TS-H | +0.00 (def.) | +0.00 (def.) | |

[a] $E_{ZPE}$ is the zero-point energy corrected electronic energy, and $G_{298K}$ is the Gibbs free energy at 298 K. For ease of comparison, the reference energy for each pairwise regioisomeric transition state comparison uses the *anti*-regioisomer as the reference energy (defined to be 0.00 kcal/mol).

**Discussion of Computational Results**

Based on the mechanistic scenario presented in Figures S64 & S65, a low-level DFT model of the proposed transition states leading to either the *syn*- or *anti*-regioisomer was developed. This model correctly predicts the reversal in regioselectivity observed experimentally between silylhaloalkynes and arylhaloalkynes (Table S1 & Figures S1–S2). The B3LYP/6-31G(d) calculated ZPE-corrected electronic energies of regioisomeric transition states predict a 2.0 kcal/mol preference for the *anti*-regioisomer in the case of silylhaloalkyne **2b** compared to 5.6 kcal/mol in favor of the *syn*-regioisomer for **phenylbromoalkyne**. An alternate mechanism in which the metal is not bound to the benzopyrylium intermediate is also plausible and transition state energies of those structures also support a reversal of regioselectivity when comparing silylhaloalkyne **2b** and **phenylbromalkyne** substrates.

Extension of this computational model towards describing the benzannulation of **trimethylsilylalkyne** results in the prediction that the pathway involving C-C bond formation with the Zn-bound benzopyrylium favors the transition state leading to the *anti*-isomer, while if C-C bond formation occurs with the metal-free benzopyrylium intermediate (i.e., protonation of the Zn-bound benzopyrylium precedes reactivity with the **trimethylsilylalkyne**) then the transition state leading to the *syn*-isomer is preferred. Experimentally, the reaction provides approximately equal amounts of the *syn*- and *anti*-isomer **3l** and **3m**, respectively, when using benzaldehyde **1b**. Two plausible explanations are:

(1) Both reaction pathways described above (***Route 1*** and ***Route 2*** in Figure S64) are operational and the reaction pathway partitioning is a result of the concentration and pKa of the protic source in the reaction mixture (e.g., adventitious water, reactor surface bound proton). This explanation would not be surprising since previous mechanistic study of the $Cu(OTf)_2$ catalyzed benzannulation of arylhaloalkynes found the amount/concentration and type of Brønsted acid used could affect the regioselectivity outcome of this reaction (see *Chem. Eur. J.* **2015**, *21*, 18122).

(2) The computational model could benefit further refinement such as using a solvation model, the use a dispersion corrected functional, the use of a larger basis set, the use of diffuse functions, and the inclusion of the counter-anion in the metal free transition states. All of these refinements could improve the model, but are currently outside the scope of this study since they would require significant additional computational time to evaluate whether they had a positive, negative or lack of an effect towards refining the model.

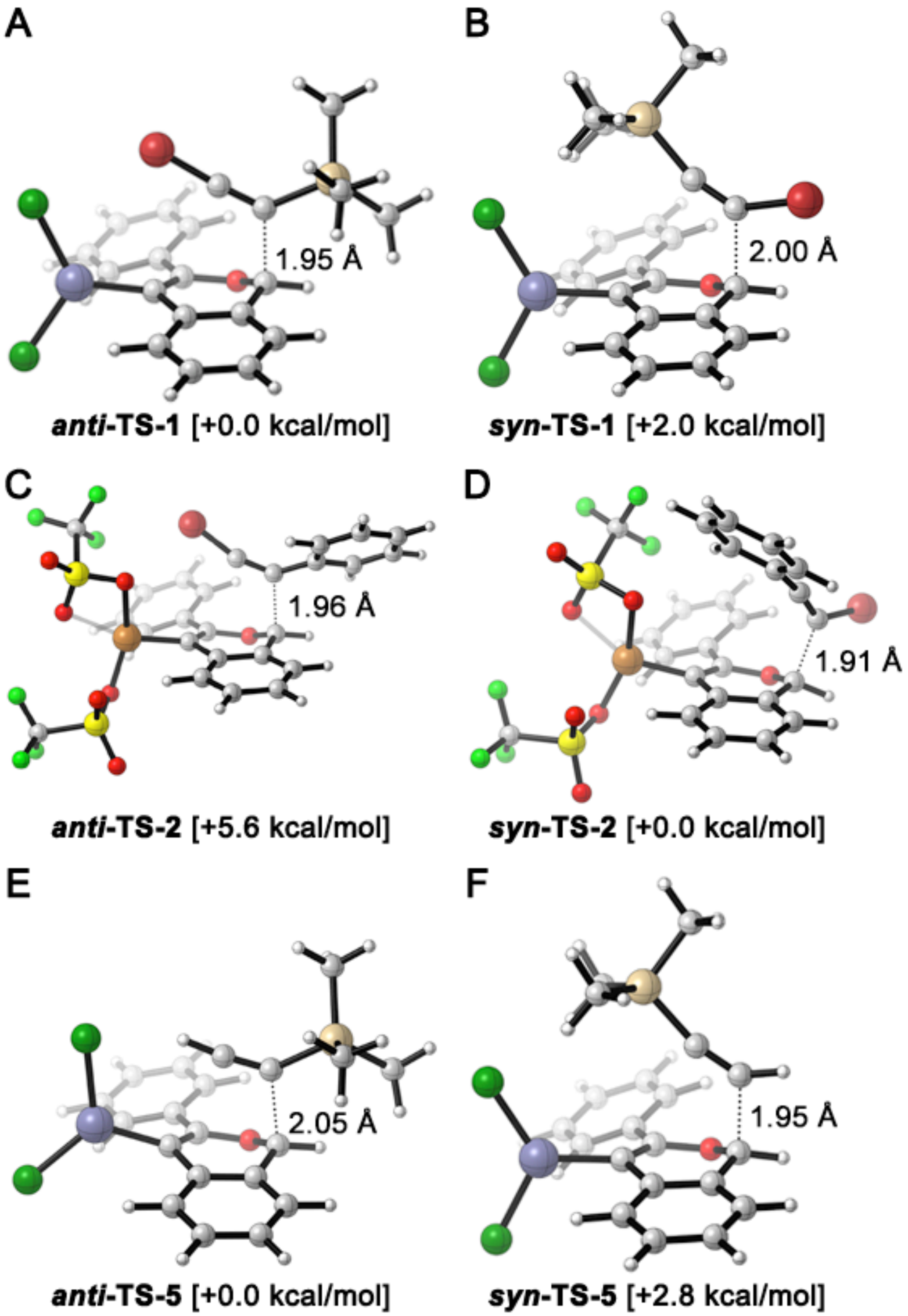


**Figure S73.** DFT calculated transition-states using B3LYP/6-31G(d) potentially responsible for the regioselectivity outcome in the benzannulation of: (A,B) silylhaloalkynes, (C,D) arylhaloalkynes, & (E,F) silylalkynes, along with their relative electronic energies and bond forming interatomic distances. Element coloring scheme: C = silver, H = white, O = light red, Zn = blue, Cl = dark green, F = light green, Si = beige, S = yellow, Br = dark red, Cu = bronze.

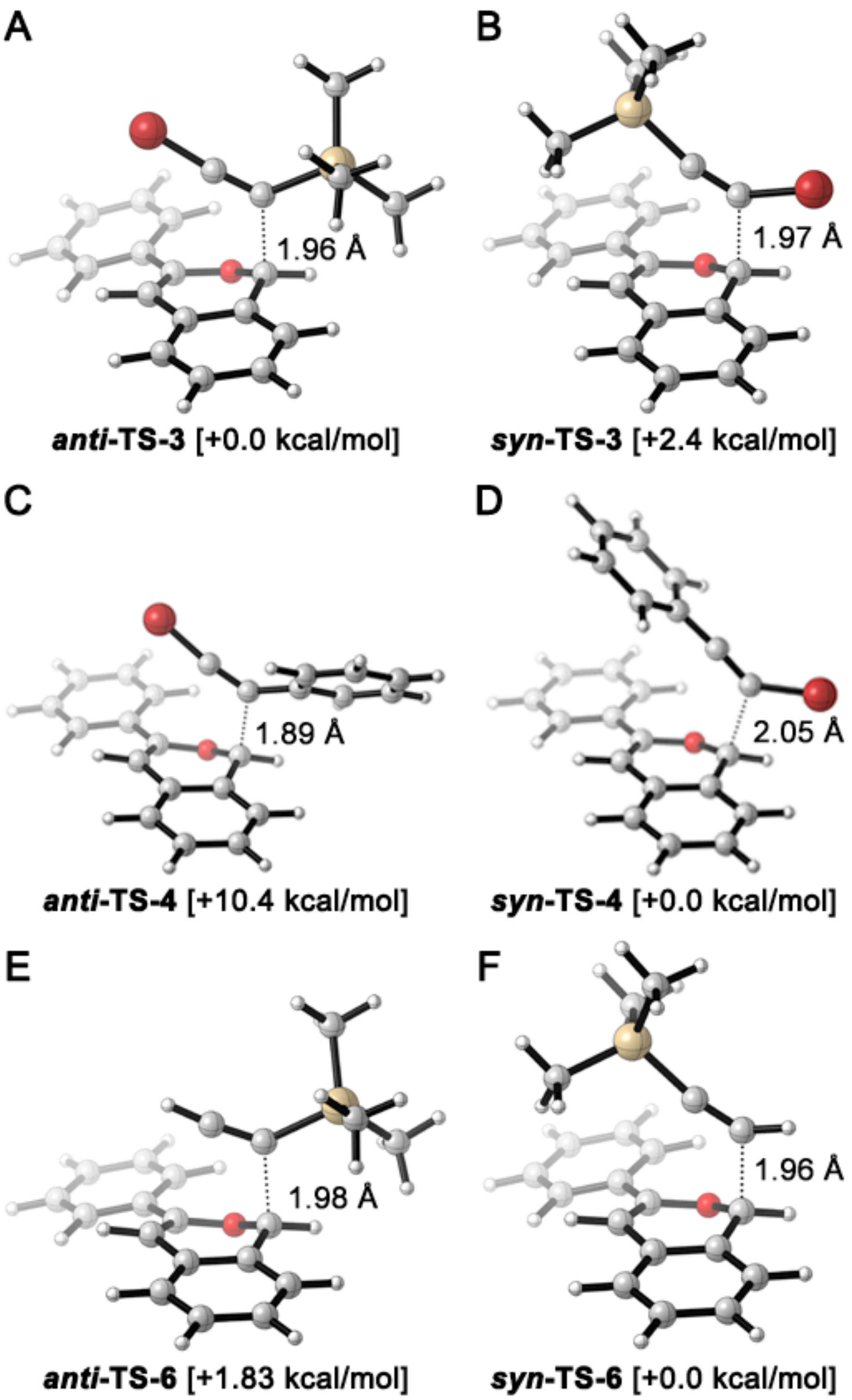


**Figure S74.** DFT calculated transition-states using B3LYP/6-31G(d) potentially responsible for the regioselectivity outcome in the benzannulation of: (A,B) silylhaloalkynes, (C,D) arylhaloalkynes, & (E,F) silylalkynes along with their relative electronic energies and bond forming interatomic distances. Element coloring scheme: C = silver, H = white, O = light red, Cl = dark green, F = light green, Si = beige, S = yellow, Br = dark red.

## Cartesian Coordinates of DFT Optimized Structures

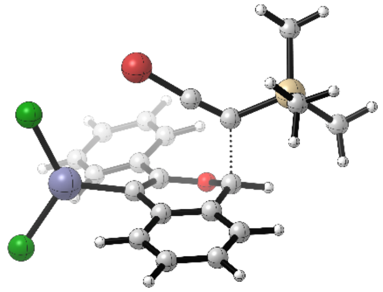

Structure: ***anti*-TS-1** (TMS-CC-Br Zn-major TS)
Charge: 0
Spin Multiplicity: 1
Computational Method/Basis Set: B3LYP/6-31G(d)
Solvation: gas phase
Number of Imaginary Frequencies: 1 (Freq. = –302.30)
Electronic Energy (AU): -6409.54186819
Gibbs Free Energy at 298.150 K (AU): -6409.275230

| | | | |
|---|---|---|---|
| C | 0.328283 | 3.192460 | -0.363978 |
| C | -0.059991 | 1.886692 | -0.743299 |
| C | -1.360461 | 1.713337 | -1.285198 |
| C | -2.242944 | 2.798186 | -1.432926 |
| C | -1.829113 | 4.066618 | -1.061650 |
| C | -0.538739 | 4.259183 | -0.527115 |
| H | 1.331176 | 3.349286 | 0.023775 |
| H | -3.231576 | 2.641238 | -1.858556 |
| H | -2.494409 | 4.915498 | -1.188905 |
| H | -0.220215 | 5.258028 | -0.243211 |
| C | 0.811018 | 0.746165 | -0.591548 |
| C | 0.462086 | -0.391190 | -1.281983 |
| O | -0.737389 | -0.487346 | -1.967615 |
| C | -1.729155 | 0.356454 | -1.616174 |
| H | -2.598158 | 0.191426 | -2.244796 |
| C | 1.278354 | -1.601023 | -1.460269 |
| C | 0.676454 | -2.846309 | -1.718636 |
| C | 2.680591 | -1.534244 | -1.371747 |
| C | 1.455131 | -3.991631 | -1.858107 |
| H | -0.403565 | -2.911871 | -1.794479 |
| C | 3.455220 | -2.682957 | -1.508883 |
| H | 3.175894 | -0.574816 | -1.252720 |
| C | 2.845893 | -3.915893 | -1.747846 |
| H | 0.975137 | -4.947894 | -2.047440 |
| H | 4.536245 | -2.608180 | -1.440237 |
| H | 3.451104 | -4.811729 | -1.854839 |
| C | -2.382302 | -0.423060 | 0.046077 |

| | | | |
|---|---|---|---|
| C | -1.501307 | -0.709206 | 0.892356 |
| Br | -0.366782 | -1.114657 | 2.190584 |
| Zn | 2.492573 | 0.888633 | 0.469968 |
| Cl | 2.767386 | -0.012348 | 2.491202 |
| Cl | 4.132428 | 2.138341 | -0.351323 |
| Si | -4.271559 | -0.593386 | 0.168220 |
| C | -4.629482 | -2.096026 | 1.246477 |
| H | -4.172409 | -1.994984 | 2.237114 |
| H | -5.710207 | -2.218950 | 1.388396 |
| H | -4.245796 | -3.016547 | 0.792624 |
| C | -4.923404 | 0.990371 | 0.957139 |
| H | -4.623244 | 1.881137 | 0.394750 |
| H | -6.019534 | 0.972905 | 1.001529 |
| H | -4.547667 | 1.102377 | 1.980161 |
| C | -4.999501 | -0.837975 | -1.557654 |
| H | -4.523356 | -1.667626 | -2.092985 |
| H | -6.066002 | -1.079186 | -1.467429 |
| H | -4.928930 | 0.058490 | -2.184547 |

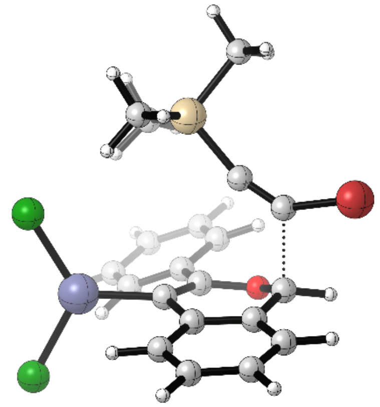

Structure: ***syn*-TS-1** (TMS-CC-Br Zn-minor TS)
Charge: 0
Spin Multiplicity: 1
Computational Method/Basis Set: B3LYP/6-31G(d)
Solvation: gas phase
Number of Imaginary Frequencies: 1 (Freq. = –414.29)
Electronic Energy (AU): -6409.53866341
Gibbs Free Energy at 298.150 K (AU): -6409.270880

| | | | |
|---|---|---|---|
| C | -0.098379 | -3.199821 | 0.282906 |
| C | 0.183740 | -1.990045 | -0.400039 |
| C | 1.431658 | -1.888600 | -1.080169 |
| C | 2.374327 | -2.936095 | -1.044972 |
| C | 2.061852 | -4.100438 | -0.370528 |
| C | 0.817960 | -4.232241 | 0.288985 |
| H | -1.060703 | -3.312831 | 0.773974 |

| | | | |
|---|---|---|---|
| H | 3.327570 | -2.822684 | -1.552683 |
| H | 2.770266 | -4.923276 | -0.351229 |
| H | 0.582902 | -5.157461 | 0.806706 |
| C | -0.736016 | -0.889955 | -0.412327 |
| C | -0.482753 | 0.119226 | -1.332255 |
| O | 0.652352 | 0.121346 | -2.101132 |
| C | 1.695008 | -0.636234 | -1.723249 |
| H | 2.511324 | -0.546317 | -2.433281 |
| C | -1.393966 | 1.201532 | -1.729529 |
| C | -0.891321 | 2.385279 | -2.302630 |
| C | -2.783770 | 1.064745 | -1.564809 |
| C | -1.755250 | 3.412269 | -2.669252 |
| H | 0.178420 | 2.499146 | -2.441226 |
| C | -3.643982 | 2.095955 | -1.933225 |
| H | -3.204517 | 0.131844 | -1.202244 |
| C | -3.133540 | 3.274074 | -2.480209 |
| H | -1.353134 | 4.325133 | -3.099678 |
| H | -4.714262 | 1.968869 | -1.802558 |
| H | -3.805704 | 4.078309 | -2.765735 |
| C | 1.778424 | 1.025325 | 0.535367 |
| C | 2.542139 | 0.420070 | -0.253116 |
| Br | 4.369635 | 0.111686 | -0.473662 |
| Zn | -2.396041 | -0.990078 | 0.696262 |
| Cl | -2.652679 | 0.104571 | 2.599518 |
| Cl | -3.968885 | -2.391450 | 0.004853 |
| Si | 1.137681 | 2.111892 | 1.928610 |
| C | -0.430773 | 3.024962 | 1.450832 |
| H | -0.639959 | 3.787160 | 2.212178 |
| H | -0.343925 | 3.531106 | 0.484036 |
| H | -1.288774 | 2.346673 | 1.423821 |
| C | 2.571844 | 3.331133 | 2.161951 |
| H | 2.349275 | 3.993696 | 3.008337 |
| H | 3.516091 | 2.819447 | 2.376887 |
| H | 2.717440 | 3.957356 | 1.275000 |
| C | 0.925511 | 1.022842 | 3.444821 |
| H | 0.741555 | 1.651259 | 4.325431 |
| H | 0.062957 | 0.358426 | 3.333382 |
| H | 1.821140 | 0.423273 | 3.642083 |

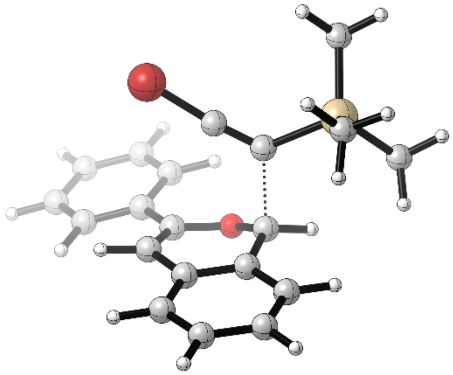

Structure: ***anti*-TS-3** (TMS-CC-Br metal-free major TS)
Charge: 1
Spin Multiplicity: 1
Computational Method/Basis Set: B3LYP/6-31G(d)
Solvation: gas phase
Number of Imaginary Frequencies: 1 (Freq. = –280.33)
Electronic Energy (AU): -3710.31570935
Gibbs Free Energy at 298.150 K (AU): -3710.031910

| | | | |
|---|---|---|---|
| C | -0.821001 | 3.522740 | 1.107783 |
| C | -0.380391 | 2.432932 | 0.328445 |
| C | -1.282769 | 1.838778 | -0.592670 |
| C | -2.589853 | 2.337343 | -0.740588 |
| C | -2.993883 | 3.422435 | 0.019531 |
| C | -2.108533 | 4.007574 | 0.949063 |
| H | -0.140889 | 3.984304 | 1.817662 |
| H | -3.266592 | 1.885644 | -1.460628 |
| H | -3.993465 | 3.827845 | -0.100392 |
| H | -2.441381 | 4.853480 | 1.542790 |
| C | 0.937374 | 1.882020 | 0.406821 |
| C | 1.399843 | 1.009610 | -0.544942 |
| O | 0.535193 | 0.574280 | -1.515378 |
| C | -0.799057 | 0.678620 | -1.304081 |
| H | -1.334420 | 0.340633 | -2.184084 |
| C | 2.760236 | 0.491678 | -0.692928 |
| C | 3.002575 | -0.658914 | -1.467082 |
| C | 3.844009 | 1.132784 | -0.062513 |
| C | 4.295106 | -1.158480 | -1.595785 |
| H | 2.176829 | -1.159177 | -1.960535 |
| C | 5.132286 | 0.624768 | -0.191690 |
| H | 3.687779 | 2.045192 | 0.504412 |
| C | 5.361903 | -0.521810 | -0.957280 |
| H | 4.470269 | -2.047226 | -2.194381 |
| H | 5.960797 | 1.132045 | 0.292982 |
| H | 6.369674 | -0.912662 | -1.061012 |
| C | -0.368604 | -1.071754 | 0.762514 |

| | | | |
|---|---|---|---|
| C | -1.195244 | -0.847936 | -0.148178 |
| H | 1.609406 | 2.212461 | 1.190075 |
| Br | 0.753711 | -1.531636 | 2.056338 |
| Si | -2.819045 | -1.798222 | -0.594665 |
| C | -4.168210 | -1.073521 | 0.497265 |
| H | -5.127339 | -1.553778 | 0.266547 |
| H | -3.956607 | -1.246616 | 1.557954 |
| H | -4.292028 | 0.004573 | 0.350453 |
| C | -2.483226 | -3.607208 | -0.215199 |
| H | -3.378311 | -4.205022 | -0.426398 |
| H | -1.667686 | -4.006610 | -0.827983 |
| H | -2.224557 | -3.761609 | 0.838073 |
| C | -3.178812 | -1.549365 | -2.427624 |
| H | -2.336964 | -1.845485 | -3.064331 |
| H | -4.026009 | -2.190000 | -2.703207 |
| H | -3.465794 | -0.523649 | -2.686654 |

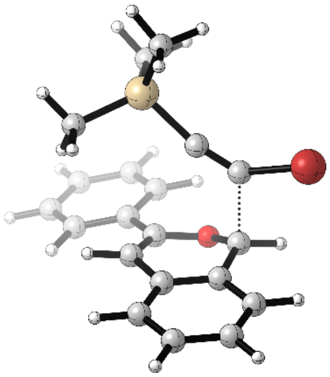

Structure: ***anti*-TS-3** (TMS-CC-Br metal-free minor TS)
Charge: 1
Spin Multiplicity: 1
Computational Method/Basis Set: B3LYP/6-31G(d)
Solvation: gas phase
Number of Imaginary Frequencies: 1 (Freq. = –391.07)
Electronic Energy (AU): -3710.31184235
Gibbs Free Energy at 298.150 K (AU): -3710.027611

| | | | |
|---|---|---|---|
| C | 1.528769 | -2.802645 | 1.873005 |
| C | 0.907542 | -2.088784 | 0.824802 |
| C | 1.687364 | -1.700938 | -0.302358 |
| C | 3.057828 | -2.017040 | -0.373148 |
| C | 3.636987 | -2.734203 | 0.658135 |
| C | 2.871087 | -3.122380 | 1.781246 |
| H | 0.944769 | -3.105227 | 2.737101 |
| H | 3.642885 | -1.705670 | -1.232463 |
| H | 4.687372 | -3.002614 | 0.608038 |
| H | 3.346971 | -3.680177 | 2.581974 |

| | | | |
|---|---|---|---|
| C | -0.467243 | -1.718241 | 0.826901 |
| C | -1.087421 | -1.264223 | -0.321063 |
| O | -0.323802 | -1.025098 | -1.420525 |
| C | 1.016219 | -0.889578 | -1.280384 |
| H | 1.474275 | -0.717927 | -2.249638 |
| C | -2.522443 | -1.094821 | -0.546899 |
| C | -2.984520 | -0.377977 | -1.668374 |
| C | -3.461193 | -1.656743 | 0.341167 |
| C | -4.350108 | -0.218633 | -1.883951 |
| H | -2.272448 | 0.053009 | -2.363042 |
| C | -4.824189 | -1.488299 | 0.121259 |
| H | -3.132462 | -2.251593 | 1.187244 |
| C | -5.272579 | -0.768607 | -0.989708 |
| H | -4.695562 | 0.335700 | -2.751127 |
| H | -5.537957 | -1.931525 | 0.808644 |
| H | -6.337367 | -0.643740 | -1.161893 |
| C | 0.422911 | 1.342677 | 0.234021 |
| C | 1.292950 | 0.928410 | -0.572081 |
| Br | 2.906013 | 1.492855 | -1.301208 |
| H | -1.063509 | -1.888351 | 1.714987 |
| Si | -0.737320 | 2.321623 | 1.346373 |
| C | -2.110010 | 3.024529 | 0.271652 |
| H | -2.768618 | 3.656597 | 0.879968 |
| H | -1.706076 | 3.646693 | -0.534277 |
| H | -2.726748 | 2.239468 | -0.178269 |
| C | 0.404226 | 3.670060 | 2.015499 |
| H | -0.183960 | 4.347936 | 2.647263 |
| H | 1.210504 | 3.254159 | 2.628254 |
| H | 0.852191 | 4.266091 | 1.214019 |
| C | -1.396746 | 1.230965 | 2.730524 |
| H | -0.591041 | 0.721421 | 3.269808 |
| H | -1.937345 | 1.851769 | 3.455271 |
| H | -2.100028 | 0.480956 | 2.353937 |

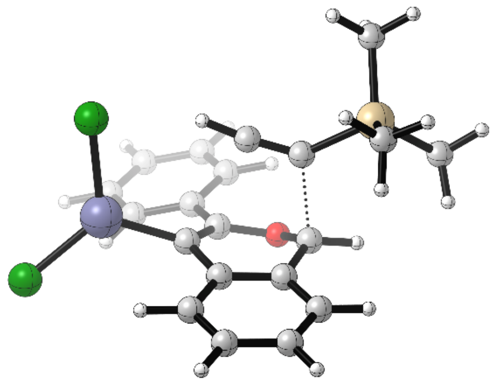

Structure: ***anti*-TS-5** (TMS-CC-H Zn-major TS)
Charge: 0

Spin Multiplicity: 1
Computational Method/Basis Set: B3LYP/6-31G(d)
Solvation: gas phase
Number of Imaginary Frequencies: 1 (Freq. = –340.79)
Electronic Energy (AU): -3838.44733237
Gibbs Free Energy at 298.150 K (AU): -3838.167853

| | | | |
|---|---|---|---|
| C | -0.057117 | -3.005101 | -0.734014 |
| C | 0.232759 | -1.629104 | -0.875668 |
| C | 1.499539 | -1.262995 | -1.400785 |
| C | 2.451996 | -2.236121 | -1.757013 |
| C | 2.131960 | -3.576271 | -1.622103 |
| C | 0.873608 | -3.957783 | -1.111681 |
| H | -1.028944 | -3.301321 | -0.347619 |
| H | 3.416631 | -1.933151 | -2.157175 |
| H | 2.849216 | -4.337932 | -1.913455 |
| H | 0.634704 | -5.012451 | -1.010692 |
| C | -0.688627 | -0.595538 | -0.474990 |
| C | -0.464350 | 0.665600 | -1.004559 |
| O | 0.694616 | 0.960592 | -1.675493 |
| C | 1.751808 | 0.149172 | -1.463311 |
| H | 2.632170 | 0.526026 | -1.972428 |
| C | -1.399659 | 1.798788 | -0.999060 |
| C | -0.929709 | 3.119466 | -1.119733 |
| C | -2.783207 | 1.578116 | -0.873716 |
| C | -1.820579 | 4.187983 | -1.094282 |
| H | 0.135196 | 3.300668 | -1.215045 |
| C | -3.669819 | 2.651242 | -0.844505 |
| H | -3.174197 | 0.565437 | -0.839188 |
| C | -3.191730 | 3.958365 | -0.952096 |
| H | -1.444348 | 5.203764 | -1.177287 |
| H | -4.734446 | 2.461449 | -0.747716 |
| H | -3.884304 | 4.795040 | -0.929236 |
| C | 2.163436 | 0.636722 | 0.482295 |
| C | 1.098611 | 0.609873 | 1.133109 |
| Zn | -2.201733 | -0.974548 | 0.766925 |
| Cl | -1.843700 | -0.203723 | 2.838784 |
| Cl | -3.973223 | -2.153342 | 0.214951 |
| Si | 3.999460 | 0.918815 | 0.826018 |
| C | 4.123204 | 2.165793 | 2.232773 |
| H | 3.612614 | 1.800649 | 3.130590 |
| H | 5.172303 | 2.351486 | 2.494003 |
| H | 3.671305 | 3.125455 | 1.958408 |
| C | 4.737622 | -0.747807 | 1.312579 |
| H | 4.586501 | -1.504934 | 0.535127 |
| H | 5.816671 | -0.655445 | 1.487818 |

| | | | |
|---|---|---|---|
| H | 4.277417 | -1.122413 | 2.233429 |
| C | 4.849257 | 1.600033 | -0.719670 |
| H | 4.341463 | 2.490021 | -1.109603 |
| H | 5.876177 | 1.894968 | -0.471030 |
| H | 4.917785 | 0.863662 | -1.529139 |
| H | 0.262348 | 0.584835 | 1.817247 |

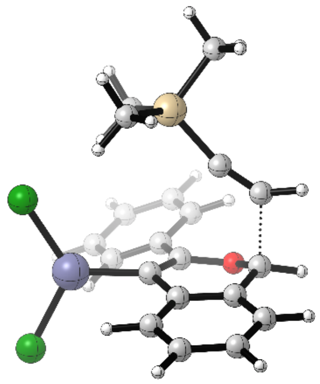

Structure: ***syn*-TS-5** (TMS-CC-H Zn-minor TS)
Charge: 0
Spin Multiplicity: 1
Computational Method/Basis Set: B3LYP/6-31G(d)
Solvation: gas phase
Number of Imaginary Frequencies: 1 (Freq. = –383.43)
Electronic Energy (AU): -3838.44280323
Gibbs Free Energy at 298.150 K (AU): -3838.162610

| | | | |
|---|---|---|---|
| C | 3.089708 | -0.892247 | 0.136652 |
| C | 2.081182 | -0.228125 | -0.603791 |
| C | 2.491981 | 0.763601 | -1.535763 |
| C | 3.848804 | 1.101483 | -1.698708 |
| C | 4.806502 | 0.430844 | -0.960735 |
| C | 4.421787 | -0.572535 | -0.044274 |
| H | 2.801924 | -1.682162 | 0.824616 |
| H | 4.135886 | 1.869312 | -2.413361 |
| H | 5.858193 | 0.668408 | -1.090596 |
| H | 5.183266 | -1.099457 | 0.523096 |
| C | 0.684767 | -0.531903 | -0.440194 |
| C | -0.171039 | -0.047802 | -1.414696 |
| O | 0.273540 | 0.776142 | -2.420402 |
| C | 1.435455 | 1.435483 | -2.243837 |
| H | 1.677353 | 2.008517 | -3.135117 |
| C | -1.578002 | -0.423351 | -1.624402 |
| C | -2.459426 | 0.448944 | -2.289732 |
| C | -2.060684 | -1.667548 | -1.182606 |
| C | -3.792309 | 0.095787 | -2.477945 |
| H | -2.095710 | 1.408270 | -2.642116 |

| | | | |
|---|---|---|---|
| C | -3.395672 | -2.016184 | -1.372826 |
| H | -1.383886 | -2.392826 | -0.740531 |
| C | -4.266588 | -1.134884 | -2.015140 |
| H | -4.464661 | 0.783514 | -2.983371 |
| H | -3.747575 | -2.983896 | -1.028510 |
| H | -5.307796 | -1.407772 | -2.162203 |
| C | 0.208420 | 2.698125 | -0.091988 |
| C | 0.955577 | 2.912754 | -1.072998 |
| Zn | 0.118570 | -1.823642 | 0.974918 |
| Cl | -0.851186 | -1.230379 | 2.872950 |
| Cl | 0.644804 | -3.952813 | 0.634676 |
| Si | -0.858086 | 2.869629 | 1.448907 |
| C | -2.445334 | 1.880196 | 1.294578 |
| H | -3.129447 | 2.179741 | 2.098604 |
| H | -2.953014 | 2.051095 | 0.339717 |
| H | -2.254661 | 0.809370 | 1.411255 |
| C | -1.234238 | 4.729778 | 1.453772 |
| H | -1.835782 | 4.972901 | 2.339064 |
| H | -0.321528 | 5.334037 | 1.495313 |
| H | -1.804709 | 5.031203 | 0.568517 |
| C | 0.177486 | 2.395233 | 2.941866 |
| H | -0.350349 | 2.687679 | 3.858346 |
| H | 0.331001 | 1.312782 | 2.988755 |
| H | 1.150087 | 2.899918 | 2.940200 |
| H | 1.497410 | 3.723055 | -1.533753 |

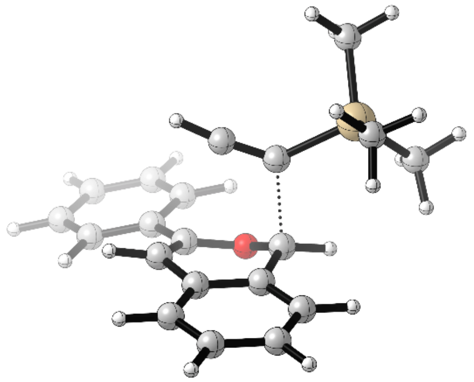

Structure: ***anti*-TS-6** (TMS-CC-H metal-free minor TS)
Charge: 1
Spin Multiplicity: 1
Computational Method/Basis Set: B3LYP/6-31G(d)
Solvation: gas phase
Number of Imaginary Frequencies: 1 (Freq. = –332.12)
Electronic Energy (AU): -1139.21502422
Gibbs Free Energy at 298.150 K (AU): -1138.917759

| | | | |
|---|---|---|---|
| C | 0.660472 | 3.480812 | -0.738593 |

| | | | |
|---|---|---|---|
| C | 0.240636 | 2.228035 | -0.245972 |
| C | 1.094981 | 1.510463 | 0.631287 |
| C | 2.340031 | 2.039693 | 1.014949 |
| C | 2.721555 | 3.283326 | 0.538780 |
| C | 1.882381 | 3.998258 | -0.341688 |
| H | 0.017126 | 4.037852 | -1.413372 |
| H | 2.982623 | 1.486023 | 1.693907 |
| H | 3.670306 | 3.713123 | 0.843873 |
| H | 2.200284 | 4.968601 | -0.710577 |
| C | -1.000926 | 1.606233 | -0.590454 |
| C | -1.507160 | 0.567296 | 0.167624 |
| O | -0.705757 | 0.005822 | 1.110674 |
| C | 0.636423 | 0.199618 | 1.014590 |
| H | 1.141026 | -0.332577 | 1.812919 |
| C | -2.857002 | 0.009222 | 0.118257 |
| C | -3.116605 | -1.265861 | 0.656705 |
| C | -3.912325 | 0.736654 | -0.465768 |
| C | -4.399193 | -1.801421 | 0.598692 |
| H | -2.311428 | -1.833079 | 1.109981 |
| C | -5.190577 | 0.192265 | -0.524017 |
| H | -3.743871 | 1.738881 | -0.847314 |
| C | -5.437031 | -1.077356 | 0.006288 |
| H | -4.589731 | -2.786581 | 1.013151 |
| H | -5.998691 | 0.763618 | -0.969990 |
| H | -6.437176 | -1.498055 | -0.036516 |
| C | 0.297652 | -0.763073 | -1.472375 |
| C | 1.079576 | -0.959939 | -0.523180 |
| H | -1.621309 | 2.037356 | -1.368016 |
| Si | 2.606482 | -2.098401 | -0.219838 |
| C | 4.107851 | -1.112072 | -0.779090 |
| H | 5.018825 | -1.699687 | -0.610743 |
| H | 4.055412 | -0.877908 | -1.847720 |
| H | 4.216192 | -0.169068 | -0.232624 |
| C | 2.322002 | -3.636221 | -1.260487 |
| H | 3.157506 | -4.335154 | -1.132613 |
| H | 1.405131 | -4.159054 | -0.967481 |
| H | 2.250834 | -3.395435 | -2.326737 |
| C | 2.687223 | -2.524971 | 1.614281 |
| H | 1.744038 | -2.937988 | 1.990384 |
| H | 3.453814 | -3.296027 | 1.760859 |
| H | 2.972341 | -1.677813 | 2.249010 |
| H | -0.327735 | -0.681772 | -2.337042 |

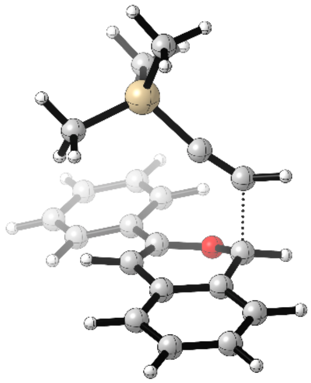

Structure: ***syn*-TS-6** (TMS-CC-H metal-free major TS)
Charge: 1
Spin Multiplicity: 1
Computational Method/Basis Set: B3LYP/6-31G(d)
Solvation: gas phase
Number of Imaginary Frequencies: 1 (Freq. = –360.07)
Electronic Energy (AU): -1139.21822776
Gibbs Free Energy at 298.150 K (AU): -1138.920177

| | | | |
|---|---|---|---|
| C | 3.032181 | -1.070805 | 1.657496 |
| C | 2.059428 | -0.990244 | 0.637494 |
| C | 2.488235 | -0.871518 | -0.713169 |
| C | 3.858164 | -0.836400 | -1.033849 |
| C | 4.792154 | -0.938244 | -0.018164 |
| C | 4.375619 | -1.050190 | 1.327332 |
| H | 2.718386 | -1.161740 | 2.693192 |
| H | 4.174111 | -0.746401 | -2.069593 |
| H | 5.851711 | -0.932192 | -0.253028 |
| H | 5.123250 | -1.122243 | 2.111293 |
| C | 0.653573 | -1.015185 | 0.877527 |
| C | -0.243207 | -1.208597 | -0.151835 |
| O | 0.225398 | -1.224029 | -1.430226 |
| C | 1.446619 | -0.698904 | -1.690874 |
| H | 1.676954 | -0.816783 | -2.745645 |
| C | -1.673270 | -1.499752 | -0.042149 |
| C | -2.515266 | -1.354110 | -1.161712 |
| C | -2.224241 | -1.938688 | 1.177636 |
| C | -3.875613 | -1.628746 | -1.055283 |
| H | -2.101545 | -1.021423 | -2.107075 |
| C | -3.585587 | -2.206880 | 1.276762 |
| H | -1.587609 | -2.101979 | 2.041555 |
| C | -4.414793 | -2.051732 | 0.162467 |
| H | -4.516413 | -1.512233 | -1.923905 |
| H | -3.997843 | -2.551999 | 2.219856 |
| H | -5.476039 | -2.267534 | 0.241299 |
| C | 0.396934 | 1.723433 | -0.833307 |

| | | | |
|---|---|---|---|
| C | 1.167014 | 1.238292 | -1.691078 |
| H | 0.285269 | -0.984205 | 1.895647 |
| Si | -0.757898 | 2.736450 | 0.262194 |
| C | -2.492841 | 2.591604 | -0.446279 |
| H | -3.180860 | 3.219116 | 0.133409 |
| H | -2.530942 | 2.932605 | -1.486482 |
| H | -2.868551 | 1.563845 | -0.408692 |
| C | -0.058409 | 4.481359 | 0.090431 |
| H | -0.676921 | 5.171981 | 0.677412 |
| H | 0.967768 | 4.549268 | 0.466159 |
| H | -0.068910 | 4.826453 | -0.948488 |
| C | -0.661725 | 2.131053 | 2.041845 |
| H | 0.371892 | 2.050377 | 2.394545 |
| H | -1.177981 | 2.845846 | 2.694093 |
| H | -1.154026 | 1.160834 | 2.165436 |
| H | 1.825864 | 1.545858 | -2.487649 |

## F. MALDI-TOF Mass Spectra

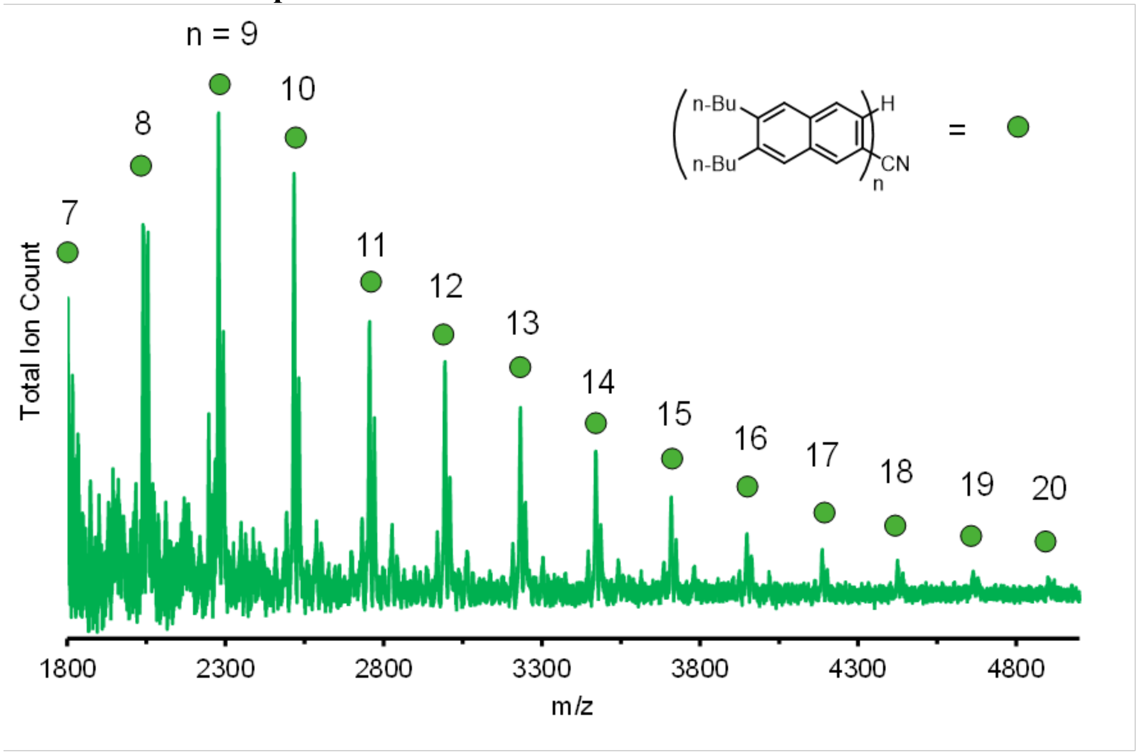


**Figure S75**: MALDI-TOF-MS of **8** using a matrix of 2,5-dihydroxybenzoic acid and Ag(OCOCF$_3$) additive.

## G. Single Crystal X-Ray Structure

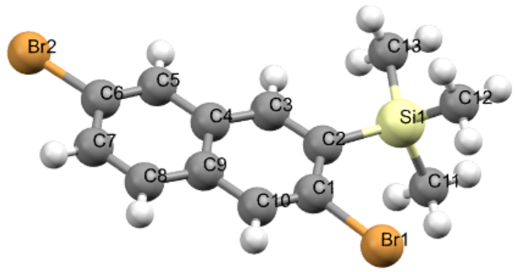


**Figure S76**: Single crystal X-ray structure of **3c** taken at 100 K.

**Table S2:** Crystal data and structure refinement for CCDC 1539451

| | |
|---|---|
| Empirical formula | $C_{13}H_{14}SiBr_2$ |
| Formula weight | 358.15 |
| Temperature / K | 100.0 |
| Crystal system | monoclinic |
| Space group | $P2_1/n$ |
| a / Å, b / Å, c / Å | 7.1961(4), 7.4763(4), 26.1123(14) |
| α/°, β/°, γ/° | 90, 95.1754(14), 90 |
| Volume / Å$^3$ | 1399.12(13) |
| Z | 4 |
| $\rho_{calc}$ / mg mm$^{-3}$ | 1.700 |
| μ / mm$^{-1}$ | 5.852 |
| F(000) | 704 |
| Crystal size / mm$^3$ | 0.193 × 0.06 × 0.033 |
| 2Θ range for data collection | 3.132 to 60.396° |
| Index ranges | $-10 \le h \le 9$, $-10 \le k \le 10$, $-36 \le l \le 36$ |
| Reflections collected | 48708 |
| Independent reflections | 4154[R(int) = 0.0365] |
| Data/restraints/parameters | 4154/0/148 |
| Goodness-of-fit on F$^2$ | 1.036 |
| Final R indexes [I>2σ (I)] | $R_1$ = 0.0193, $wR_2$ = 0.0416 |
| Final R indexes [all data] | $R_1$ = 0.0256, $wR_2$ = 0.0431 |
| Largest diff. peak/hole / e Å$^{-3}$ | 0.435/-0.450 |

**Table S3** Fractional Atomic Coordinates (×10$^4$) and Equivalent Isotropic Displacement Parameters (Å$^2$×10$^3$) for CCDC 1539451. $U_{eq}$ is defined as 1/3 of of the trace of the orthogonalised $U_{IJ}$ tensor.

| Atom | *x* | *y* | *z* | U(eq) |
|---|---|---|---|---|
| Br1 | -290.5(2) | 5554.7(2) | 6333.1(2) | 22.81(4) |
| Br2 | 9648.2(2) | 7293.9(2) | 8442.8(2) | 20.57(4) |
| Si1 | 3325.1(5) | 7401.5(6) | 5713.7(2) | 18.60(8) |
| C1 | 2086.7(18) | 6049.6(18) | 6691.0(6) | 17.3(3) |
| C2 | 3511.0(18) | 6853.5(18) | 6423.5(5) | 15.6(3) |
| C3 | 5161.6(18) | 7211.1(17) | 6718.0(5) | 14.8(2) |
| C4 | 5460.2(18) | 6784.8(17) | 7247.7(5) | 14.5(2) |
| C5 | 7176.8(18) | 7171.1(17) | 7538.1(6) | 16.0(3) |
| C6 | 7381.7(19) | 6731.2(18) | 8047.6(6) | 16.6(3) |
| C7 | 5952(2) | 5897.2(18) | 8296.1(6) | 18.7(3) |
| C8 | 4292(2) | 5519.8(18) | 8021.9(6) | 18.4(3) |
| C9 | 3995.6(18) | 5957.4(17) | 7492.3(5) | 15.6(3) |
| C10 | 2284.4(19) | 5608.7(18) | 7199.4(6) | 18.0(3) |
| C11 | 1567(2) | 9198(3) | 5562.6(7) | 30.4(4) |
| C12 | 2749(2) | 5350(3) | 5323.9(7) | 35.8(4) |
| C13 | 5641(2) | 8246(2) | 5549.0(6) | 20.7(3) |

**Table S4** Anisotropic Displacement Parameters (Å$^2$×10$^3$) for CCDC 1539451. The Anisotropic displacement factor exponent takes the form: $-2\pi^2[h^2a^{*2}U_{11}+2hka^*b^*U_{12}+\ldots]$.

| Atom | $U_{11}$ | $U_{22}$ | $U_{33}$ | $U_{23}$ | $U_{13}$ | $U_{12}$ |
|---|---|---|---|---|---|---|
| Br1 | 12.27(6) | 23.85(8) | 32.01(9) | 3.09(6) | 0.34(5) | -1.88(5) |
| Br2 | 20.93(7) | 17.95(7) | 22.24(8) | 2.78(5) | -1.33(5) | -3.52(5) |
| Si1 | 13.76(17) | 23.9(2) | 18.22(19) | -2.09(15) | 1.88(14) | 1.78(14) |
| C1 | 11.9(6) | 12.6(6) | 27.3(7) | -0.7(5) | 1.5(5) | 1.5(5) |
| C2 | 14.3(6) | 11.8(6) | 21.2(7) | 0.1(5) | 3.7(5) | 2.9(5) |
| C3 | 13.8(6) | 11.9(6) | 19.5(6) | 0.3(5) | 5.7(5) | 1.3(5) |
| C4 | 14.4(6) | 9.5(5) | 20.3(7) | 0.2(5) | 4.6(5) | 1.1(4) |
| C5 | 14.8(6) | 12.1(6) | 21.5(7) | 0.6(5) | 4.6(5) | -0.7(5) |
| C6 | 16.4(6) | 11.5(6) | 22.1(7) | -0.9(5) | 2.3(5) | 0.3(5) |
| C7 | 22.1(7) | 15.7(6) | 19.1(7) | 2.6(5) | 6.3(5) | 0.9(5) |
| C8 | 17.4(6) | 14.8(6) | 24.3(7) | 3.3(5) | 8.6(5) | -0.2(5) |
| C9 | 15.4(6) | 11.3(6) | 20.9(7) | 1.1(5) | 5.5(5) | 1.6(5) |
| C10 | 13.7(6) | 14.1(6) | 26.9(7) | 2.4(5) | 6.5(5) | 0.2(5) |
| C11 | 21.7(7) | 45(1) | 24.6(8) | 12.1(7) | 3.3(6) | 11.0(7) |
| C12 | 26.3(8) | 46.7(11) | 35.3(10) | -18.9(8) | 7.7(7) | -9.5(8) |
| C13 | 18.0(6) | 26.5(7) | 18.0(7) | 0.0(6) | 3.1(5) | 1.3(6) |

**Table S5** Bond Lengths for CCDC 1539451.

| Atom | Atom | Length/Å | Atom | Atom | Length/Å |
|---|---|---|---|---|---|
| Br1 | C1 | 1.9101(14) | C3 | C4 | 1.417(2) |
| Br2 | C6 | 1.8969(14) | C4 | C5 | 1.4196(19) |
| Si1 | C2 | 1.8911(15) | C4 | C9 | 1.4224(19) |
| Si1 | C11 | 1.8632(17) | C5 | C6 | 1.366(2) |
| Si1 | C12 | 1.8664(18) | C6 | C7 | 1.4105(19) |
| Si1 | C13 | 1.8683(15) | C7 | C8 | 1.365(2) |
| C1 | C2 | 1.4244(19) | C8 | C9 | 1.418(2) |
| C1 | C10 | 1.363(2) | C9 | C10 | 1.414(2) |
| C2 | C3 | 1.3816(19) | | | |

**Table S6** Bond Angles for CCDC 1539451.

| Atom | Atom | Atom | Angle/° | Atom | Atom | Atom | Angle/° |
|---|---|---|---|---|---|---|---|
| C11 | Si1 | C2 | 110.51(7) | C3 | C4 | C5 | 121.58(12) |
| C11 | Si1 | C12 | 111.33(9) | C3 | C4 | C9 | 118.88(13) |
| C11 | Si1 | C13 | 108.08(8) | C5 | C4 | C9 | 119.53(13) |
| C12 | Si1 | C2 | 110.44(8) | C6 | C5 | C4 | 118.93(12) |
| C12 | Si1 | C13 | 108.12(7) | C5 | C6 | Br2 | 119.25(10) |
| C13 | Si1 | C2 | 108.25(6) | C5 | C6 | C7 | 122.39(13) |
| C2 | C1 | Br1 | 119.61(11) | C7 | C6 | Br2 | 118.35(11) |
| C10 | C1 | Br1 | 116.11(10) | C8 | C7 | C6 | 119.33(14) |
| C10 | C1 | C2 | 124.28(13) | C7 | C8 | C9 | 120.77(13) |
| C1 | C2 | Si1 | 125.57(11) | C8 | C9 | C4 | 119.05(13) |
| C3 | C2 | Si1 | 119.00(10) | C10 | C9 | C4 | 118.80(13) |
| C3 | C2 | C1 | 115.42(13) | C10 | C9 | C8 | 122.15(13) |
| C2 | C3 | C4 | 123.16(12) | C1 | C10 | C9 | 119.45(13) |

**Table S7** Hydrogen Atom Coordinates (Å×$10^4$) and Isotropic Displacement Parameters (Å$^2$×$10^3$) for CCDC 1539451.

| **Atom** | ***x*** | ***y*** | ***z*** | **U(eq)** |
|---|---|---|---|---|
| H3 | 6143 | 7770 | 6558 | 18 |
| H5 | 8167 | 7727 | 7380 | 19 |
| H7 | 6141 | 5601 | 8651 | 22 |
| H8 | 3326 | 4958 | 8188 | 22 |
| H10 | 1281 | 5072 | 7356 | 22 |
| H11A | 329 | 8763 | 5631 | 46 |
| H11B | 1557 | 9531 | 5199 | 46 |
| H11C | 1890 | 10246 | 5778 | 46 |
| H12A | 3669 | 4416 | 5421 | 54 |
| H12B | 2773 | 5631 | 4958 | 54 |
| H12C | 1502 | 4928 | 5388 | 54 |
| H13A | 5936 | 9366 | 5734 | 31 |
| H13B | 5598 | 8460 | 5178 | 31 |
| H13C | 6605 | 7355 | 5649 | 31 |

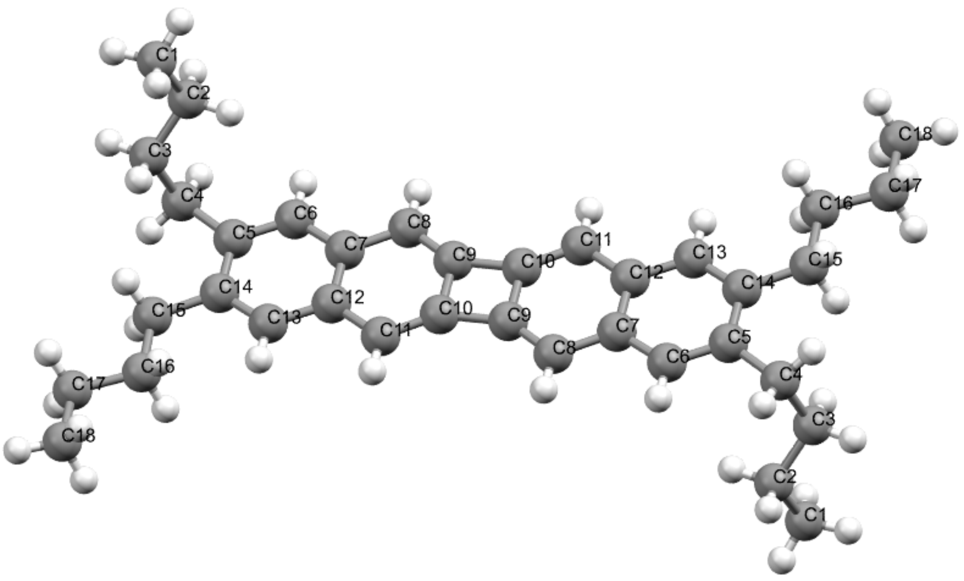


**Figure S77**: Single crystal X-ray structure of **8** taken at 100 K.

**Table S8:** Crystal data and structure refinement for CCDC 1539450

| | |
|---|---|
| Empirical formula | $C_{36}H_{44}$ |
| Formula weight | 476.71 |
| Temperature / K | 100.0 |
| Crystal system | triclinic |
| Space group | P-1 |
| a / Å, b / Å, c / Å | 8.2828(4), 8.9132(4), 9.1524(4) |
| α/°, β/°, γ/° | 88.129(2), 79.474(2), 86.662(2) |
| Volume / Å$^3$ | 663.02(5) |
| Z | 1 |
| $\rho_{calc}$ / mg mm$^{-3}$ | 1.194 |
| μ / mm$^{-1}$ | 0.067 |
| F(000) | 260 |
| Crystal size / mm$^3$ | 0.486 × 0.281 × 0.09 |
| 2Θ range for data collection | 4.578 to 51.55° |
| Index ranges | $-10 \le h \le 10$, $-10 \le k \le 10$, $-11 \le l \le 11$ |
| Reflections collected | 14580 |
| Independent reflections | 2529[R(int) = 0.0545] |
| Data/restraints/parameters | 2529/0/165 |
| Goodness-of-fit on F$^2$ | 1.075 |
| Final R indexes [I>2σ (I)] | $R_1$ = 0.0481, w$R_2$ = 0.1353 |
| Final R indexes [all data] | $R_1$ = 0.0495, w$R_2$ = 0.1367 |
| Largest diff. peak/hole / e Å$^{-3}$ | 0.350/-0.223 |

**Table S9** Fractional Atomic Coordinates ($\times10^4$) and Equivalent Isotropic Displacement Parameters ($Å^2\times10^3$) for CCDC 1539450. $U_{eq}$ is defined as 1/3 of of the trace of the orthogonalised $U_{IJ}$ tensor.

| **Atom** | ***x*** | ***y*** | ***z*** | **U(eq)** |
|---|---|---|---|---|
| C1 | 7242.7(18) | 5871.2(15) | 10336.7(16) | 21.2(3) |
| C2 | 5985.0(17) | 6981.8(15) | 9815.8(15) | 19.6(3) |
| C3 | 6797.0(16) | 8102.7(15) | 8660.3(15) | 17.0(3) |
| C4 | 5584.0(16) | 9125.1(14) | 7947.3(14) | 15.2(3) |
| C5 | 4811.2(16) | 8307.1(14) | 6852.6(15) | 13.7(3) |
| C6 | 3272.2(16) | 7752.9(14) | 7295.0(14) | 13.8(3) |
| C7 | 2508.4(16) | 6919.6(14) | 6358.8(14) | 13.7(3) |
| C8 | 897.1(15) | 6369.8(14) | 6870.9(14) | 13.9(3) |
| C9 | 263.7(15) | 5580.9(14) | 5889.4(15) | 13.8(3) |
| C10 | 1150.3(16) | 5270.4(14) | 4411.9(15) | 13.9(3) |
| C11 | 2673.6(16) | 5754.6(14) | 3894.2(14) | 14.4(3) |
| C12 | 3378.5(15) | 6625.1(14) | 4893.2(14) | 13.7(3) |
| C13 | 4946.7(16) | 7220.3(14) | 4450.1(14) | 14.1(3) |
| C14 | 5670.3(15) | 8048.0(13) | 5378.6(14) | 13.5(3) |
| C15 | 7339.1(15) | 8697.4(14) | 4859.1(14) | 14.8(3) |
| C16 | 8206.6(16) | 8294.8(14) | 3299.7(15) | 15.1(3) |
| C17 | 9920.3(16) | 8907.7(14) | 2906.3(15) | 17.1(3) |
| C18 | 10833.4(17) | 8353.3(16) | 1404.9(16) | 21.0(3) |

**Table S10** Anisotropic Displacement Parameters ($Å^2×10^3$) for CCDC 1539450. The Anisotropic displacement factor exponent takes the form: $-2\pi^2[h^2a^{*2}U_{11}+2hka^*b^*U_{12}+\ldots]$.

| Atom | $U_{11}$ | $U_{22}$ | $U_{33}$ | $U_{23}$ | $U_{13}$ | $U_{12}$ |
|---|---|---|---|---|---|---|
| C1 | 27.0(7) | 15.0(7) | 23.2(7) | 0.3(5) | -7.8(6) | -4.1(5) |
| C2 | 20.8(7) | 16.2(7) | 21.5(7) | 1.7(5) | -2.5(5) | -4.0(5) |
| C3 | 17.6(6) | 15.6(6) | 18.1(6) | -0.7(5) | -3.1(5) | -4.8(5) |
| C4 | 17.4(6) | 11.8(6) | 16.1(6) | -2.1(5) | -0.9(5) | -3.2(5) |
| C5 | 15.9(6) | 7.5(6) | 18.0(6) | 0.2(5) | -4.0(5) | 0.4(5) |
| C6 | 15.6(6) | 10.5(6) | 14.6(6) | -0.8(5) | -0.8(5) | 0.3(5) |
| C7 | 14.6(6) | 8.6(6) | 17.3(6) | 1.0(5) | -2.1(5) | 0.5(5) |
| C8 | 14.8(6) | 10.9(6) | 15.1(6) | -0.2(5) | -0.3(5) | -0.6(5) |
| C9 | 13.0(6) | 9.1(6) | 18.6(6) | 1.4(5) | -1.2(5) | -0.9(5) |
| C10 | 16.2(6) | 9.0(6) | 16.9(6) | -1.4(5) | -3.8(5) | -0.5(5) |
| C11 | 16.0(6) | 11.0(6) | 15.4(6) | -1.1(5) | -0.7(5) | -0.9(5) |
| C12 | 15.2(6) | 8.1(6) | 17.5(7) | 0.5(5) | -2.3(5) | -0.3(5) |
| C13 | 14.9(6) | 10.6(6) | 15.8(6) | -0.5(5) | -0.4(5) | -0.6(5) |
| C14 | 13.4(6) | 8.4(6) | 18.1(7) | 0.8(5) | -1.7(5) | 0.0(5) |
| C15 | 15.1(6) | 11.0(6) | 18.2(7) | -0.8(5) | -2.2(5) | -2.9(5) |
| C16 | 15.1(6) | 10.8(6) | 19.0(7) | -1.2(5) | -1.5(5) | -3.2(5) |
| C17 | 16.5(7) | 11.8(6) | 22.2(7) | -2.4(5) | 0.0(5) | -3.4(5) |
| C18 | 18.9(7) | 17.9(7) | 24.2(7) | -3.6(5) | 3.2(5) | -4.5(5) |

**Table S11** Bond Lengths for CCDC 1539450.

| Atom | Atom | Length/Å | Atom | Atom | Length/Å |
|---|---|---|---|---|---|
| C1 | C2 | 1.5242(19) | C9 | $C10^1$ | 1.5028(18) |
| C2 | C3 | 1.5226(18) | C10 | $C9^1$ | 1.5029(18) |
| C3 | C4 | 1.5362(18) | C10 | C11 | 1.3544(18) |
| C4 | C5 | 1.5104(18) | C11 | C12 | 1.4398(18) |
| C5 | C6 | 1.3801(18) | C12 | C13 | 1.4166(18) |
| C5 | C14 | 1.4248(18) | C13 | C14 | 1.3799(18) |
| C6 | C7 | 1.4073(18) | C14 | C15 | 1.5189(17) |
| C7 | C8 | 1.4400(17) | C15 | C16 | 1.5201(18) |
| C7 | C12 | 1.4266(18) | C16 | C17 | 1.5276(17) |
| C8 | C9 | 1.3539(19) | C17 | C18 | 1.5250(18) |
| C9 | C10 | 1.4431(18) | | | |

$^1$1-X,1-Y,1-Z

**Table S12** Bond Angles for CCDC 1539450.

| Atom | Atom | Atom | Angle/° | Atom | Atom | Atom | Angle/° |
|---|---|---|---|---|---|---|---|
| C3 | C2 | C1 | 111.85(11) | C9 | C10 | C9[1] | 89.93(11) |
| C2 | C3 | C4 | 114.32(11) | C11 | C10 | C9[1] | 147.36(13) |
| C5 | C4 | C3 | 112.48(10) | C11 | C10 | C9 | 122.70(12) |
| C6 | C5 | C4 | 119.78(12) | C10 | C11 | C12 | 116.53(12) |
| C6 | C5 | C14 | 119.12(12) | C7 | C12 | C11 | 120.75(12) |
| C14 | C5 | C4 | 121.05(11) | C13 | C12 | C7 | 118.10(12) |
| C5 | C6 | C7 | 122.95(12) | C13 | C12 | C11 | 121.15(12) |
| C6 | C7 | C8 | 120.86(12) | C14 | C13 | C12 | 122.90(12) |
| C6 | C7 | C12 | 118.21(12) | C5 | C14 | C15 | 119.71(11) |
| C12 | C7 | C8 | 120.93(12) | C13 | C14 | C5 | 118.69(12) |
| C9 | C8 | C7 | 116.54(12) | C13 | C14 | C15 | 121.60(11) |
| C8 | C9 | C10[1] | 147.39(12) | C14 | C15 | C16 | 116.08(11) |
| C8 | C9 | C10 | 122.53(12) | C15 | C16 | C17 | 112.60(11) |
| C10 | C9 | C10[1] | 90.07(11) | C18 | C17 | C16 | 111.58(11) |

$^{1}$1-X,1-Y,1-Z

**Table S13** Torsion Angles for CCDC 1539450.

| A | B | C | D | Angle/° | A | B | C | D | Angle/° |
|---|---|---|---|---|---|---|---|---|---|
| C1 | C2 | C3 | C4 | 172.70(11) | C8 | C7 | C12 | C11 | -0.72(18) |
| C2 | C3 | C4 | C5 | -73.99(14) | C8 | C7 | C12 | C13 | 178.92(11) |
| C3 | C4 | C5 | C6 | 97.61(14) | C8 | C9 | C10 | C9[1] | 179.23(15) |
| C3 | C4 | C5 | C14 | -80.10(14) | C8 | C9 | C10 | C11 | -0.5(2) |
| C4 | C5 | C6 | C7 | -176.95(11) | C9[1] | C10 | C11 | C12 | 179.69(17) |
| C4 | C5 | C14 | C13 | 176.33(11) | C9 | C10 | C11 | C12 | -0.74(19) |
| C4 | C5 | C14 | C15 | -4.39(18) | C10[1] | C9 | C10 | C9[1] | 0.000(1) |
| C5 | C6 | C7 | C8 | -179.77(11) | C10[1] | C9 | C10 | C11 | -179.77(15) |
| C5 | C6 | C7 | C12 | 0.68(19) | C10 | C11 | C12 | C7 | 1.33(18) |
| C5 | C14 | C15 | C16 | 176.84(11) | C10 | C11 | C12 | C13 | -178.31(11) |
| C6 | C5 | C14 | C13 | -1.40(18) | C11 | C12 | C13 | C14 | -179.39(11) |
| C6 | C5 | C14 | C15 | 177.88(11) | C12 | C7 | C8 | C9 | -0.53(18) |
| C6 | C7 | C8 | C9 | 179.94(11) | C12 | C13 | C14 | C5 | 0.51(19) |
| C6 | C7 | C12 | C11 | 178.82(11) | C12 | C13 | C14 | C15 | -178.75(11) |
| C6 | C7 | C12 | C13 | -1.53(18) | C13 | C14 | C15 | C16 | -3.91(18) |
| C7 | C8 | C9 | C10 | 1.16(18) | C14 | C5 | C6 | C7 | 0.81(19) |
| C7 | C8 | C9 | C10[1] | 179.73(17) | C14 | C15 | C16 | C17 | -176.24(10) |
| C7 | C12 | C13 | C14 | 0.96(19) | C15 | C16 | C17 | C18 | 174.05(11) |

$^{1}$1-X,1-Y,1-Z

**Table 14** Hydrogen Atom Coordinates (Å×$10^4$) and Isotropic Displacement Parameters (Å$^2$×$10^3$) for CCDC 1539450.

| Atom | *x* | *y* | *z* | U(eq) |
|---|---|---|---|---|
| H1A | 7742 | 5218 | 9516 | 32 |
| H1B | 6696 | 5258 | 11169 | 32 |
| H1C | 8100 | 6424 | 10660 | 32 |
| H2A | 5193 | 6422 | 9384 | 24 |
| H2B | 5364 | 7534 | 10681 | 24 |
| H3A | 7537 | 7541 | 7866 | 20 |
| H3B | 7483 | 8740 | 9136 | 20 |
| H4A | 4706 | 9534 | 8739 | 18 |
| H4B | 6170 | 9983 | 7429 | 18 |
| H6 | 2703 | 7943 | 8276 | 17 |
| H8 | 309 | 6551 | 7847 | 17 |
| H11 | 3247 | 5531 | 2922 | 17 |
| H13 | 5526 | 7042 | 3470 | 17 |
| H15A | 8065 | 8356 | 5565 | 18 |
| H15B | 7198 | 9806 | 4910 | 18 |
| H16A | 7532 | 8702 | 2575 | 18 |
| H16B | 8301 | 7187 | 3217 | 18 |
| H17A | 9821 | 10020 | 2879 | 21 |
| H17B | 10561 | 8587 | 3686 | 21 |
| H18A | 10243 | 8732 | 621 | 32 |
| H18B | 10897 | 7252 | 1416 | 32 |
| H18C | 11947 | 8720 | 1212 | 32 |

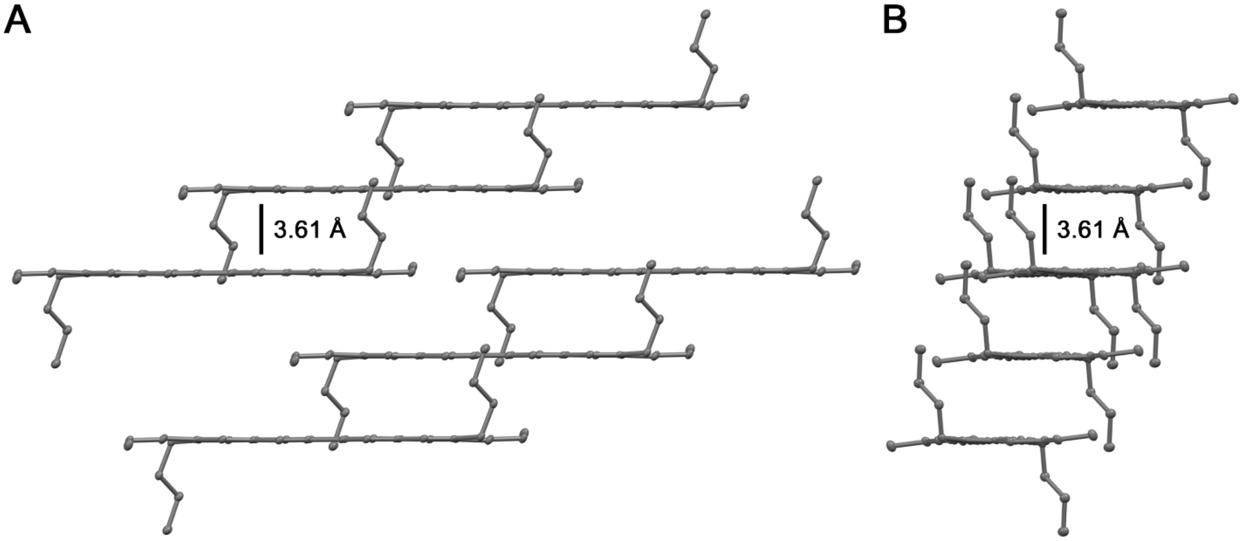


**Figure S78.** (A) Solid-state packing of compound **8** viewed down the short molecular axis and (B) viewed down the long molecular axis. Hydrogens are omitted for clarity, thermal ellipsoids shown at the 50% probability level.

## H. UV/Vis and Fluorescence Spectroscopy

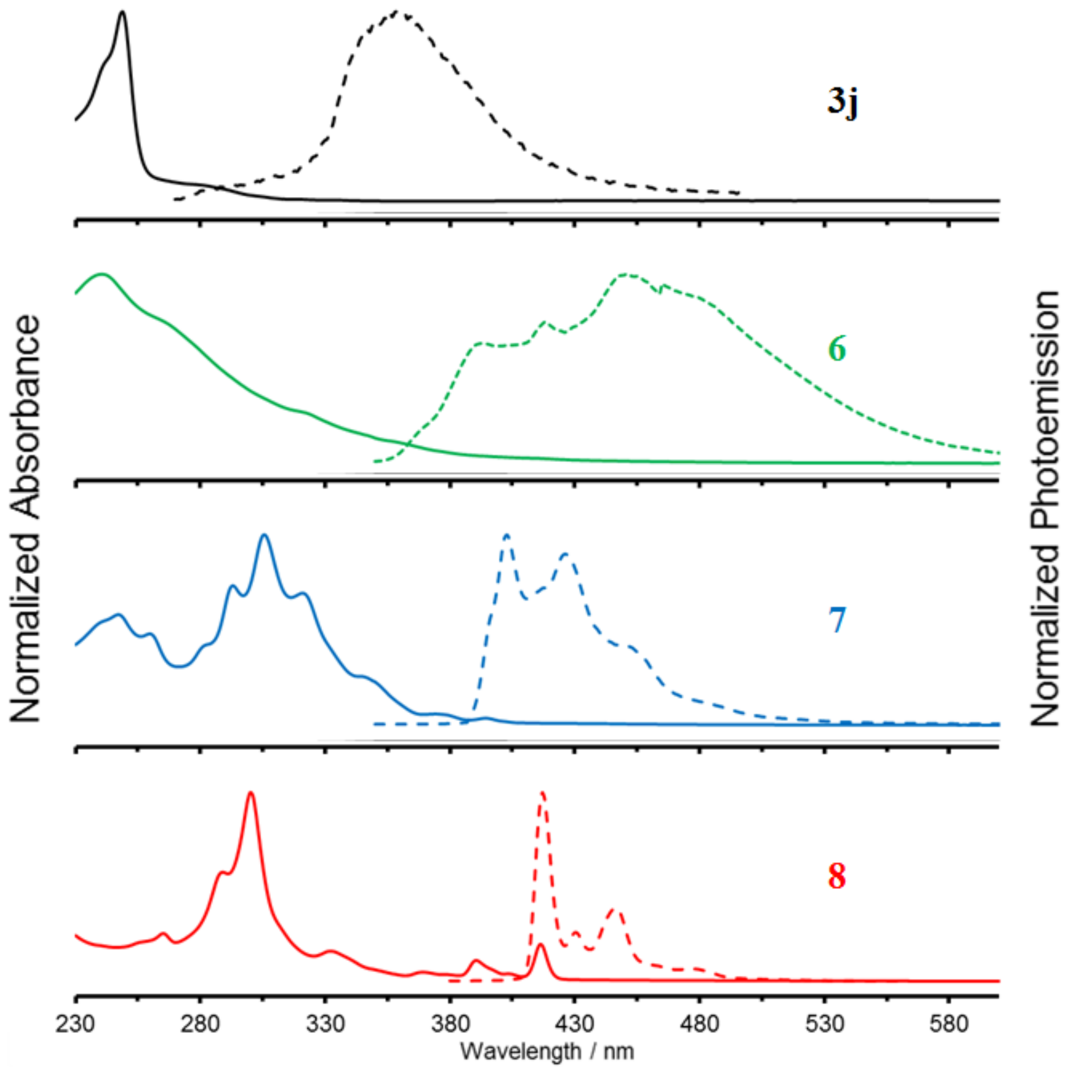


**Figure S79**: UV/Vis absorption (solid) and photoemission (dashed) of **3j** (black), **6** (green), **7** (blue), and **8** (red) at 5.0 μg/mL in $CH_2Cl_2$. The spectra are normalized to the $\lambda_{max}$ of each compound ($\lambda_{max}$ = 249 nm and ε = 1.0 x $10^5$ $M^{-1}cm^{-1}$ for **3j**; $\lambda_{max}$ = 240 nm and ε = 3.0 x $10^4$ $M^{-1}cm^{-1}$ for **6**; $\lambda_{max}$ = 306 nm and ε = 9.7 x $10^4$ $M^{-1}cm^{-1}$ for **7**; $\lambda_{max}$ = 301 nm and ε = 1.3 x $10^5$ $M^{-1}cm^{-1}$ for **8**). Photoemission spectrum for each compound is normalized to the $\lambda_{em}$ of each compound ($\lambda_{ex}$ = 260 nm for **3j**; $\lambda_{ex}$ = 340 nm for **6**; $\lambda_{ex}$ = 330 nm for **7**; $\lambda_{ex}$ = 370 nm for **8**).

## I. References for Supporting Information


1. D. Lehnherr, J. M. Alzola, E. B. Lobkovsky, W. R. Dichtel, *Chem. Eur. J.* **2015**, *21*, 18122–18127
2. C. Amatore, E. Blart, J. P. Genet, A. Jutand, S. Lemaire-Audoire, M Savignac*, J. Org. Chem.*, **1995**, *60*, 6829–6839
3. X. Zhang, Y. Zhang, J. Huang, R. P. Hsung, K. C. M. Kurtz, J. Oppenheimer, M. E. Petersen, I. K. Sagamanova, L. Shen, M. R. Tracey, *J. Org. Chem*. **2006**, *71*, 4170-4177.
4. Chen, X. Y.; Wang, L.; Frings, M.; Bolm, C. *Org. Lett.* **2014**, *16*, 3796–3799.
5. T. Kitamura, N. Fukatzu, Y. Fujiwara, *J. Org. Chem.*, **1998**, *63*, 8579–8581
6. Doddrell, D.; Barfield, M.; Adcock, W.; Aurangzeb, M.; Jordan, D. *J. Chem. Soc., Perkin Trans.* **1976**, *4*, 402–412.
7. Gaussian 09, Revision D.01, M. J. Frisch, G. W. Trucks, H. B. Schlegel, G. E. Scuseria, M. A. Robb, J. R. Cheeseman, G. Scalmani, V. Barone, B. Mennucci, G. A. Petersson, H. Nakatsuji, M. Caricato, X. Li, H. P. Hratchian, A. F. Izmaylov, J. Bloino, G. Zheng, J. L. Sonnenberg, M. Hada, M. Ehara, K. Toyota, R. Fukuda, J. Hasegawa, M. Ishida, T. Nakajima, Y. Honda, O. Kitao, H. Nakai, T. Vreven, J. A. Montgomery, Jr., J. E. Peralta, F. Ogliaro, M. Bearpark, J. J. Heyd, E. Brothers, K. N. Kudin, V. N. Staroverov, T. Keith, R. Kobayashi, J. Normand, K. Raghavachari, A. Rendell, J. C. Burant, S. S. Iyengar, J. Tomasi, M. Cossi, N. Rega, J. M. Millam, M. Klene, J. E. Knox, J. B. Cross, V. Bakken, C. Adamo, J. Jaramillo, R. Gomperts, R. E. Stratmann, O. Yazyev, A. J. Austin, R. Cammi, C. Pomelli, J. W. Ochterski, R. L. Martin, K. Morokuma, V. G. Zakrzewski, G. A. Voth, P. Salvador, J. J. Dannenberg, S. Dapprich, A. D. Daniels, O. Farkas, J. B. Foresman, J. V. Ortiz, J. Cioslowski, and D. J. Fox, Gaussian, Inc., Wallingford CT, **2013**.
8. CYLview, 1.0b; Legault, C. Y., Université de Sherbrooke, **2009** (http://www.cylview.org).
